# Metal-free one-pot synthesis of 2-substituted and 2,3-disubstituted morpholines from aziridines

**DOI:** 10.3762/bjoc.11.59

**Published:** 2015-04-22

**Authors:** Hongnan Sun, Binbin Huang, Run Lin, Chao Yang, Wujiong Xia

**Affiliations:** 1 State Key Laboratory of Urban Water Resource and Environment, the Academy of Fundamental and Interdisciplinary Sciences, Harbin Institute of Technology, Harbin 150080, P. R. China

**Keywords:** ammonium persulfate, aziridine, metal free, morpholine

## Abstract

The metal-free synthesis of 2-substituted and 2,3-disubstituted morpholines through a one-pot strategy is described. A simple and inexpensive ammonium persulfate salt enables the reaction of aziridines with halogenated alcohols to proceed via an S_N_2-type ring opening followed by cyclization of the resulting haloalkoxy amine.

## Introduction

**Figure 1:**
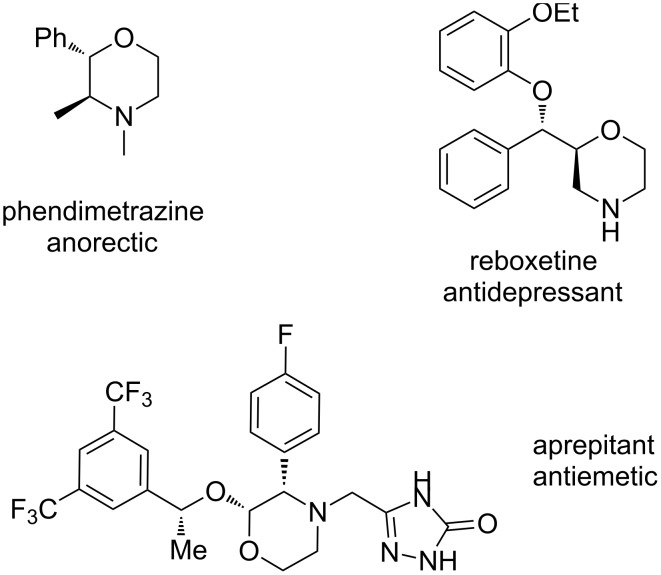
Pharmaceutically active 2- and 2,3-disubstituted morpholines.

Morpholines are common structural cores of a broad range of biological and pharmacological natural or synthetically important organic molecules [1]. In particular, a number of 2-substituted and 2,3-disubstituted morpholines are clinically available drugs (Figure 1). For example, the *trans*-2,3-disubstituted morpholine, phendimetrazine (bontril), is a clinically available appetite suppressant [2], the 2-substituted morpholine, reboxetine, is a clinically active, efficacious, and well-tolerated antidepressant drug [3–5], and the *cis*-2,3-disubstituted morpholine, aprepitant, is approved for the use in the prevention of chemotherapy-induced nausea and vomiting [6].

**Scheme 1:**
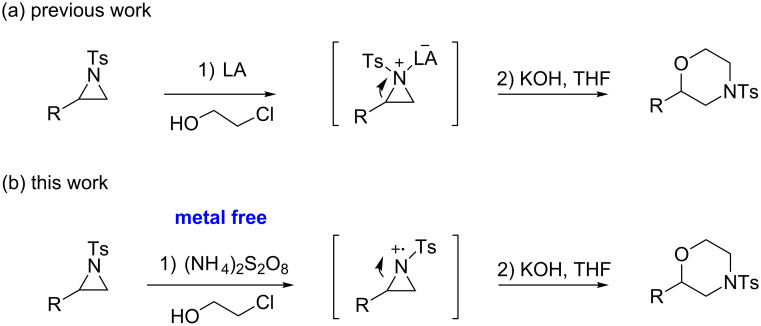
One-pot synthesis of morpholines through ring opening of aziridines with haloalcohols.

In addition to pharmacological properties, morpholines are also used in organic synthesis as bases, catalysts, and chiral auxiliaries [7–13]. Thus, up to now, numerous strategies toward the synthesis of substituted morpholines have been reported [14–23]. Despite these advances, the synthetic approach to 2-substituted and 2,3-disubstituted morpholines is still scarce. Recently, Ghorai and co-workers disclosed an intriguing strategy for the synthesis of substituted morpholines through Cu(OTf)_2_-catalyzed ring-opening/closing reactions of aziridines and halogenated alcohols in high yield and enantioselectivity (Scheme 1a) [21]. However, this method suffered from the need for transition metal catalysts and low temperatures in the initial stage. Thus, the discovery of an operationally simple and eco-friendly synthetic approach is a desirable complement to current methodologies.

Recently we have reported the visible light-mediated ring opening of aziridines by a number of nucleophiles, such as LiBr, NaN_3_ and alcohols [24]. As a part of an ongoing program on the ring opening of aziridines [25–31], we have developed an efficient method for the synthesis of 2-substituted and 2,3-disubstituted morpholines from aziridines utilizing a simple and inexpensive ammonium persulfate salt as the oxidant at room temperature (Scheme 1b) [32–33].

## Results and Discussion

Our investigation started with the treatment of 2-phenyl-*N*-tosylaziridine (**1a**) with 2-chloroethanol in the presence of sodium persulfate at room temperature for 13 h (Table 1). To our delight, NMR studies showed that chloroethoxyamine **2a** is observed as the only ring-opening product. After screening different persulfates, in concordance with Zeng [32], we found that ammonium persulfate ((NH_4_)_2_S_2_O_8_) is superior to Na_2_S_2_O_8_ and K_2_S_2_O_8_ in the transformation, leading to chloroethoxyamine **2a** in an excellent yield (93%) in short time.

**Table 1:** Metal-free ring opening of 2-phenyl-*N*-tosylaziridine (**1a**) with 2-chloroethanol using different persulfates as oxidant.^a^

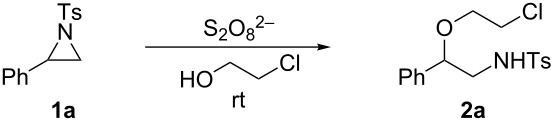			

Entry	S_2_O_8_^2−^	Time (h)	Yield (%)^b^

1	Na_2_S_2_O_8_	13	94
2	K_2_S_2_O_8_	16	96
3	(NH_4_)_2_S_2_O_8_	0.5	93

^a^Aziridine **1a** (0.3 mmol), (NH_4_)_2_S_2_O_8_ (0.6 mmol, 2 equiv) in 2-chloroethanol (10 equiv) as the solvent; ^b^isolated yield.

**Scheme 2:**
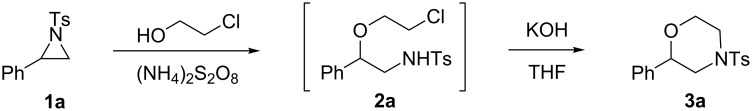
Metal-free one-pot synthesis of morpholine **3a** from aziridine **1a**.

Encouraged by the result that treatment of **2a** with KOH at room temperature in THF led to morpholine **3a** in 90% yield, we performed the reaction by addition of KOH to the mixture of **1a** and (NH_4_)_2_S_2_O_8_ in 2-chloroethanol after the reaction and hoped to prepare **3a** in one pot. Gratifyingly the reaction proceeded smoothly to furnish **3a** in 93% yield (Scheme 2).

To investigate the scope of this methodology, various substituted aziridines were prepared from the corresponding alkenes and submitted them to the reaction conditions. As shown in Table 2, both electron-deficient and electron-rich 2-aryl-substituted aziridines **1a**–**j** were well tolerated and the desired morpholines **3a**–**j** were obtained in good yields (Table 2, entries 1–10). *N*-Tosylaziridine **1k** was also a viable substrate for the reaction leading to the corresponding bicyclic morpholine **3k** in 95% yield (Table 2, entry 11). In addition, the reaction of 2,3-disubstituted aziridines (acyclic and/or cyclic ones), separable mixtures of regioisomers **3l**,**m** and **4l**,**m** were obtained arising from isomeric ring opening (Table 2, entries 12 and 13). We speculated that the observed regioselectivity might depend on the combined action of electronic effects and the position of substitution [31]. Under identical reaction conditions, the separable 2-butylmorpholine **3n** and 3-butylmorpholine **4n** could be easily prepared from aziridine **1n** (Table 2, entry 14).

**Table 2:** Metal-free one-pot synthesis of morpholines from aziridines.^a^

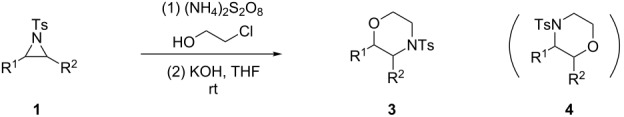			

Entry	Substrate	Morpholine Yield (%)^b^	

	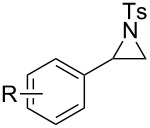	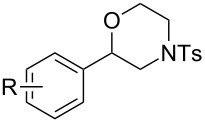	
1	**1a**, R = H	**3a**, 93	
2	**1b**, R = 3-OMe	**3b**, 90	
3	**1c**, R = 4-Me	**3c**, 88	
4	**1d**, R = 4-*t*-Bu	**3d**, 84	
5	**1e**, R = 4-F	**3e**, 84	
6	**1f**, R = 4-Cl	**3f**, 80	
7	**1g**, R = 2-Cl	**3g**, 87	
8	**1h**, R = 4-CF_3_	**3h**, 78	
9	**1i**, R = 4-NO_2_	**3i**, 83	
10	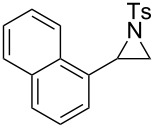 **1j**	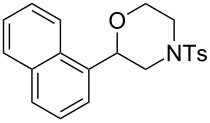 **3j**, 82	
11	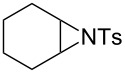 **1k**	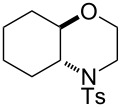 **3k**, 95	
12	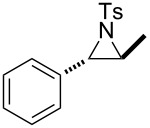 **1l**	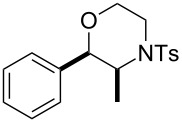 **3l**, 75	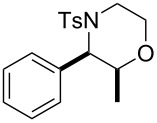 **4l**, 16
13	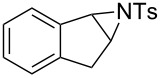 **1m**	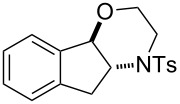 **3m**, 40	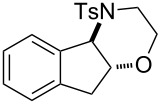 **4m**, 47
14	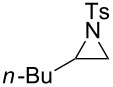 **1n**	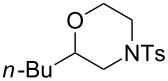 **3n**, 45	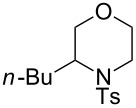 **4n**, 30

^a^In all cases 2-chloroethanol served as the solvent; ^b^isolated yield.

**Scheme 3:**
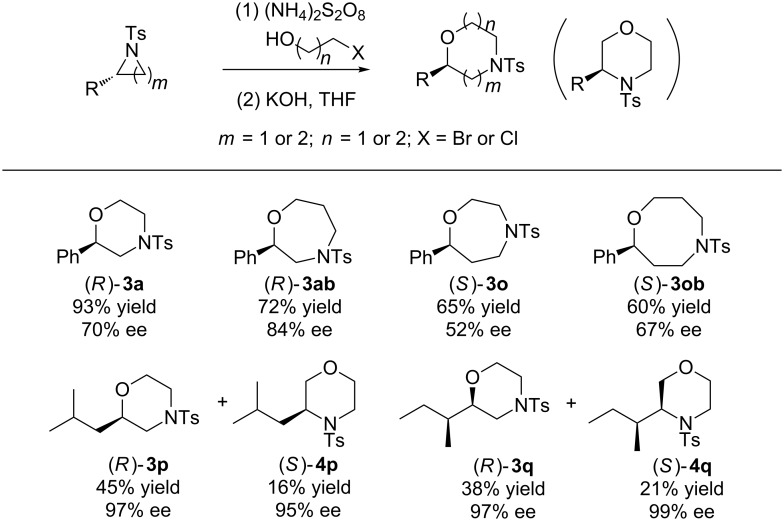
Metal-free one-pot synthesis of optically pure morpholine derivatives from chiral aziridines.

To further investigate the applicability of this strategy in organic synthesis, we next performed a series of experiments to determine the potential of the straightforward synthesis of optically pure morpholines from chiral aziridines. The initial investigation was carried out by the replacement of racemic 2-phenyl-*N*-tosylaziridine (**1a**) with optically pure (*S*)-2-phenyl-1-tosylaziridine under the standard reaction conditions. To our delight, (*R*)-**3a** was obtained in 93% yield and 70% ee (Scheme 3). For optically pure (*S*)-2-alkyl-substituted aziridines **1p**,**q**, separable (*R*)-2-alkylmorpholines **3p**,**q** and (*S*)-3-alkylmorpholines **4p**,**q** were prepared in pure forms (95–99% ee) and low to moderate overall yields. Furthermore, the enantiospecific synthesis of seven and eight-membered homologues of morpholine was also conducted to extend the potential application of the strategy. For example, when 2-chloroethanol was replaced by 3-bromopropanol, the seven-membered product (*R*)-**3ab** was obtained in 72% yield and 84% ee. Similarly, reaction of (*R*)-2-phenyl-*N*-tosylazetidine (**1o**) with 2-bromoethanol and/or 3-bromopropanol under the one-pot reaction conditions, afforded the seven-membered product (*S*)-**3o** and the eight-membered compound (*S*)-**3ob** in 65% and 60% yield with 52% and 67% ee, respectively.

**Scheme 4:**
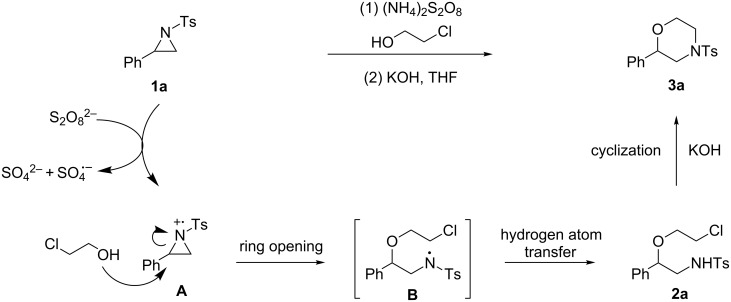
Proposed mechanism.

Based on the above results, a viable mechanism was proposed as shown in Scheme 4. Initially, aziridine **1a** might participate in single-electron transfer (SET) with the persulfate anion to render the radical cation **A** [32,34]. Concerted ring opening and nucleophilic addition leads to amino radical intermediate **B**, which is converted to the haloalkoxy amine intermediate **2a** after abstraction of one hydrogen atom from alcohol. Finally, an intramolecular ring closure affords the morpholine product **3a** in the presence of KOH [21].

## Conclusion

In conclusion, we have developed a simple and practicable metal-free protocol for the synthesis of 2-substituted and 2,3-disubstituted morpholines. Compared with the previous procedure, this reaction is conducted with a simple and inexpensive ammonium persulfate salt as the oxidant to realize the ring opening of aziridines for the reaction with haloalcohols through a radical cation intermediate pathway. Furthermore, a range of optically pure morpholines could be achieved by the use of chiral aziridines.

## Experimental

**General procedure for the one-pot synthesis of morpholines:** A 10 mL round bottom flask equipped with a magnetic stirring bar was charged with aziridine/azetidine **1** (0.3 mmol, 1 equiv), (NH_4_)_2_S_2_O_8_ (137 mg, 0.6 mmol, 2 equiv) and haloalcohol (10 equiv). The mixture was stirred at rt for the appropriate time until the starting material disappeared completely (monitored by TLC). Then, 5.0 mL THF and excess KOH (12 equiv) were added to the reaction mixture and the mixture was stirred at rt. After the reaction was completed, the resulting suspension was quenched with saturated aqueous sodium bicarbonate solution and extracted with ethyl acetate. The organic layers were combined, washed with brine and dried over anhydrous sodium sulfate. Solvents were removed under reduced pressure and the residue was purified by column chromatography on silica gel using ethyl acetate/hexane mixtures to afford the pure products.

## Supporting Information

File 1 Experimental procedures, characterization data and copies of ^1^H and ^13^C NMR spectra for products.
