# Supplementary material for: Metal-free one-pot synthesis of 2-substituted and 2,3-disubstituted morpholines from aziridines
Source: Beilstein J Org Chem. 2015 Apr 22;11:524–9. doi: 10.3762/bjoc.11.59 (PMC4419545; doi:10.3762/bjoc.11.59)

Supporting Information

for

Metal-free one-pot synthesis of 2-substituted and 2,3-disubstituted morpholines from aziridines

Hongnan Sun, Binbin Huang, Run Lin, Chao Yang and Wujiong Xia*

Address: State Key Laboratory of Urban Water Resource and Environment, the Academy of Fundamental and Interdisciplinary Sciences, Harbin Institute of Technology, Harbin 150080, P. R. China

Email: Wujiong Xia* - xiawj@hit.edu.cn

* Corresponding author

Experimental procedures, characterization data and copies of ^{1}H and ^{13}C NMR spectra for products

Contents

1. General information S2

2.1 General procedure for the synthesis of aziridines **1a–n** S2

2.2 General procedure for the synthesis of chiral aziridines **1a**, **1p** and **1q** S5

2.3 Procedure for the synthesis of chiral azetidine **1o** S6

3. General procedure for one-pot synthesis of morpholine derivatives and characterization data of corresponding products S6

4. Copies of HPLC chromatograms for ee determination S16

5. Copies of NMR spectra S22

1. General information

All reagents were purchased from commercial sources unless otherwise noted. Solvent was freshly distilled prior to use unless otherwise noted. Reactions were monitored by thin layer chromatography (TLC) and visualized by UV light (254 nm) or by treatment with a solution of 10 g phosphomolybdic acid and 100 mL EtOH followed by heating. ^{1}H NMR (400 MHz) and ^{13}C NMR (100 MHz) spectra were obtained on Bruker AV-400 instrument. Chemical shifts δ for ^{1}H NMR spectra were reported in ppm referenced to an internal $SiMe_4$ standard. Chemical shifts δ for ^{13}C NMR spectra were reported in ppm relative to the center line signal of the $CDCl_3$ triplet at 77.0 ppm. HRMS spectra were recorded on a Bruker Esquire LC mass spectrometer using electrospray ionization (ESI). Enantiomeric excess (ee) was determined by HPLC using chiralpak AS-H or chiralcel OD-H analytical column (detection at 254 nm).

2.1 General procedure for the synthesis of aziridines 1a–n

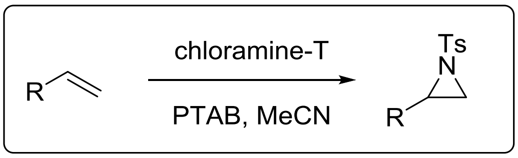

The compounds were prepared in a similar manner as described before[1]. To a mixture of the appropriate olefin (3 mmol) and $TsNClNa\cdot 3H_2O$ (0.93 g, 3.3 mmol) in 15 mL of MeCN, was added phenyltrimethylammonium tribromide (PTAB) (0.113 g, 0.3 mmol) at rt. After 12 h vigorous stirring, the reaction mixture was concentrated and filtered through a short column of silica gel eluting with EtOAc. After evaporation of the solvent, the resultant solid was purified by flash column chromatography to yield the corresponding aziridine.

1 Jeong, J. U.; Tao, B.; Sagasser, I.; Henniges, H.; Sharpless, K. B. *J. Am. Chem. Soc.* **1998**, 120, 6844-6845.

2-Phenyl-1-tosylaziridine (1a).[2] ^{1}H NMR (400 MHz, $CDCl_3$): δ 7.86 (d, *J* = 8.2 Hz, 2H), 7.33-7.20 (m, 7H), 3.77 (dd, *J* = 4.5, 7.1 Hz, 1H), 2.97 (d, *J* = 7.2 Hz, 1H), 2.42 (s, 3H), 2.38 (d, *J* = 4.4 Hz, 1H).

2-(3-Methoxyphenyl)-1-tosylaziridine (1b).[3] ^{1}H NMR (400 MHz, $CDCl_3$): δ 7.87 (d, *J* = 8.2 Hz, 2H), 7.33 (d, *J* = 8.2 Hz, 2H), 7.20 (t, *J* = 7.9 Hz, 1H), 6.82-6.79 (m, 2H), 6.72 (s, 1H), 3.75 (s, 3H), 3.75 – 3.72 (m, 1H), 2.97 (d, *J* = 7.2 Hz, 1H), 2.43 (s, 3H), 2.38 (d, *J* = 4.4 Hz, 1H).

2-(*p*-Tolyl)-1-tosylaziridine (1c).[2] ^{1}H NMR (400 MHz, $CDCl_3$): δ 7.86 (d, *J* = 8.3 Hz, 2H), 7.32 (d, *J* = 8.1 Hz, 2H), 7.09 (s, 4H), 3.74 (dd, *J* = 7.2, 4.5 Hz, 1H), 2.96 (d, *J* = 7.2 Hz, 1H), 2.42 (s, 3h), 2.37 (d, *J* = 4.5 Hz, 1H), 2.30 (s, 3H).

2-(4-(*tert*-Butyl)phenyl)-1-tosylaziridine (1d).[2] ^{1}H NMR (400 MHz, $CDCl_3$): δ 7.87 (d, *J* = 8.3 Hz, 2H), 7.34-7.30 (m, 4H), 7.14 (d, *J* = 8.3 Hz, 2H), 3.76 (dd, *J* = 7.2, 4.5 Hz, 1H), 2.96 (d, *J* = 7.2 Hz, 1H), 2.43 (s, 3H), 2.38 (d, *J* = 4.5 Hz, 1H), 1.28 (s, 9H).

2-(4-Fluorophenyl)-1-tosylaziridine (1e).[2] ^{1}H NMR (400 MHz, $CDCl_3$): δ 7.86 (d, *J* = 8.3 Hz, 2H), 7.34 (d, *J* = 8.1 Hz, 2H), 7.21-6.96 (m, 4H), 3.75 (dd, *J* = 4.5, 7.1 Hz, 1H), 2.97 (d, *J* = 7.1 Hz, 1H), 2.44 (s, 3H), 2.35 (d, *J* = 4.5 Hz, 1H).

2-(4-Chlorophenyl)-1-tosylaziridine (1f).[2] ^{1}H NMR (400 MHz, $CDCl_3$): δ 7.85 (d, *J* = 8.3 Hz, 2H), 7.33 (d, *J* = 8.1 Hz, 2H), 7.27-7.25 (m, 2H), 7.15 (d, *J* = 8.5 Hz, 2H), 3.73 (dd, *J* = 7.1, 4.4 Hz, 1H), 2.98 (d, *J* = 7.2 Hz, 1H), 2.43 (s, 3H), 2.34 (d, *J* = 4.4 Hz, 1H).

2-(2-Chlorophenyl)-1-tosylaziridine (1g).[4] ^{1}H NMR (400 MHz, $CDCl_3$): δ 7.90 (d, *J* = 8.3 Hz, 2H), 7.36-7.32 (m, 3H), 7.23-7.16 (m, 3H), 4.04 (dd, *J* = 4.4, 7.2 Hz, 1H), 3.03 (d, *J* = 7.2 Hz, 1H), 2.45 (s, 3H), 2.29 (d, *J* = 4.4 Hz, 1H).

---

[2] Gao, G.-Y.; Harden, J. D.; Zhang, X. P. *Org. Lett.* **2005**, *7*, 3191–3193.
[3] Huang, C.-Y.; doyle, A. G. *J. Am. Chem. Soc.* **2012**, 134, 9541-9544.
[4] Craig, Ⅱ, R. A.; O`Connor, N. R.; Goldberg, A. F. G.; Stoltz, B. M. *Chem. Eur. J.* **2014**, 20, 4806-4813.

1-Tosyl-2-(4-(trifluoromethyl)phenyl)aziridine (1h).[3] ^{1}H NMR (400 MHz, $CDCl_3$): δ7.87 (d, *J* = 8.3 Hz, 2H), 7.55 (d, *J* = 8.2 Hz, 2H), 7.34 (d, *J* = 8.1 Hz, 2H), 3.81 (dd, *J* = 7.1, 4.3 Hz, 1H), 3.02 (d, *J* = 7.2 Hz, 1H), 2.44 (s, 3H), 2.37 (d, *J* = 4.3 Hz, 1H).

2-(4-Nitrophenyl)-1-tosylaziridine (1i).[5] ^{1}H NMR (400 MHz, $CDCl_3$): δ 8.15 (d, *J* = 8.7 Hz, 2H), 7.87 (d, *J* = 8.3 Hz, 2H), 7.41 (d, *J* = 8.7 Hz, 2H), 7.36 (d, *J* = 8.1 Hz, 2H), 3.84 (dd, *J* = 7.1, 4.3 Hz, 1H), 3.05 (d, *J* = 7.2 Hz, 1H), 2.45 (s, 3H), 2.38 (d, *J* = 4.2 Hz, 1H).

2-(Naphthalen-1-yl)-1-tosylaziridine (1j). ^{1}H NMR (400 MHz, $CDCl_3$): δ 8.14 (d, *J* = 8.0 Hz, 1H), 7.94 (d, *J* = 8.3 Hz, 2H), 7.86-7.84 (m, 1H), 7.82-7.75 (m, 1H), 7.56-7.49 (m, 2H), 7.36-7.33 (m, 4H), 4.33 (dd, *J* = 7.1, 4.6 Hz, 1H), 3.14 (d, *J* = 7.2 Hz, 1H), 2.45-2.41 (m, 4H). ^{13}C NMR (100 MHz, $CDCl_3$): δ 144.7, 134.9, 133.3, 131.5, 130.9, 129.8, 128.7, 128.6, 128.1, 126.6, 126.1, 125.3, 124.3, 122.9, 39.5, 35.1, 21.6. HRMS-ESI (*m/z*): $[M+H]^+$ calculated for $C_{19}H_{18}NO_2S$, 324.1058; found: 324.1058.

7-Tosyl-7-azabicyclo[4.1.0]heptane (1k).[3] ^{1}H NMR (400 MHz, $CDCl_3$): δ 7.81 (d, *J* = 8.3 Hz, 2H), 7.32 (d, *J* = 8.1 Hz, 2H), 2.99-2.95 (m, 2H), 2.44 (s, 3H), 1.80-1.77 (m, 4H), 1.45-1.36 (m, 2H), 1.27-1.17 (m, 2H).

***trans*-2-Methyl-3-phenyl-1-tosylaziridine (1l).**[6] ^{1}H NMR (400 MHz, $CDCl_3$): δ 7.82 (d, *J* = 7.8 Hz, 2H), 7.26-7.13 (m, 7H), 3.79 (d, *J* = 4.1 Hz, 1H), 2.93-2.88 (m, 1H), 2.38 (s, 3H), 1.84 (d, *J* = 5.9 Hz, 3H).

1-Tosyl-1,1a,6,6a-tetrahydroindeno[1,2-*b*]azirine (1m).[7] ^{1}H NMR (400 MHz, $CDCl_3$): δ7.83 (d, *J* = 8.3 Hz, 2H), 7.40 (d, *J* = 7.3 Hz, 1H), 7.31 (d, *J* = 8.1 Hz, 2H), 7.23-7.17 (m, 3H), 4.30 (d, *J* = 5.3 Hz, 1H), 3.91-3.88 (m, 1H), 3.14-3.13 (m, 2H), 2.41 (s, 3H).

---

[5] Evans, D. A., Faul, M. M., Bilodeau, M. T. *J. Am. Chem. Soc.* **1994**, 116, 2742-2753.
[6] Yoshimura, A.; Nemykin, V. N.; Zhdankin, V. V. *Chem. Eur. J.* **2011**, 17, 10538-10541.
[7] Saikia, I.; Kashyap, B.; Phukan, P. *Chem. Commun.* **2011**, *47*, 2967–2969.

2-Butyl-1-tosylaziridine (1n).[8] ^{1}H NMR (400 MHz, $CDCl_3$): δ 7.83 (d, J = 8.3 Hz, 2H), 7.34 (d, J = 8.1 Hz, 2H), 2.75-2.69 (m, 1H), 2.63 (d, J = 7.0 Hz, 1H), 2.06 (s, 3H), 2.06 (d, J = 4.6 Hz, 1H), 1.59-1.5 (m, 1H), 1.38-1.20 (m, 5H), 0.81 (t, J = 7.0 Hz, 3H).

2.2 General procedure for the synthesis of chiral aziridines 1a, 1p and 1q

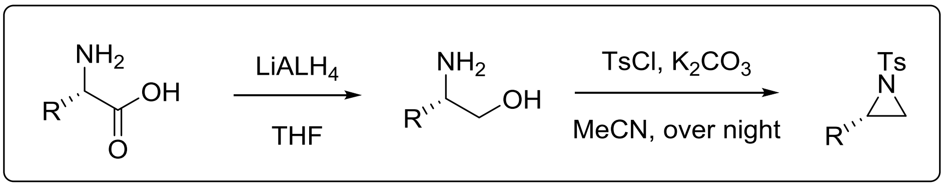

The compounds were prepared in a similar manner as described before[9]. The chiral amino acid was reduced to the amino alcohol by treatment with $LiAlH_4$ in THF. The reaction was quenched with water. The solid was filtered off and the filtrate was dried over anhydrous sodium sulfate. The solvent was removed under reduced pressure to give the crude product. Subsequent treatment of the crude product with TsCl and K_2CO_3 in MeCN afforded pure chiral aziridines.

(*S*)-2-Isobutyl-1-tosylaziridine (1p).[10] ^{1}H NMR (400 MHz, $CDCl_3$): δ 7.83 (d, J = 8.2 Hz, 2H), 7.33 (d, J = 8.1 Hz, 2H), 2.82-2.76 (m, 1H), 2.63 (d, J = 7.0 Hz, 1H), 2.44 (s, 3H), 2.02 (d, J = 4.6 Hz, 1H), 1.67-1.57 (m, 1H), 1.39-1.28 (m, 2H), 0.88 (dd, J = 6.6, 1.9 Hz, 6H). ^{13}C NMR (100 MHz, $CDCl_3$): δ 144.4, 135.2, 129.6, 127.9, 40.4, 39.0, 34.0, 26.7, 22.7, 21.9, 21.6. HRMS-ESI (*m*/*z*): $[M+H]^+$ calculated for $C_{13}H_{20}NO_2S$, 254.1215; found: 254.1215.

(*S*)-2-((*S*)-*sec*-Butyl)-1-tosylaziridine (1q).[11] ^{1}H NMR (400 MHz, $CDCl_3$): δ 7.83 (d, J = 8.2 Hz, 2H), 7.33 (d, J = 8.1 Hz, 2H), 2.61-2.55 (m, 2H), 2.44 (s, 3H), 2.06 (d, J = 3.3 Hz, 1H), 1.42-1.33 (m, 1H), 1.20-1.07 (m, 2H), 0.88 (d, J = 6.6 Hz, 3H), 0.81 (t,

[8] Kumar, G. D. K.; Baskaran, S. *Chem. Commun.* **2004**, 1026–1027.
[9] Cernerud, M.; Adolfsson, H.; Moberg, C. *Tetrahedron: Asymmetry* 1997, 8, 2655-2662.
[10] Craven, A. P.; Dyke, H. J.; Thomas, E. J. *Tetrahedron* **1989**, 45, 2417-2429.
[11] Kolb, H. C.; Kanamarlapudi, R. C.; Richardson, P. F.; Khan, G. U. S. Pat. Appl. 20030153771.

J = 7.3 Hz, 3H). ^{13}C NMR (100 MHz, CDCl$_3$): δ 144.4, 135.1, 129.5, 128.0, 45.1, 36.6, 32.6, 27.1, 21.6, 15.5, 10.8. HRMS-ESI (*m/z*): [M+H]$^+$ calculated for $C_{13}H_{20}NO_2S$, 254.1215; found: 254.1215.

2.3 Procedure for the synthesis of chiral azetidine 1o

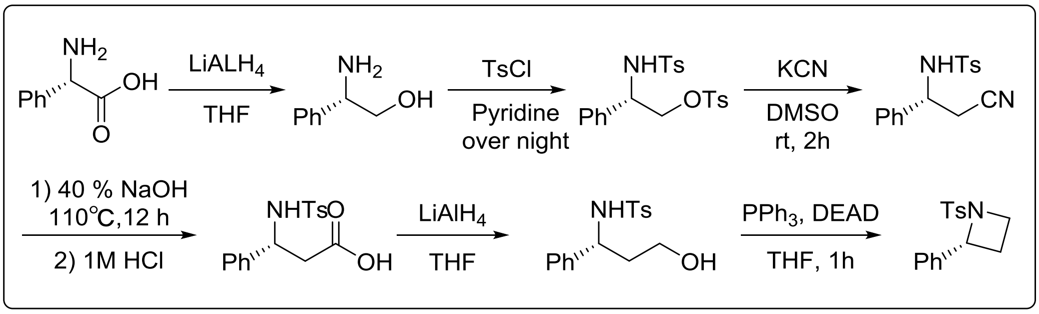

The compound was prepared in a similar manner as described before[12]. The amino acid was reduced to the corresponding amino alcohol by treatment with $LiAlH_4$ in THF. Tosylation of the amino alcohol with 2 equiv TsCl in pyridine followed by treatment with KCN in DMSO gave the aminonitrile. The nitrile was hydrolyzed to the corresponding acid by treatment with 40% aq NaOH under reflux. Subsequent reduction of the acid using $LiAlH_4$ in THF afforded the amino alcohol. Finally, the alcohol was cyclized to the corresponding azetidine by treatment with PPh_3 and DEAD in THF.

(*R*)-2-Phenyl-1-tosylazetidine (1o).[12] ^{1}H NMR (400 MHz, CDCl$_3$): δ 7.68 (d, J = 7.7 Hz, 2H), 7.41 (d, J = 7.2 Hz, 2H), 7.37-7.26 (m, 5H), 4.88 (t, J = 8.2 Hz, 1H), 3.81-3.72 (m, 2H), 2.44 (s, 3H), 2.35-2.28 (m, 1H), 2.26-2.14 (m, 1H).

3. General procedure for one-pot synthesis of morpholine derivatives and characterization data of the corresponding product

General procedure: A 10 mL round bottomed flask equipped with a magnetic stir bar was charged with aziridine/azetidine **1** (0.3 mmol, 1 equiv), $(NH_4)_2S_2O_8$ (137 mg, 0.6 mmol, 2 equiv) and haloalcohol (10 equiv). The mixture was stirred at rt for the

[12] Ghorai, M. K.; Das, K.; Kumar, A. *Tetrahedron Lett.* **2007**, 48, 2471-2475.

appropriate time (until the starting material disappeared completely as monitored by TLC). Then, 5.0 mL THF and excess KOH (12 equiv) were added to the mixture and stirring was continued at rt. After completion of the reaction, the resulting suspension was treated with saturated aqueous sodium bicarbonate solution and extracted with ethyl acetate. The organic layers were combined, washed with brine and dried over anhydrous sodium sulfate. The solvent was removed under reduced pressure and the residue was purified by column chromatography on silica gel using ethyl acetate/hexane as eluent to afford the pure product.

2-Phenyl-4-tosylmorpholine (3a). To a 10 mL round bottomed flask was added **1a** (82 mg, 0.3 mmol), $(NH_4)_2S_2O_8$ (137 mg, 0.6 mmol) and chloroethanol (240 mg, 3.0 mmol). The mixture was stirred at rt for 0.5 h. THF (5.0 mL) and KOH (202 mg, 3.6 mmol) were then added and stirred at rt for further 1 h to afford **3a** as a white solid (88 mg, 93% yield). ^{1}H NMR (400 MHz, $CDCl_3$): δ 7.61 (d, J = 8.3 Hz, 2H), 7.36-7.28 (m, 7H), 4.60 (dd, J = 2.6, 10.3 Hz, 1H), 4.09-4.05 (m, 1H), 3.85 (dt, J = 2.7, 11.6 Hz, 1H), 3.76 (td, J = 2.2, 11.5 Hz, 1H), 3.65-3.62 (m, 1H), 2.50 (dt, J = 3.4, 11.6 Hz, 1H), 2.43 (s, 3H), 2.24 (dd, J = 10.4, 11.5 Hz, 1H). ^{13}C NMR (100 MHz, $CDCl_3$): δ 143.9, 138.7, 132.2, 129.8, 128.5, 128.3, 127.8, 126.0, 77.4, 66.2, 51.9, 45.4, 21.5. HRMS-ESI (*m*/*z*): $[M+H]^+$ calculated for $C_{17}H_{20}NO_3S$, 318.1164; found: 318.1156.

2-(3-Methoxyphenyl)-4-tosylmorpholine (3b). To a 10 mL round bottomed flask was added **1b** (91 mg, 0.3 mmol), $(NH_4)_2S_2O_8$ (137 mg, 0.6 mmol) and chloroethanol (240 mg, 3.0 mmol). The mixture was stirred at rt for 0.5 h. THF (5.0 mL) and KOH (202 mg, 3.6 mmol) were then added and stirred at rt for further 1 h to afford **3b** as a colorless liquid (94 mg, 90% yield). ^{1}H NMR (400 MHz, $CDCl_3$): δ 7.61 (d, J = 8.2 Hz, 2H), 7.32 (d, J = 8.1 Hz, 2H), 7.26-7.23 (m, 1H), 6.89-6.82 (m, 3H), 4.57 (dd, J = 2.5, 10.2 Hz, 1H), 4.07 (dd, J = 2.2, 11.7 Hz, 1H), 3.88-3.74 (m, 5H), 3.63 (d, J = 11.4 Hz, 1H), 2.49 (dt, J = 3.4, 11.6 Hz, 1H), 2.43 (s, 3H), 2.23 (t, J = 10.9 Hz, 1H). ^{13}C NMR (100 MHz, $CDCl_3$): δ 159.7, 143.9, 140.2, 132.2, 129.8, 129.5, 127.8, 118.2, 113.8, 111.5, 77.3, 66.2, 55.3, 51.9, 45.4, 21.5. HRMS-ESI (*m*/*z*): $[M+H]^+$ calculated for $C_{18}H_{22}NO_4S$, 348.1270; found: 348.1275.

2-(*p*-Tolyl)-4-tosylmorpholine (3c). To a 10 mL round bottomed flask was added **1c**

(86 mg, 0.3 mmol), $(NH_4)_2S_2O_8$ (137 mg, 0.6 mmol) and chloroethanol (240 mg, 3.0 mmol). The mixture was stirred at rt for 0.5 h. THF (5.0 mL) and KOH (202 mg, 3.6 mmol) were then added and stirred at rt for further 1 h to afford **3c** as a white solid (87 mg, 88% yield). ^{1}H NMR (400MHz, $CDCl_3$): δ 7.61 (d, *J* = 8.2 Hz, 2H), 7.31 (d, *J* = 8.0 Hz, 2H), 7.19-7.13 (m, 4H), 4.56 (dd, *J* = 1.9, 10.2 Hz, 1H), 4.05 (dd, *J* = 2.1, 11.6 Hz, 1H), 3.84 (dt, *J* = 2.5, 11.6 Hz, 1H), 3.73 (d, *J* = 11.5 Hz, 1H), 3.62 (d, *J* = 11.4 Hz, 1H), 2.49 (dt, *J* = 3.3, 11.5 Hz, 1H), 2.43 (s, 3H), 2.33 (s, 3H), 2.23 (t, *J* = 10.9 Hz, 1H). ^{13}C NMR (100 MHz, $CDCl_3$): δ 143.9, 138.0, 135.7, 132.2, 129.7, 129.1, 127.8, 125.9, 77.3, 66.2, 52.0, 45.4, 21.5, 21.1. HRMS-ESI (*m*/*z*): $[M+H]^+$ calculated for $C_{18}H_{22}NO_3S$, 332.1320; found: 332.1318.

2-(4-(*tert*-Butyl)phenyl)-4-tosylmorpholine (3d). To a 10 mL round bottomed flask was added **1d** (99 mg, 0.3 mmol), $(NH_4)_2S_2O_8$ (137 mg, 0.6 mmol) and chloroethanol (240 mg, 3.0 mmol). The mixture was stirred at rt for 0.5 h. THF (5.0 mL) and KOH (202 mg, 3.6 mmol) were then added and stirred at rt for further 1 h to afford **3d** as a white solid (94 mg, 84% yield). ^{1}H NMR (400 MHz, $CDCl_3$): δ 7.62 (d, *J* = 8.2 Hz, 2H), 7.35 (d, *J* = 8.4 Hz, 2H), 7.32 (d, *J* = 8.0 Hz, 2H), 7.22 (d, *J* = 8.3 Hz, 2H), 4.57 (dd, *J* = 10.3, 2.5 Hz, 1H), 4.06 (dd, *J* = 11.6, 2.1 Hz, 1H), 3.84 (dt, *J* = 2.6, 11.6 Hz, 1H), 3.75 (td, *J* = 2.0, 11.6 Hz, 1H), 3.62 (d, *J* = 11.6 Hz, 1H), 2.50 (dt, *J* = 11.5, 3.4 Hz, 1H), 2.43 (s, 3H), 2.27 (dd, *J* = 11.3, 10.6 Hz, 1H), 1.30 (s, 9H). ^{13}C NMR (100 MHz, $CDCl_3$): δ 151.3, 143.9, 135.6, 132.2, 129.8, 127.8, 125.8, 125.4, 77.3, 66.2, 51.8, 45.4, 34.6, 31.3, 21.5. HRMS-ESI (*m*/*z*): $[M+H]^+$ calculated for $C_{21}H_{28}NO_3S$, 374.1790; found: 374.1795.

2-(4-Fluorophenyl)-4-tosylmorpholine (3e). To a 10 mL round bottomed flask was added **1e** (87 mg, 0.3 mmol), $(NH_4)_2S_2O_8$ (137 mg, 0.6 mmol) and chloroethanol (240 mg, 3.0 mmol) at rt for 0.5 h. THF (5.0 mL) and KOH (202 mg, 3.6 mmol) were then added and stirred at rt for further 1 h to afford **3a** as a white solid (84 mg, 84% yield). ^{1}H NMR (400 MHz, $CDCl_3$): δ 7.62 (d, *J* = 8.2 Hz, 2H), 7.33 (d, *J* = 8.1 Hz, 2H), 7.29-7.26 (m, 2H), 7.02 (t, *J* = 8.7 Hz, 2H), 4.57 (dd, *J* = 2.5, 10.2 Hz, 1H), 4.06 (dd, *J* = 2.1, 10.6 Hz, 1H), 3.84 (dt, *J* = 2.7, 11.6 Hz, 1H), 3.73 (td, *J* = 2.0, 11.5 Hz, 1H), 3.63 (d, *J* = 11.0 Hz, 1H), 2.49 (dt, *J* = 3.4, 11.6 Hz, 1H), 2.43 (s, 3H), 2.20 (dd, *J* = 10.5, 11.4 Hz, 1H). ^{13}C NMR (100 MHz, $CDCl_3$): δ 162.5 (d, *J* = 245 Hz), 144.0, 134.5 (d, *J* = 3.0 Hz), 132.2, 129.8, 127.8, 127.7 (d, *J* = 8.6 Hz), 115.4 (d, *J* = 21.3 Hz), 76.7, 66.2, 51.9, 45.3, 21.5. ^{19}F NMR (376MHz, $CDCl_3$): δ -113.6 (S)

HRMS-ESI (*m*/*z*): $[M+H]^+$ calculated for $C_{17}H_{19}FNO_3S$, 336.1070; found: 336.1076.

2-(4-Chlorophenyl)-4-tosylmorpholine (3f). To a 10 mL round bottomed flask was added **1f** (92 mg, 0.3 mmol), $(NH_4)_2S_2O_8$ (137 mg, 0.6 mmol) and chloroethanol (240 mg, 3.0 mmol) at rt for 0.5 h. THF (5.0 mL) and KOH (202 mg, 3.6 mmol) were then added and stirred at rt for further 1 h to afford **3a** as a white solid (84 mg, 80% yield). ^{1}H NMR (400 MHz, CDCl3): δ 7.61 (d, *J* = 8.3 Hz, 2H), 7.33-7.23 (m, 6H), 4.57 (dd, *J* = 10.2, 2.6 Hz, 1H), 4.09-4.02 (m, 1H), 3.83 (dt, *J* = 2.7, 11.6 Hz, 1H), 3.77-3.69 (m, 1H), 3.62 (d, *J* = 10.9 Hz, 1H), 2.49 (dt, *J* = 3.4, 11.6 Hz, 1H), 2.43 (s, 3H), 2.18 (dd, *J* = 10.4, 11.4 Hz, 1H). ^{13}C NMR (100 MHz, CDCl3): δ 144.0, 137.1, 134.0, 132.1, 129.8, 128.6, 127.8, 127.3, 76.6, 66.1, 51.8, 45.3, 21.5. HRMS-ESI (m/z): $[M+H]^+$ calculated for $C_{17}H_{19}ClNO_3S$, 352.0774; found: 352.0779.

2-(2-Chlorophenyl)-4-tosylmorpholine (3g). To a 10 mL round bottomed flask was added **1g** (92 mg, 0.3 mmol), $(NH_4)_2S_2O_8$ (137 mg, 0.6 mmol) and chloroethanol (240 mg, 3.0 mmol) at rt for 0.5 h. THF (5.0 mL) and KOH (202 mg, 3.6 mmol) were then added and stirred at rt for further 1 h to afford **3g** as a colorless liquid (92 mg, 87% yield). ^{1}H NMR (400 MHz, $CDCl_3$): δ 7.63 (d, *J* = 8.2 Hz, 2H), 7.42-7.40 (m, 1H), 7.36-7.31 (m, 3H), 7.25-7.22 (m, 2H), 4.94 (dd, *J* = 2.4, 10.1 Hz, 1H), 4.08 (dd, *J* = 2.4, 11.5 Hz, 1H), 3.96 (d, *J* = 11.6 Hz, 1H), 3.87 (dt, *J* = 2.6, 11.7 Hz, 1H), 3.66 (d, *J* = 11.2 Hz, 1H), 2.54 (dt, *J* = 3.4, 11.7 Hz, 1H), 2.43 (s, 3H), 2.11 (t, *J* = 10.3, 11.4 Hz, 1H). ^{13}C NMR (100 MHz, $CDCl_3$): δ 143.9, 136.3, 132.6, 131.9, 129.8, 129.4, 129.2, 127.8, 127.4, 127.0, 74.4, 66.3, 50.3, 45.4, 21.5. HRMS-ESI (*m*/*z*): $[M+H]^+$ calculated for $C_{17}H_{19}ClNO_3S$, 352.0774; found: 352.0779.

4-Tosyl-2-(4-(trifluoromethyl)phenyl)morpholine (3h). To a 10 mL rounded bottom flask was added **1h** (102 mg, 0.3 mmol), $(NH_4)_2S_2O_8$ (137 mg, 0.6 mmol) and chloroethanol (240 mg, 3.0 mmol) at rt for 0.5 h. THF (5.0 mL) and KOH (202 mg, 3.6 mmol) were then added and stirred at rt for further 1 h to afford **3h** as a white solid (90 mg, 78% yield). ^{1}H NMR (400 MHz, CDCl3): δ 7.62-7.59 (m, 4H), 7.44 (d, *J* = 8.2 Hz, 2H), 7.33 (d, *J* = 8.0 Hz, 2H), 4.66 (dd, *J* = 10.2, 2.3 Hz, 1H), 4.09 (dd, *J* = 11.7, 2.1 Hz, 1H), 3.86 (dt, *J* = 2.7, 11.6 Hz, 1H), 3.78 (td, *J* = 2.0, 11.5 Hz, 1H), 3.65 (d, *J* = 11.7 Hz, 1H), 2.51 (dt, *J* = 3.4, 11.6 Hz, 1H), 2.43 (s, 3H), 2.19 (dd, *J* = 11.4, 10.5 Hz, 1H). ^{13}C NMR (100 MHz, $CDCl_3$): δ 144.1, 142.6 (d, *J* = 1.0 Hz), 132.1, 130.4 (q, *J* = 32.6 Hz), 129.8, 127.8, 126.3, 125.4 (q, *J* = 3.8 Hz), 123.9 (q, *J* =

270.0 Hz), 76.6, 66.2, 51.8, 45.3, 21.5. ^{19}F NMR (376MHz, $CDCl_3$): δ -62.6 (S). HRMS-ESI (m/z): $[M+H]^+$ calculated for $C_{18}H_{19}F_3NO_3S$, 386.1038; found: 386.1038.

2-(4-Nitrophenyl)-4-tosylmorpholine (3i). To a 10 mL round bottom flask was added **1i** (96 mg, 0.3 mmol), $(NH_4)_2S_2O_8$ (137 mg, 0.6 mmol) and chloroethanol (240 mg, 3.0 mmol) at rt for 3 h. THF (5.0 mL) and KOH (202 mg, 3.6 mmol) were then added and stirred at rt for further 1 h to afford **3i** as a white solid (90 mg, 83% yield). ^{1}H NMR (400 MHz, $CDCl_3$): δ 8.20 (d, *J* = 8.7 Hz, 2H), 7.62 (d, *J* = 8.2 Hz, 2H), 7.50 (d, *J* = 8.7 Hz, 2H), 7.33 (d, *J* = 8.1 Hz, 2H), 4.71 (dd, *J* = 2.5 Hz, 10.2 Hz, 1H), 4.10 (dd, *J* = 2.1, 11.7 Hz, 1H), 3.87 (dt, *J* = 2.7, 11.6 Hz, 1H), 3.80 (td, *J* = 1.9, 11.6 Hz, 1H), 3.66 (d, *J* = 11.1 Hz, 1H), 2.52 (dt, *J* = 3.4, 11.6 Hz, 1H), 2.43 (s, 3H), 2.19 (dd, *J* = 10.5, 11.3 Hz, 1H). ^{13}C NMR (100 MHz, $CDCl_3$): δ 147.7, 145.6, 144.2, 132.0, 129.9, 127.8, 126.8, 123.7, 76.3, 66.2, 51.5, 45.3, 21.5. HRMS-ESI (*m*/*z*): $[M+H]^+$ calculated for $C_{17}H_{19}N_2O_5S$, 363.1015; found: 363.1016.

2-(Naphthalen-1-yl)-4-tosylmorpholine (3j). To a 10 mL round bottomed flask was added **1j** (97 mg, 0.3 mmol), $(NH_4)_2S_2O_8$ (137 mg, 0.6 mmol) and chloroethanol (240 mg, 3.0 mmol) at rt for 1 h. THF (5.0 mL) and KOH (202 mg, 3.6 mmol) were then added and stirred at rt for further 1 h to afford **3j** as a white solid (90 mg, 82% yield). ^{1}H NMR (400 MHz, $CDCl_3$): δ 8.01 (d, *J* = 8.4 Hz, 1H), 7.86 (d, *J* = 8.0 Hz, 1H), 7.78 (d, *J* = 8.1 Hz, 1H), 7.59-7.47 (m, 5H), 7.42 (t, *J* = 7.7 Hz, 1H), 7.26 (d, *J* = 8.1 Hz, 2H), 5.32 (dd, *J* = 1.9, 10.0 Hz, 1H), 4.16 (dd, *J* = 2.3, 11.7 Hz, 1H), 4.02-3.96 (m, 2H), 3.71 (d, *J* = 11.3 Hz, 1H), 2.60 (dt, *J* = 3.4, 11.6 Hz, 1H), 2.39-2.32 (m, 4H). ^{13}C NMR (100 MHz, $CDCl_3$): δ 143.8, 134.3, 133.5, 132.2, 129.9, 129.696, 128.9, 128.6, 127.7, 126.6, 125.7, 125.3, 123.6, 122.5, 74.5, 66.5, 51.6, 45.6, 21.4. HRMS-ESI (*m*/*z*): $[M+H]^+$ calculated for $C_{21}H_{22}NO_3S$, 368.1320; found: 368.1322.

4-Tosyloctahydro-2*H*-benzo[*b*][1,4]oxazine (3k). To a 10 mL round bottome flask was added **1k** (75 mg, 0.3 mmol), $(NH_4)_2S_2O_8$ (137 mg, 0.6 mmol) and chloroethanol (240 mg, 3.0 mmol) at rt for 5 h. THF (5.0 mL) and KOH (202 mg, 3.6 mmol) were then added and stirred at rt for further 3 h to afford **3k** as a white solid (84 mg, 95% yield). ^{1}H NMR (400 MHz, $CDCl_3$): δ 7.65 (d, *J* = 8.3Hz, 2H), 7.34 (d, *J* = 8.1 Hz, 2H), 3.92-3.86 (m, 2H), 3.77 (dt, *J* = 2.2, 11.2 Hz, 1H), 3.25-3.19 (m, 1H), 2.76 (dt, *J* = 3.7, 12.1 Hz, 1H), 2.57-2.52 (m, 1H), 2.45 (s, 3H), 2.28 (ddd, *J* = 3.8, 8.8, 11.9 Hz,

1H), 1.89-1.85 (m, 1H), 1.75-1.65 (m, 2H), 1.52-1.42 (m, 1H), 1.32-1.09 (m, 3H). ^{13}C NMR (100 MHz, $CDCl_3$): δ 143.6, 134.6, 129.7, 127.5, 79.5, 66.4, 63.4, 48.9, 31.5, 29.5, 24.8, 23.9, 21.5. HRMS-ESI(*m*/*z*): $[M+H]^+$ calculated for $C_{15}H_{22}NO_3S$, 296.1320; found, 296.1336.

***cis*-3-Methyl-2-phenyl-4-tosylmorpholine (3l).** To a 10 mL round bottomed flask was added **1l** (86 mg, 0.3 mmol), $(NH_4)_2S_2O_8$ (137 mg, 0.6 mmol) and chloroethanol (240 mg, 3.0 mmol) at rt for 0.5 h. THF (5.0 mL) and KOH (202 mg, 3.6 mmol) were then added and stirred at rt for further 1 h to afford **3l** and **4l**. Regioisomer **3l** (75 mg, 75% yield) colorless liquid: ^{1}H NMR (400 MHz, $CDCl_3$): δ 7.73 (d, *J* = 7.9 Hz, 2H), 7.34-7.24 (m, 7H), 4.62 (d, *J* = 1.4 Hz, 1H), 4.20 (qd, *J* = 6.4, 2.2 Hz, 1H), 4.04 (dd, *J* = 11.4, 3.2 Hz, 1H), 3.69 (td, *J* = 11.9, 2.8 Hz, 1H), 3.60 (d, *J* = 13.1 Hz, 1H), 3.25 (td, *J* = 12.6, 3.4 Hz, 1H), 2.42 (s, 3H), 0.73 (d, *J* = 6.8 Hz, 3H). ^{13}C NMR (100 MHz, $CDCl_3$): δ 143.4, 138.6, 137.5, 129.8, 128.3, 127.4, 127.1, 125.3, 80.2, 67.0, 53.2, 39.4, 21.5, 9.0. HRMS-ESI(*m*/*z*): $[M+H]^+$ calculated for $C_{18}H_{22}NO_3S$, 332.1320; found: 332.1322.

***cis*-2-Methyl-3-phenyl-4-tosylmorpholine (4l).** 16 mg, 16% yield, white solid. ^{1}H NMR (400 MHz, $CDCl_3$): δ 7.68 (d, *J* = 7.9 Hz, 2H), 7.42 (d, *J* = 7.5 Hz, 2H), 7.37-7.30 (m, 5H), 4.39 (d, *J* = 4.4 Hz, 1H), 3.97-3.91 (m, 1H), 3.79-3.73 (m, 1H), 3.71-3.66 (m, 1H), 3.58-3.52 (m, 1H), 3.19 (dt, *J* = 12.1, 4.1 Hz, 1H), 2.44 (s, 3H), 1.21 (d, *J* = 6.6 Hz, 3H). ^{13}C NMR (100 MHz, $CDCl_3$): δ 143.6, 138.2, 136.1, 129.8, 128.5, 128.1, 127.8, 127.5, 80.3, 62.6, 52.9, 43.0, 21.5, 15.4. HRMS-ESI(*m*/*z*): $[M+H]^+$ calculated for $C_{18}H_{22}NO_3S$, 332.1320; found: 332.1322.

4-Tosyl-2,3,4,4a,5,9b-hexahydroindeno[1,2-*b*][1,4]oxazine (3m). To a 10 mL round bottomed flask was added **1m** (86 mg, 0.3 mmol), $(NH_4)_2S_2O_8$ (137 mg, 0.6 mmol) and chloroethanol (240 mg, 3.0 mmol) at rt for 0.5 h. THF (5.0 mL) and KOH (202 mg, 3.6 mmol) were then added and stirred at rt for further 5 h to afford **3m** and **4m**. Regioisomer **3m** (40 mg, 40% yield) white solid: ^{1}H NMR (400 MHz, $CDCl_3$): δ 7.71 (d, *J* = 8.3 Hz, 2H), 7.37 (d, *J* = 8.1 Hz, 2H), 7.29-7.26 (m, 4H), 4.64 (d, *J* = 9.1 Hz, 1H), 4.15 (ddd, *J* = 11.6, 3.7, 1.0 Hz, 1H), 4.05 (td, *J* = 11.8, 2.8 Hz, 1H), 3.74-3.71 (m, 1H), 3.35 (dd, *J* = 15.1, 6.7 Hz, 1H), 3.26 (dd, *J* = 15.1, 10.7 Hz, 1H), 2.58-2.51 (m, 2H), 2.45 (s, 3H); ^{13}C NMR (100 MHz, $CDCl_3$): δ 143.7, 141.8, 139.6, 136.8, 129.9, 129.5, 127.3, 127.2, 125.3, 125.2, 77.9, 64.3, 55.2, 40.3, 30.6, 21.5;

HRMS-ESI (*m*/*z*): $[M+H]^+$ calculated for $C_{18}H_{20}NO_3S$, 330.1164; found: 330.1167.

4-Tosyl-2,3,4,4a,9,9a-hexahydroindeno[2,1-*b*][1,4]oxazine (4m). 47 mg, 47% yield, white solid. ^{1}H NMR (400 MHz, $CDCl_3$): δ 7.76 (d, J = 8.3 Hz, 2H), 7.42-7.16 (m, 6H), 4.60 (d, J = 4.5 Hz, 1H), 4.39-4.28 (m, 1H), 3.82 (dd, J = 11.5, 1.9 Hz, 1H), 3.68 (d, J = 12.8 Hz, 1H), 3.58 (td, J = 11.5, 2.4 Hz, 1H), 3.11 (td, J = 12.4, 3.1 Hz, 1H), 2.91 (dd, J = 15.1, 9.6 Hz, 1H), 2.78 (dd, J = 15.2, 7.4 Hz, 1H), 2.46 (s, 3H); ^{13}C NMR (100 MHz, $CDCl_3$): δ 143.7, 141.8, 139.6, 136.8, 129.9, 129.5, 127.3, 127.2, 125.3, 125.2, 77.9, 64.3, 55.2, 40.3, 30.6, 21.5; HRMS-ESI (*m*/*z*): $[M+H]^+$ calculated for $C_{18}H_{20}NO_3S$, 330.1164; found: 330.1167.

2-Butyl-4-tosylmorpholine (3n). To a 10 mL round bottomed flask was added **1n** (76 mg, 0.3 mmol), $(NH_4)_2S_2O_8$ (137 mg, 0.6 mmol) and chloroethanol (240 mg, 3.0 mmol) at rt for 5 h. THF (5.0 mL) and KOH (202 mg, 3.6 mmol) were then added and stirred at rt for further 3 h to afford **3n** and **4n**. Regioisomer **3n** (40 mg, 45% yield) colorless liquid: ^{1}H NMR (400 MHz, $CDCl_3$): δ 7.63 (d, J = 8.1 Hz, 2H), 7.34 (d, J = 8.1 Hz, 2H), 3.88 (dd, J = 2.1, 11.6 Hz, 1H), 3.65 (dt, J = 2.5, 11.5 Hz, 1H), 3.56-3.46 (m, 3H), 2.44 (s, 3H), 2.38 (dt, J = 3.3, 11.4 Hz, 1H), 2.03 (t, J = 10.5 Hz, 1H), 1.45-1.26 (m, 6H), 0.89 (t, J = 6.7 Hz, 3H). ^{13}C NMR (100 MHz, $CDCl_3$): δ 143.8, 132.2, 129.7, 127.8, 75.3, 65.9, 50.4, 45.5, 32.8, 27.2, 22.540, 21.5, 13.8. HRMS-ESI (*m*/*z*): $[M+H]^+$ calculated for $C_{15}H_{24}NO_3S$, 298.1477; found: 298.1483.

3-Butyl-4-tosylmorpholine (4n). 27 mg, 30% yield, colorless liquid. ^{1}H NMR (400 MHz, $CDCl_3$): δ 7.70 (d, J = 8.3 Hz, 2H), 7.30 (d, J = 8.0 Hz, 2H), 3.75-3.69 (m, 2H), 3.66 (d, J = 11.6 Hz, 1H), 3.54 (d, J = 11.7 Hz, 1H), 3.41 (dd, J = 2.9, 11.5 Hz, 1H), 3.35-3.22 (m, 2H), 2.43 (s, 3H), 1.74-1.63 (m, 1H), 1.59-1.50 (m, 1H), 1.33-1.20 (m, 4H), 0.86 (t, J = 6.9 Hz, 3H). ^{13}C NMR (100 MHz, $CDCl_3$): δ 143.3, 138.2, 129.7, 127.0, 68.4, 66.1, 53.5, 40.7, 28.4, 27.6, 22.4, 21.5, 13.9. HRMS-ESI (*m*/*z*): $[M+H]^+$ calculated for $C_{15}H_{24}NO_3S$, 298.1477; found: 298.1486.

(*R*)-2-Phenyl-4-tosylmorpholine (3a). Enantiomeric purity was determined by chiral HPLC analysis (Chiralcel OD-H column, hexane/isopropanol, 90:10, flow rate = 1.0 mL/min; t_R1: 8.43 (minor), t_R2: 15.71 (major).

(*R*)-2-Phenyl-4-tosyl-1,4-oxazepane (3ab). To a 10 mL round bottomed flask was added (*S*)-**1a** (82 mg, 0.3 mmol), $(NH_4)_2S_2O_8$ (137 mg, 0.6 mmol) and 3-bromopropyl alcohol (417 mg, 3.0 mmol) at rt for 0.5 h. THF (5.0 mL) and KOH (202 mg,

3.6 mmol) were then added and stirred at rt for further 16 h to afford **3ab** as a colorless liquid (72 mg, 72% yield). Enantiomeric purity was determined by chiral HPLC analysis (Chiralcel OD-H column, hexane-isopropanol, 98:2, flow rate = 1.0 mL/min; t_R1: 15.87 (minor), t_R2: 17.32 (major). ^{1}H NMR (400 MHz, $CDCl_3$): δ 7.66 (d, *J* = 8.3 Hz, 2H), 7.34-7.26 (m, 7H), 4.63 (dd, *J* = 10.0, 2.5 Hz, 1H), 4.21-4.14 (m, 1H), 3.97 (ddd, *J* = 14.0, 2.3, 1.4 Hz, 1H), 3.91-3.84 (m, 2H), 3.04 (ddd, *J* = 13.8, 8.1, 5.9 Hz, 1H), 2.82 (dd, *J* = 14.0, 10.0 Hz, 1H), 2.41 (s, 3H), 2.14-2.06 (m, 2H). ^{13}C NMR (100 MHz, $CDCl_3$): δ 143.30, 139.63, 136.17, 129.72, 128.45, 127.89, 126.86, 125.97, 83.95, 68.56, 57.71, 46.86, 30.51, 21.45. HRMS-ESI (*m*/*z*): $[M+H]^+$ calculated for $C_{18}H_{22}NO_3S$, 332.1320; found: 332.1326.

(*S*)-7-Phenyl-4-tosyl-1,4-oxazepane (3o). To a 10 mL round bottomed flask was added (*R*)-**1o** (86 mg, 0.3 mmol), $(NH_4)_2S_2O_8$ (137 mg, 0.6 mmol) and 2-bromoethanol (375 mg, 3.0 mmol) at rt for 1 h. THF (5.0 mL) and KOH (202 mg, 3.6 mmol) were then added and stirred at rt for further 36 h to afford **3o** as a white solid (65 mg, 65% yield). Enantiomeric purity was determined by chiral HPLC analysis (Chiralcel OD-H column, hexane/isopropanol, 95:5, flow rate = 1.0 mL/min; t_R1: 16.82 (minor), t_R2: 19.54 (major). ^{1}H NMR (400 MHz, $CDCl_3$): δ 7.71 (d, *J* = 7.9 Hz, 2H), 7.34-7.23 (m, 7H), 4.67 (dd, *J* = 9.6, 4.1 Hz, 1H), 4.10 (d, *J* = 12.9 Hz, 1H), 3.80-3.59 (m, 3H), 3.30 (ddd, *J* = 33.9, 16.9, 7.3 Hz, 2H), 2.44 (s, 3H), 2.34-2.28 (m, 1H), 2.05-2.02 (m, 1H). ^{13}C NMR (100 MHz, $CDCl_3$): δ 143.4, 142.9, 135.9, 129.8, 128.4, 127.4, 127.1, 125.5, 81.5, 70.0, 51.7, 46.2, 37.8, 21.5. HRMS-ESI (*m*/*z*): $[M+H]^+$ calculated for $C_{18}H_{22}NO_3S$, 332.1320; found: 332.1320.

(*S*)-2-Phenyl-5-tosyl-1,5-oxazocane (3ob). To a 10 mL round bottomed flask was added (*R*)-**1o** (86 mg, 0.3 mmol), $(NH_4)_2S_2O_8$ (137 mg, 0.6 mmol) and 3-bromopropyl alcohol (417 mg, 3.0 mmol) at rt for 1 h. THF (5.0 mL) and KOH (202 mg, 3.6 mmol) were then added and stirred at rt for further 48 h to afford **3ob** as a colorless liquid (62 mg, 60% yield). Enantiomeric purity was determined by chiral HPLC analysis (Chiralcel OD-H column, hexane-isopropanol, 95:5, flow rate = 1.0 mL/min; t_R1: 9.11 (minor), t_R2: 10.50 (major). ^{1}H NMR (400 MHz, $CDCl_3$): δ 7.73 (d, *J* = 7.8 Hz, 2H), 7.34-7.26 (m, 7H), 4.73 (dd, *J* = 10.0, 3.9 Hz, 1H), 3.92-3.86 (m, 1H), 3.78-3.70 (m, 2H), 3.58 (dt, *J* = 14.3, 3.9 Hz, 1H), 3.21 (ddd, *J* = 14.2, 10.3, 3.6 Hz, 1H), 3.03 (dd, *J* = 13.7, 8.6 Hz, 1H), 2.43 (s, 3H), 2.34-2.20 (m, 2H), 1.92-1.83 (m, 2H). ^{13}C NMR (100 MHz, $CDCl_3$): δ 143.1, 143.0, 136.7, 129.7, 128.3, 127.1, 126.9, 125.7, 76.41,

66.4, 49.0, 47.6, 37.4, 30.4, 21.5. HRMS-ESI (*m/z*): $[M+H]^+$ calculated for $C_{19}H_{24}NO_3S$ 346.1477; found: 346.1478.

(*R*)-2-Isobutyl-4-tosylmorpholine (3p). To a 10 mL round bottomed flask was added (*S*)-**1p** (76 mg, 0.3 mmol), $(NH_4)_2S_2O_8$ (137 mg, 0.6 mmol) and chloroethanol (240 mg, 3.0 mmol) at rt for 12 h. THF (5.0 mL) and KOH (202 mg, 3.6 mmol) were then added and stirred at rt for further 3 h to afford **3p** and **4p**. Regioisomer **3p** (40 mg, 45% yield) colorless liquid. Enantiomeric purity was determined by chiral HPLC analysis (Chiralpak AS-H column, hexane/isopropanol, 98:2, flow rate = 0.8 mL/min; t_R1: 25.77 (major), t_R2: 32.34 (minor). ^1H NMR (400 MHz, $CDCl_3$): δ 7.63 (d, *J* = 8.2 Hz, 2H), 7.34 (d, *J* = 8.1 Hz, 2H), 3.88 (dd, *J* = 2.2, 11.4 Hz, 1H), 3.65 (dt, *J* = 2.4, 11.5 Hz, 1H), 3.59-3.50 (m, 3H), 2.44 (s, 3H), 2.37 (dt, *J* = 3.3, 11.5 Hz, 1H), 2.02 (t, *J* = 10.8 Hz, 1H), 1.80-1.69 (m, 1H), 1.36 (ddd, *J* = 5.9, 8.4, 14.0 Hz, 1H), 1.13 (ddd, *J* = 4.3, 8.3, 13.1 Hz, 1H), 0.90 (d, *J* = 2.3 Hz, 3H), 0.88 (t, *J* = 2.2 Hz, 3H). ^{13}C NMR (100 MHz, $CDCl_3$): δ 143.8, 132.2, 129.7, 127.8, 73.6, 65.8, 50.6, 45.5, 42.1, 24.1, 23.1, 22.1, 21.5. HRMS-ESI(*m/z*): $[M+H]^+$ calculated for $C_{15}H_{24}NO_3S$, 298.1477; found, 298.1481.

(*S*)-3-Isobutyl-4-tosylmorpholine (4p). 14 mg, 16% yield, white solid. Enantiomeric purity was determined by chiral HPLC analysis (Chiralpak AS-H column, hexane/isopropanol, 98:2, flow rate = 0.8 mL/min; t_R1: 31.28 (major), t_R2: 38.92 (minor). ^1H NMR (400 MHz, $CDCl_3$): δ 7.71 (d, *J* = 8.2 Hz, 2H), 7.31 (d, *J* = 8.1 Hz, 2H), 3.84-3.83 (m, 1H), 3.72-3.69 (m, 1H), 3.62 (d, *J* = 11.5 Hz, 1H), 3.54 (d, *J* = 12.4 Hz, 1H), 3.44 (dd, *J* = 2.9, 11.5 Hz, 1H), 3.36-3.22 (m, 2H), 2.43 (s, 3H), 1.59-1.51 (m, 2H), 1.44-1.36 (m, 1H), 0.89 (d, *J* = 6.2 Hz, 6H). ^{13}C NMR (100 MHz, $CDCl_3$): δ 143.3, 138.1, 129.8, 127.1, 68.8, 66.1, 51.7, 40.7, 36.9, 24.9, 22.7, 22.4, 21.5. HRMS-ESI(*m/z*): $[M+H]^+$ calculated for $C_{15}H_{24}NO_3S$, 298.1477; found, 298.1482.

(*R*)-2-((*S*)-sec-Butyl)-4-tosylmorpholine (3q). To a 10 mL round bottomed flask was added (*S*)-**1q** (76 mg, 0.3 mmol), $(NH_4)_2S_2O_8$ (137 mg, 0.6 mmol) and chloroethanol (240 mg, 3.0 mmol) at rt for 12 h. THF (5.0 mL) and KOH (202 mg, 3.6 mmol) were then added and stirred at rt for further 3 h to afford **3q** and **4q**. Regioisomer **3q** (34 mg, 38% yield) colorless liquid. Enantiomeric purity was determined by chiral HPLC analysis (Chiralpak AS-H column, hexane/isopropanol, 98:2, flow rate =

0.8 mL/min; t_R1: 27.51 (major), t_R2: 32.84 (minor). ^{1}H NMR (400 MHz, $CDCl_3$): δ 7.64 (d, J = 8.2 Hz, 2H), 7.34 (d, J = 8.1 Hz, 2H), 3.90 (dd, J = 2.2, 11.5 Hz, 1H), 3.63 (dt, J = 2.6, 11.5 Hz, 1H), 3.58-3.50 (m, 2H), 3.37-3.32 (m, 1H), 2.45 (s, 3H), 2.36 (dt, J = 3.4, 11.4 Hz, 1H), 2.14 (t, J = 10.7 Hz, 1H), 1.49-1.39 (m, 1H), 1.18-1.09 (m, 1H), 0.90-0.87 (m, 6H). ^{13}C NMR (100 MHz, $CDCl_3$): δ 143.8, 132.4, 129.7, 127.8, 78.7, 66.0, 48.3, 45.6, 37.6, 25.2, 21.5, 14.5, 11.4. HRMS-ESI(*m*/*z*): $[M+H]^+$ calculated for $C_{15}H_{24}NO_3S$, 298.1477; found, 298.1483.

(*S*)-3-((*S*)-sec-Butyl)-4-tosylmorpholine (4q). 19 mg, 21% yield, white solid. Enantiomeric purity was determined by chiral HPLC analysis (Chiralpak AS-H column, hexane-isopropanol, 98:2, flow rate = 0.8 mL/min; t_R1: 34.80 (major), t_R2: 44.87 (minor). ^{1}H NMR (400 MHz, $CDCl_3$): δ 7.72 (d, J = 8.3 Hz, 2H), 7.31 (d, J = 8.1 Hz, 2H), 3.82 (d, J = 12.0 Hz, 1H), 3.62-3.56 (m, 2H), 3.41 (dd, J = 2.6, 10.4 Hz, 1H), 3.34-3.26 (m, 1H), 3.22-3.12 (m, 2H), 2.43 (s, 3H), 2.09-1.98 (m, 2H), 1.69-1.60 (m, 1H), 1.14-1.03 (m, 1H), 0.94-0.88 (m, 6H). ^{13}C NMR (100 MHz, $CDCl_3$): δ 143.3, 138.8, 129.8, 127.0, 66.3, 65.4, 58.4, 41.3, 31.7, 25.3, 21.5, 15.9, 11.2. HRMS-ESI(*m*/*z*): $[M+H]^+$ calculated for $C_{15}H_{24}NO_3S$, 298.1477; found: 298.1484.

4. Copies of HPLC chromatograms for ee determination

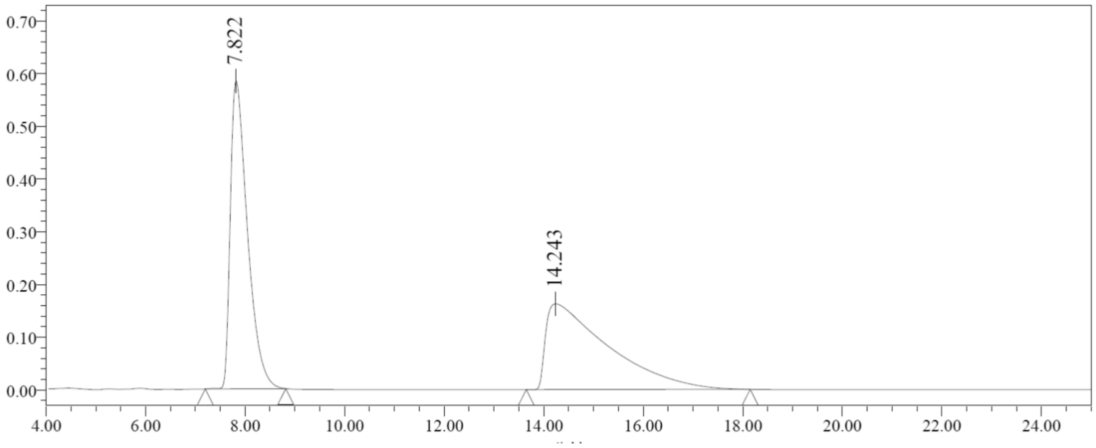

Figure S1 Chromatogram of racemic **3a**

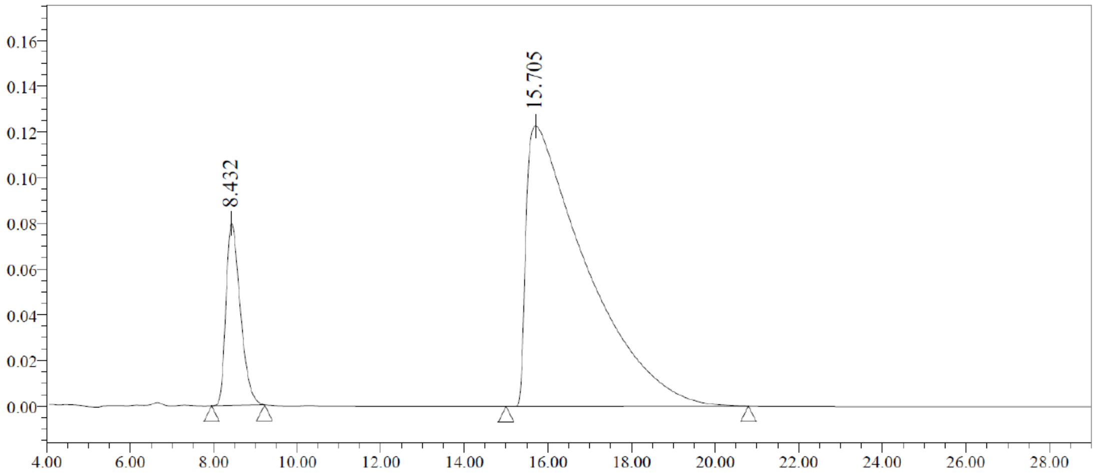

Figure S2 Chromarogram of **3a** (70% *ee*)

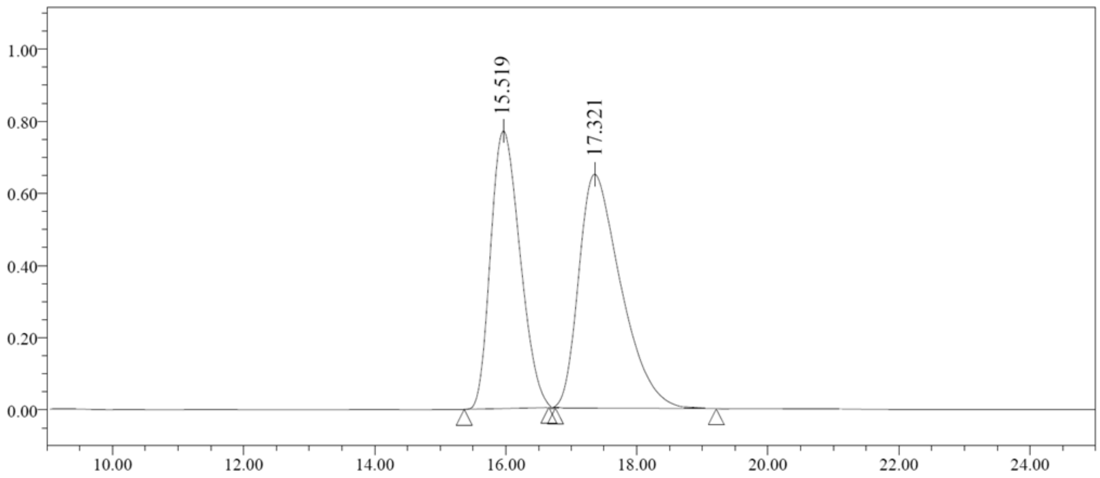

Figure S3 Chromatogram of racemic **3ab**

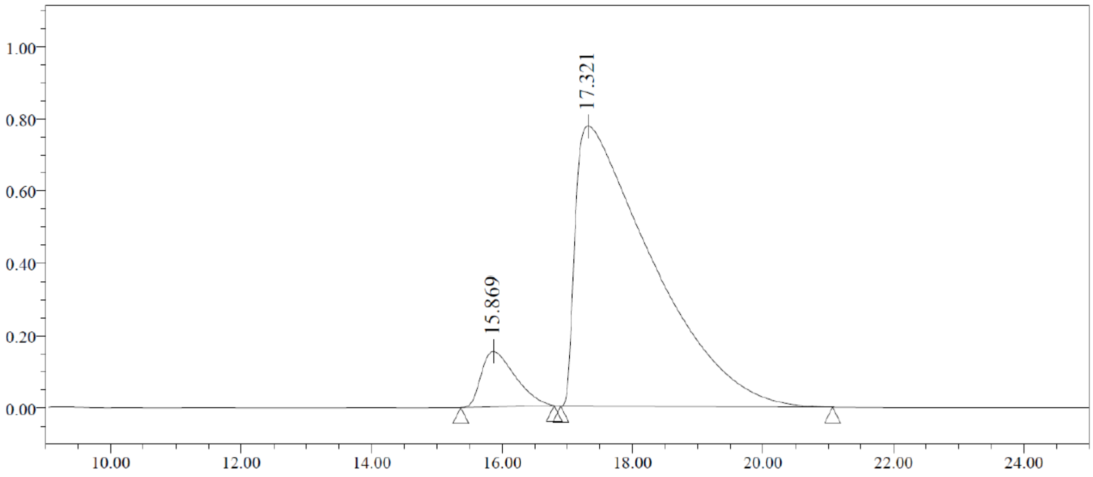

Figure S4 Chromatogram of **3ab** (84% *ee*)

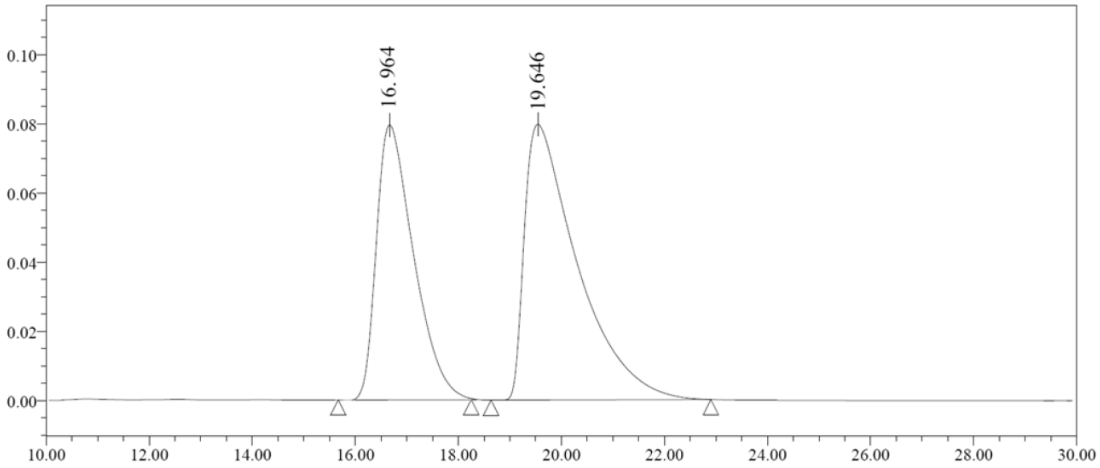

Figure S5 Chromatogram of racemic **3o**

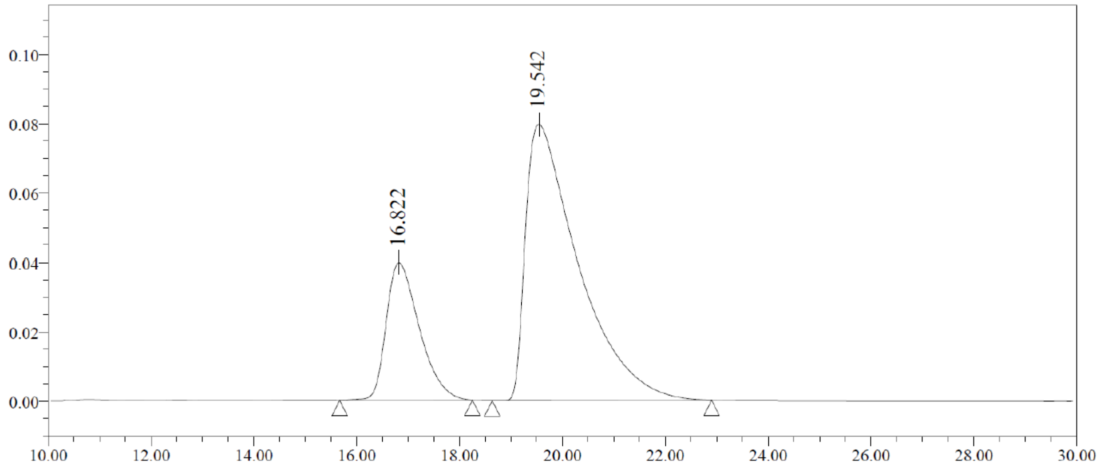

Figure S6 Chromatogram of **3o** (52% *ee*)

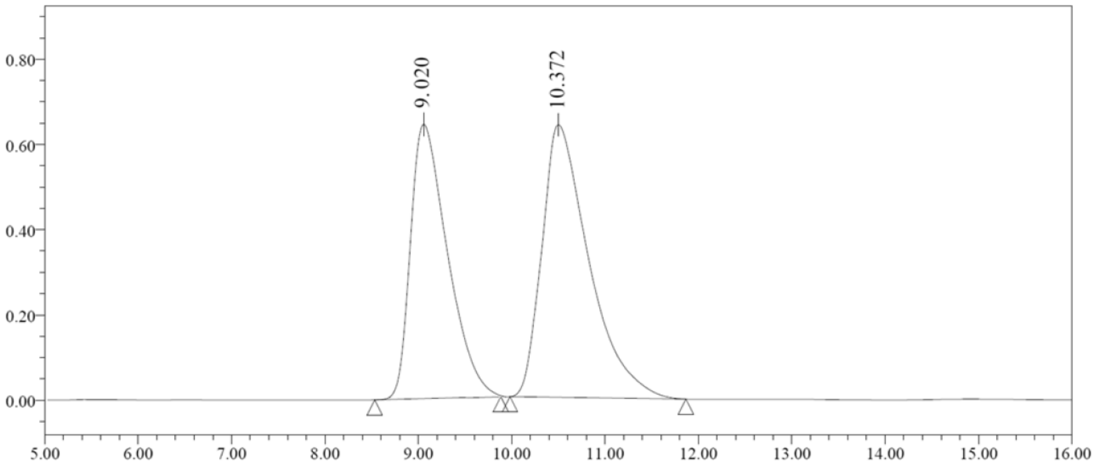

Figure S7 Chromatogram of racemic **3ob**

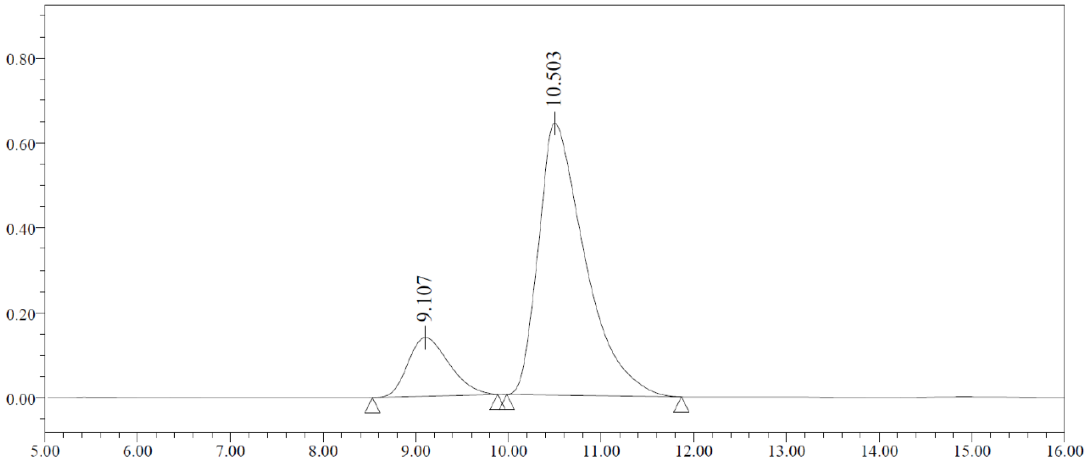

Figure S8 Chromatogram of **3ob** (67% *ee*)

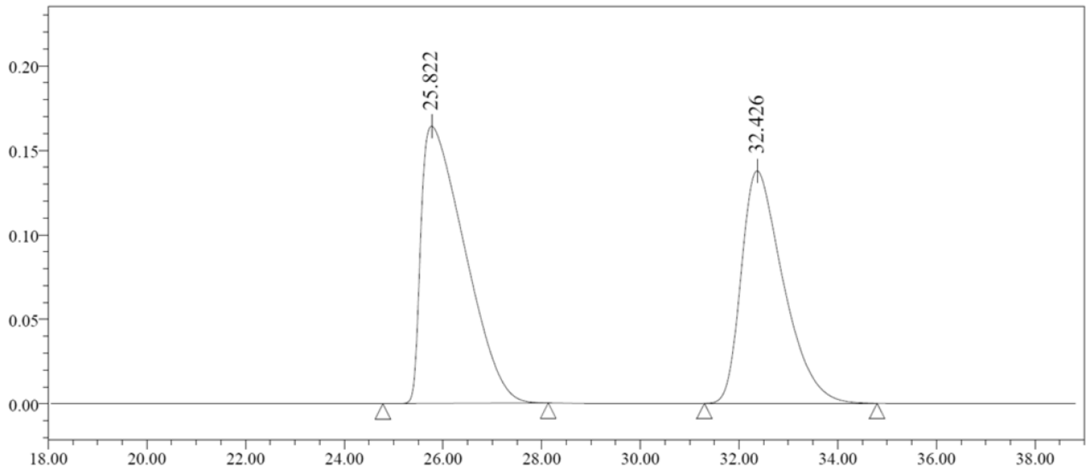

Figure S9 Chromatogram of racemic **3p**

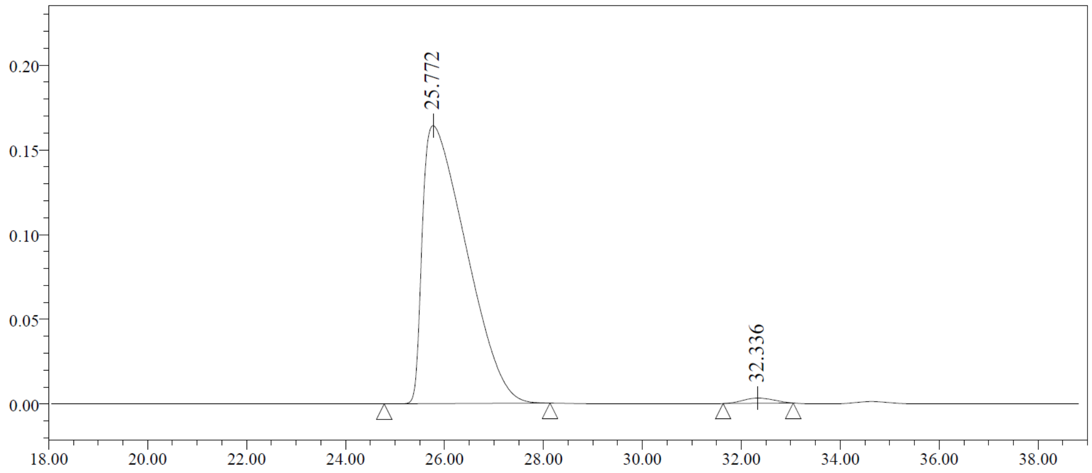

Figure S10 Chromatogram of **3p** (97% *ee*)

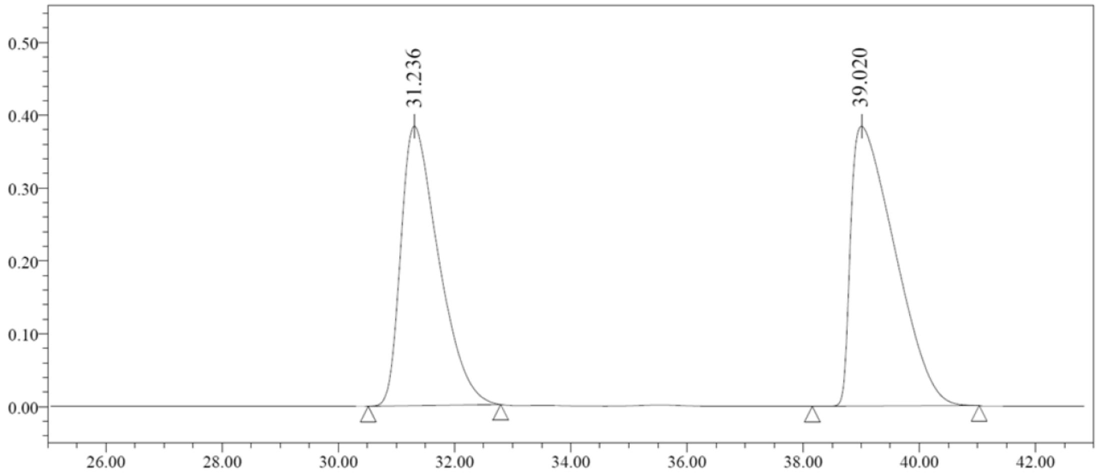

Figure S11 Chromatogram of racemic **4p**

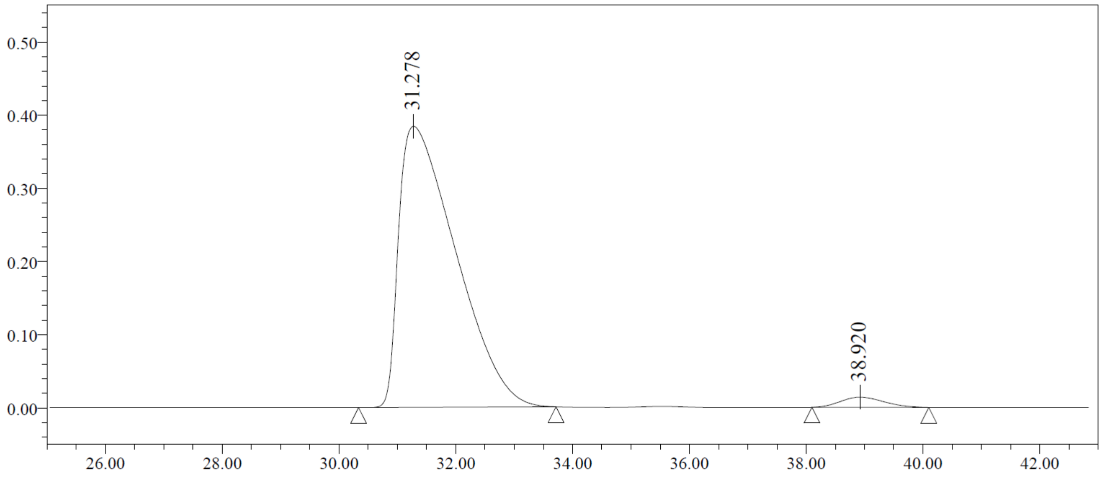

Figure S12 Chromatogram of **4p** (95% *ee*)

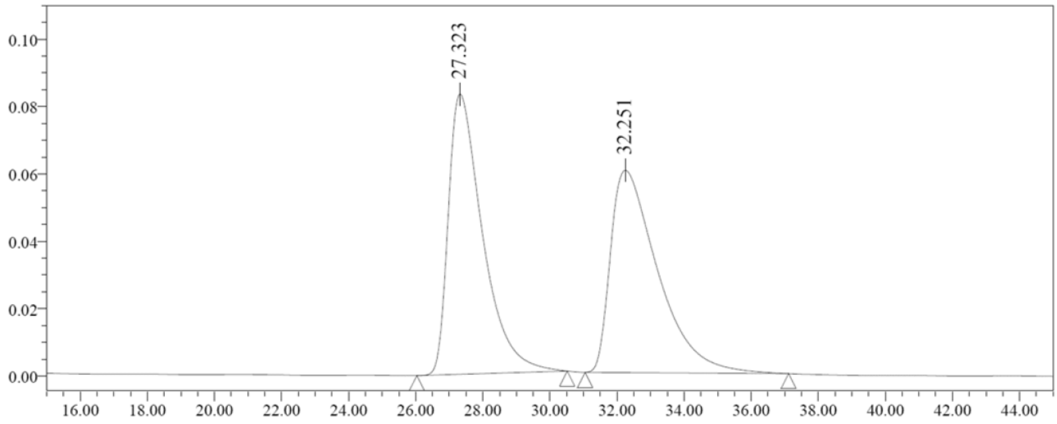

Figure S13 Chromatogram of racemic **3q**

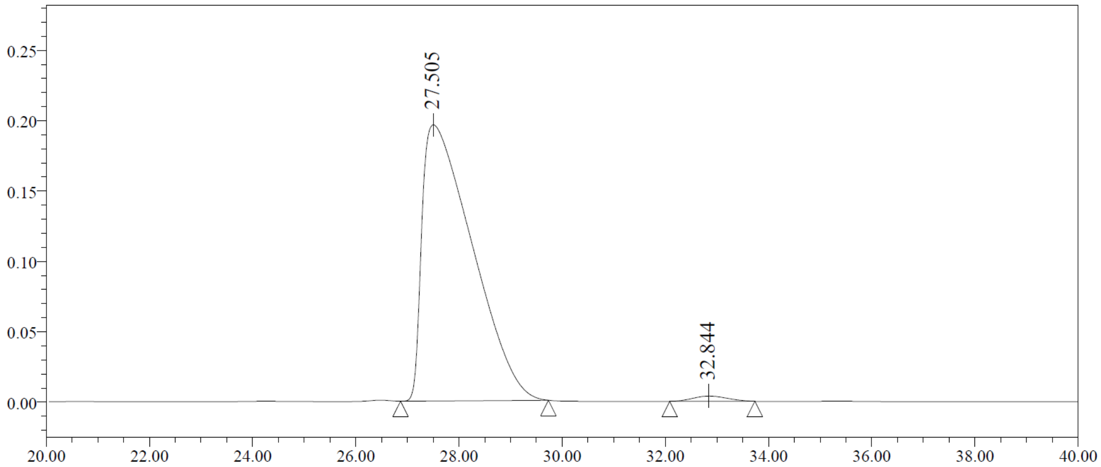

Figure S14 Chromatogram of **3q** (97% *ee*)

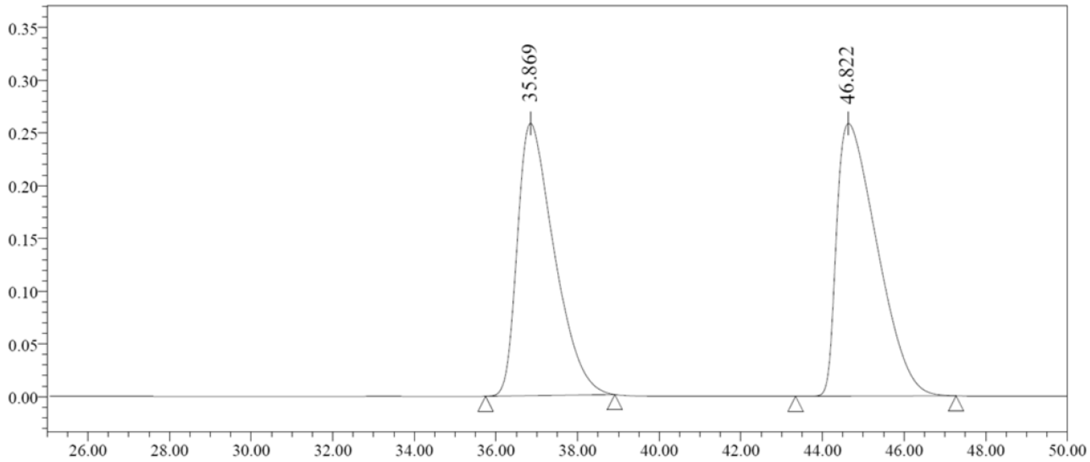

Figure S15 Chromatogram of racemic **4q**

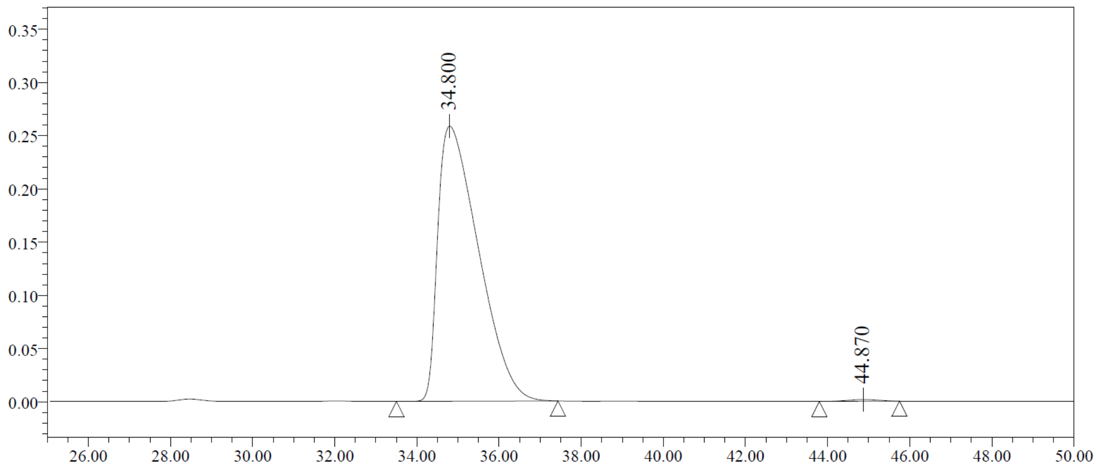

Figure S16 Chromatogram of **4q** (99% *ee*)

5. Copies of NMR spectra

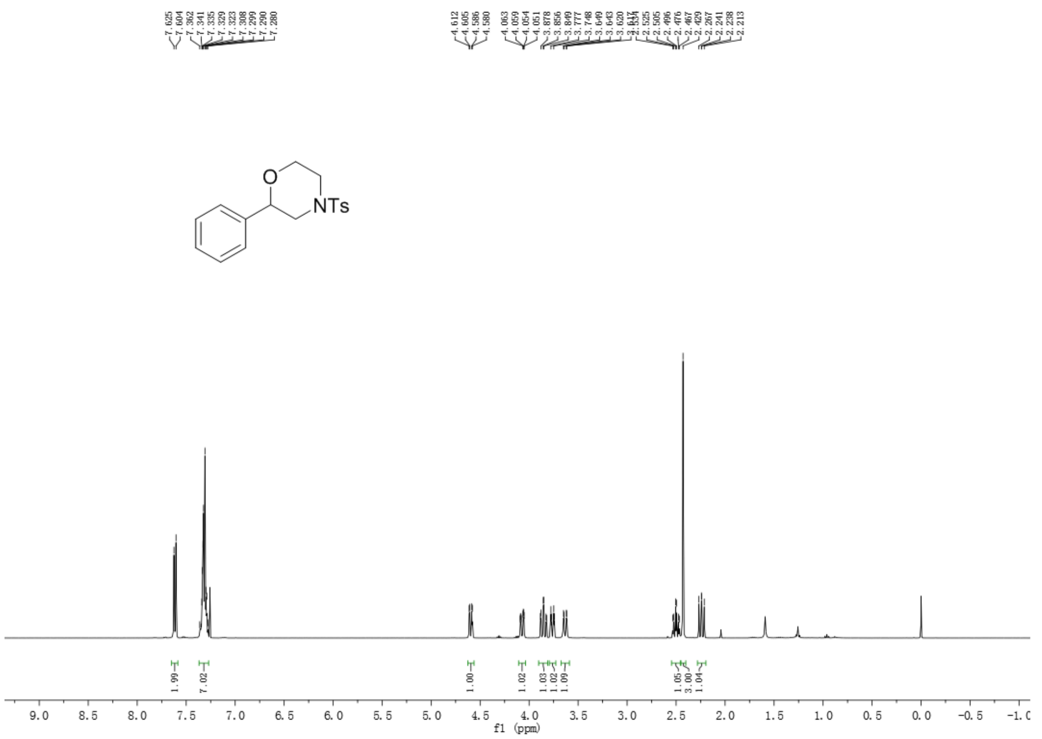

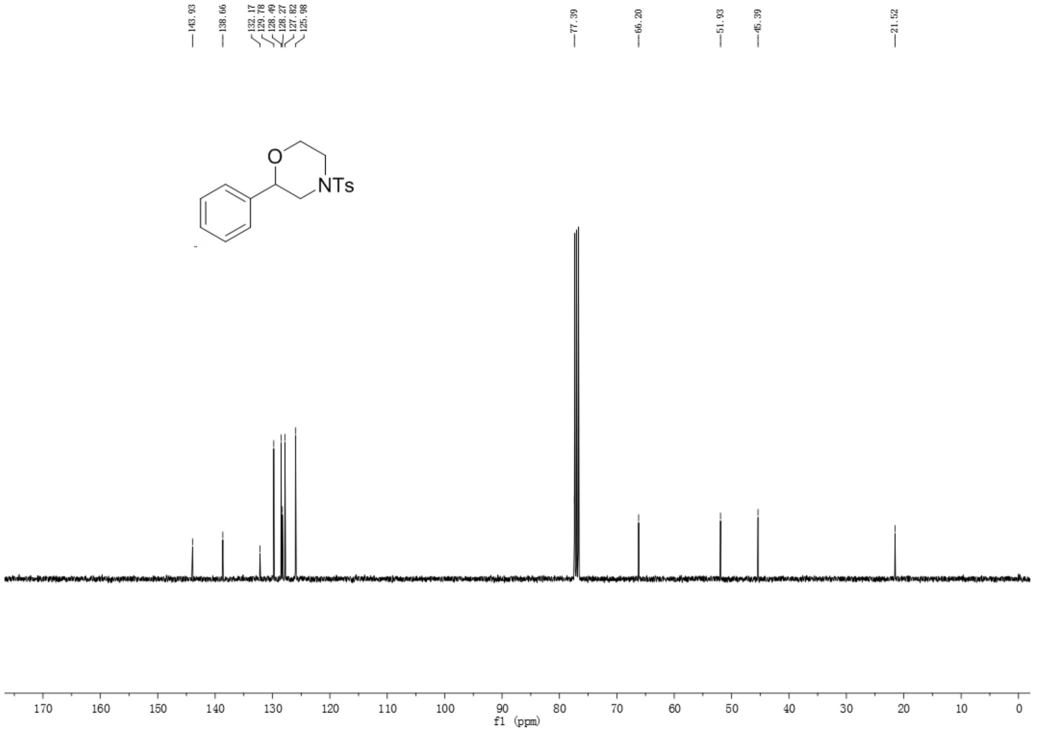

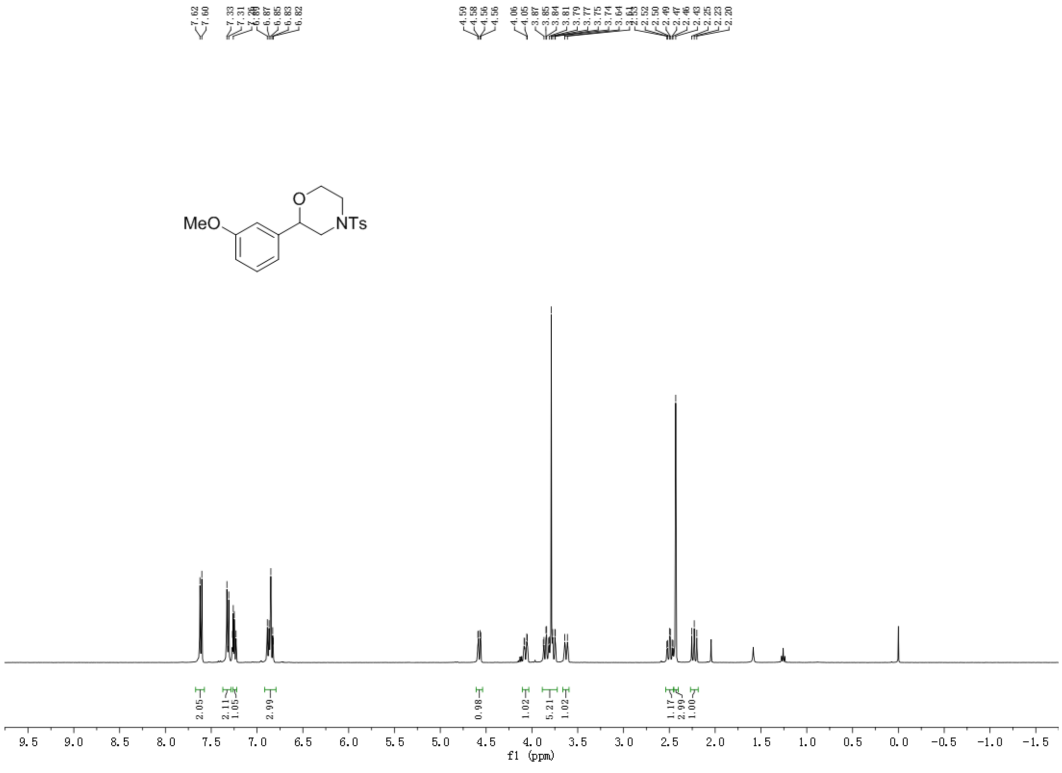
MeO
O
NTs
f1 (ppm)

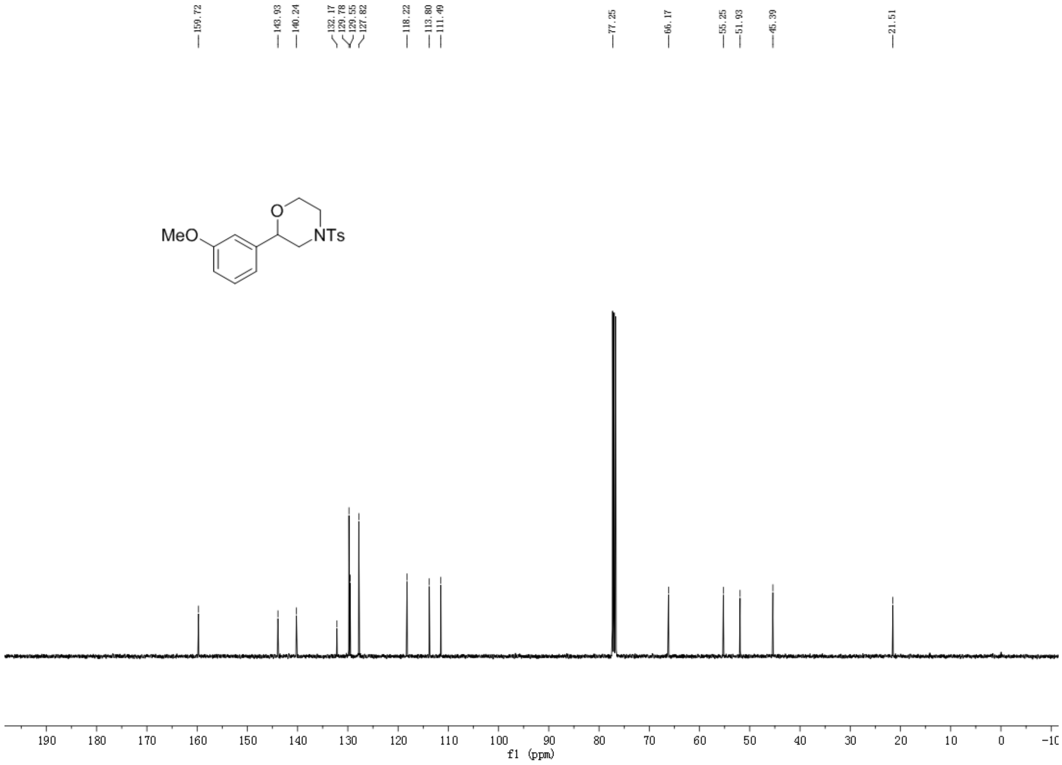
MeO
O
NTs
f1 (ppm)

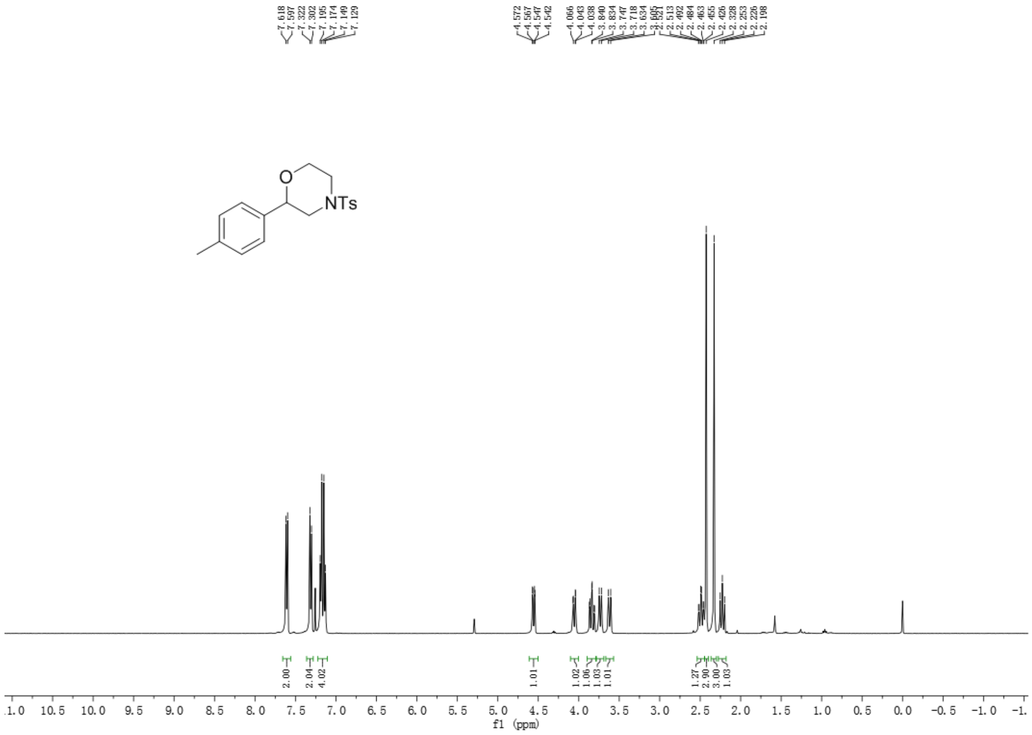
7.618
7.597
7.322
7.302
7.195
7.174
7.149
7.129
4.572
4.567
4.547
4.542
4.066
4.043
4.038
3.840
3.834
3.747
3.718
3.634
2.513
2.492
2.484
2.463
2.455
2.426
2.328
2.253
2.226
2.198
NTs
O
2.00
2.04
4.02
1.01
1.02
1.06
1.03
1.01
1.27
2.90
3.00
1.03
f1 (ppm)

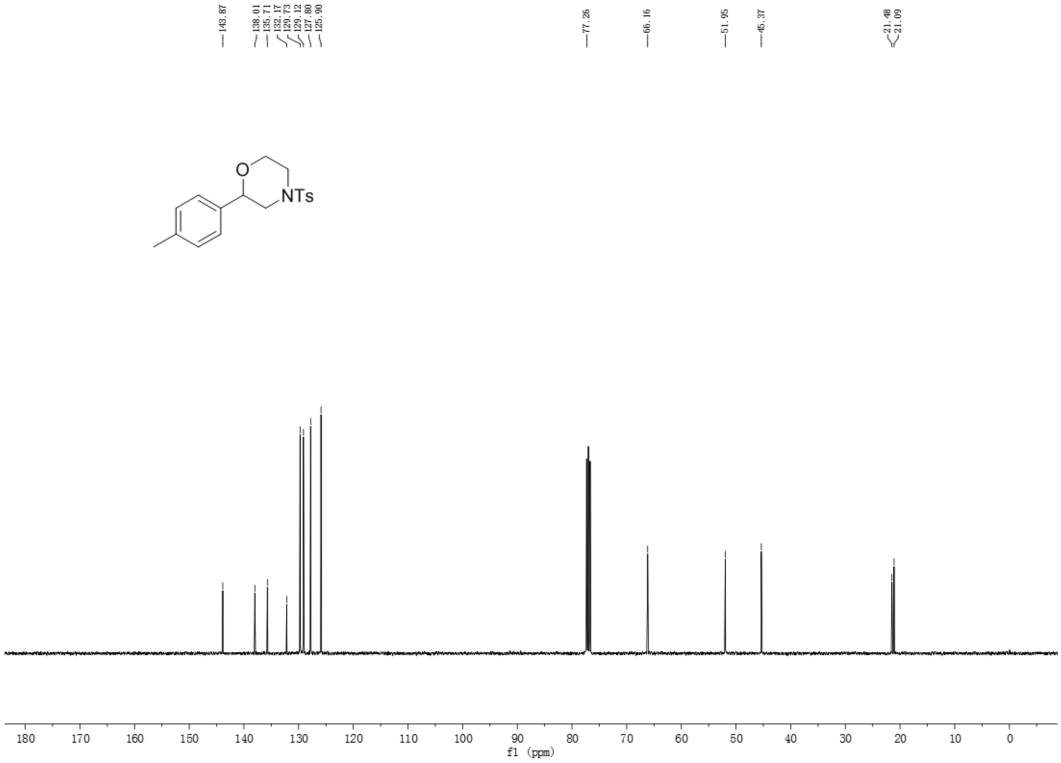
143.87
138.01
135.71
132.17
129.73
129.12
127.80
125.90
77.26
66.16
51.95
45.37
21.48
21.09
NTs
O
f1 (ppm)

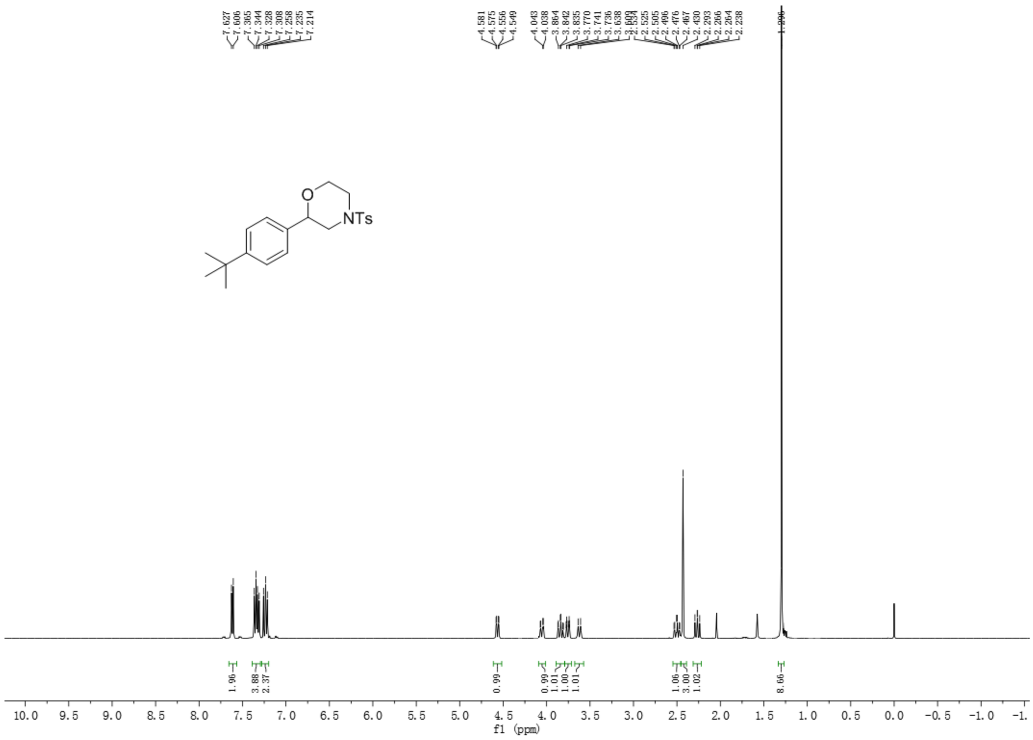
O
NTs
f1 (ppm)

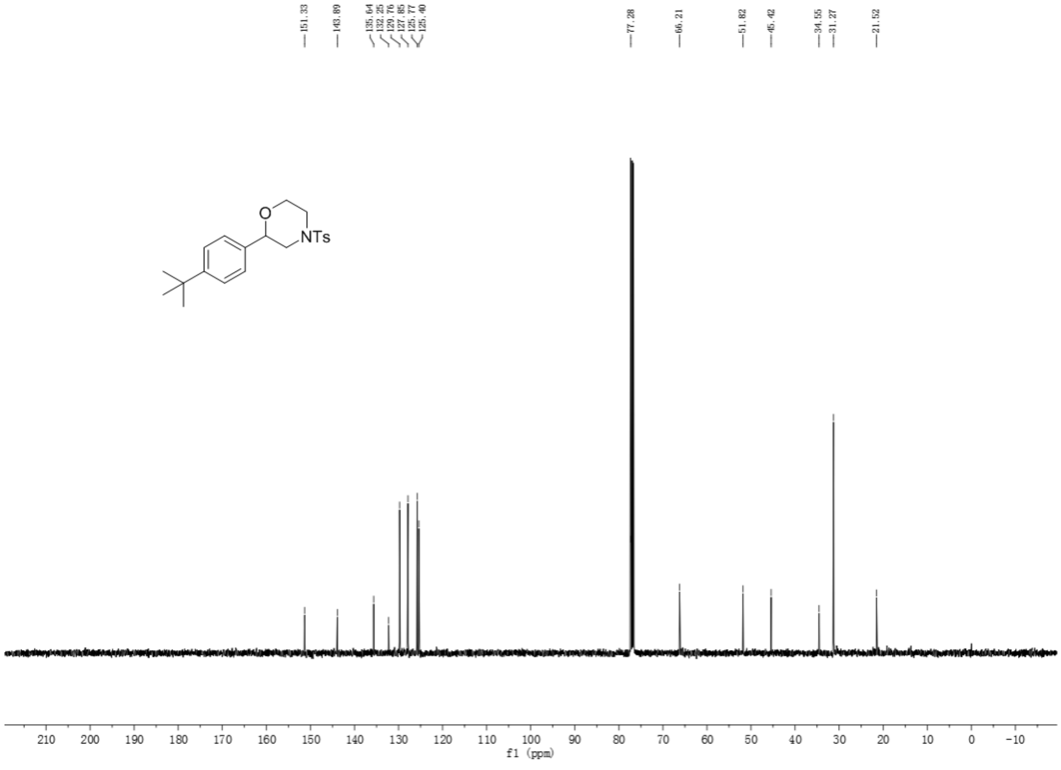
O
NTs
f1 (ppm)

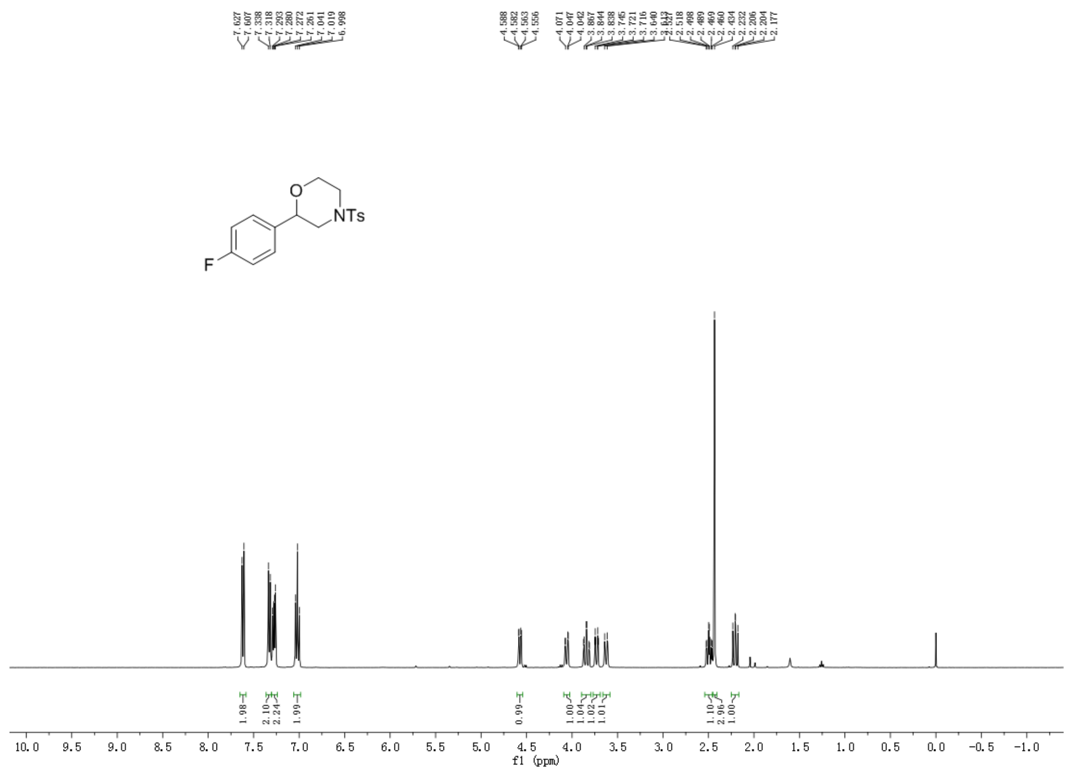

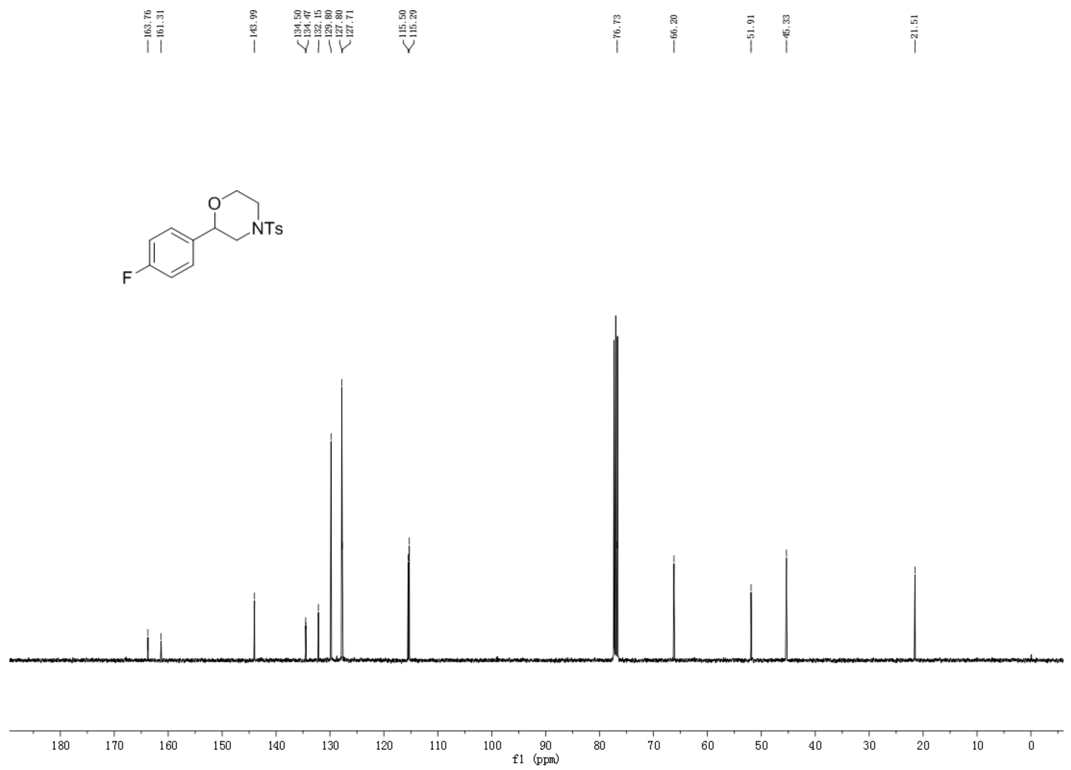

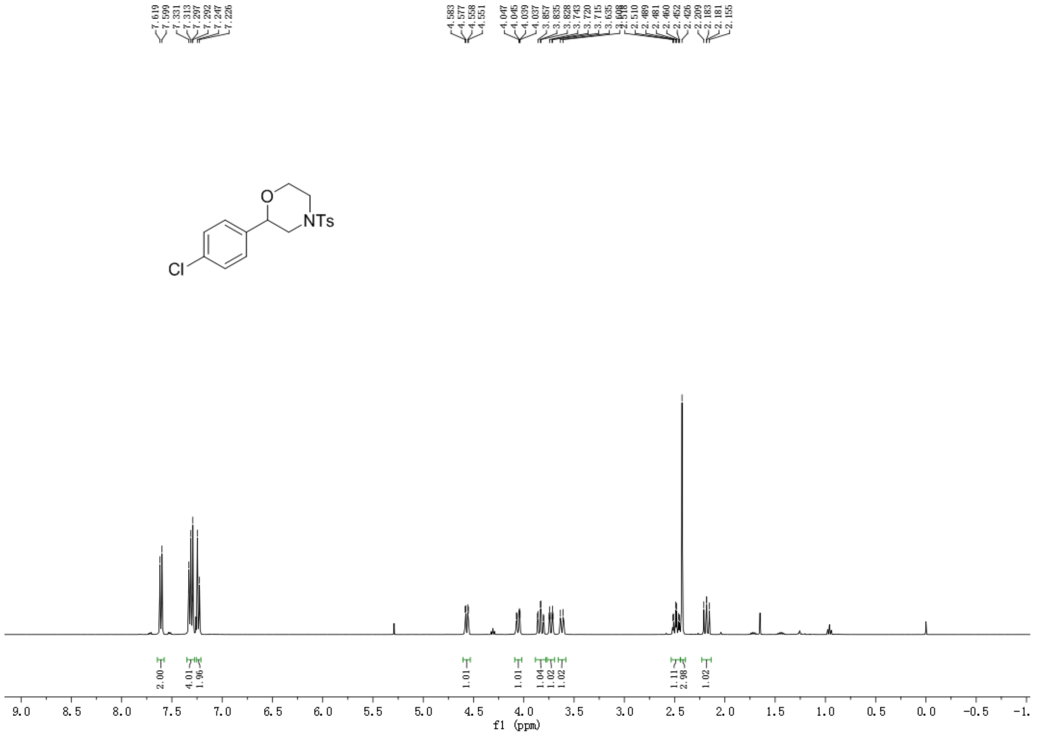

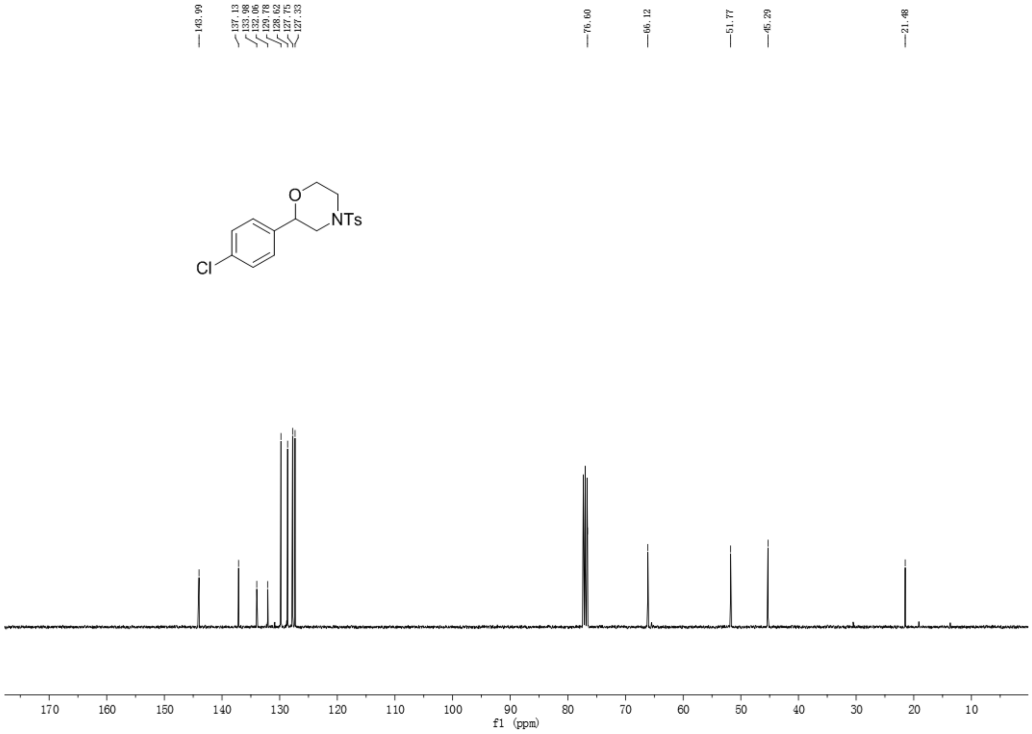

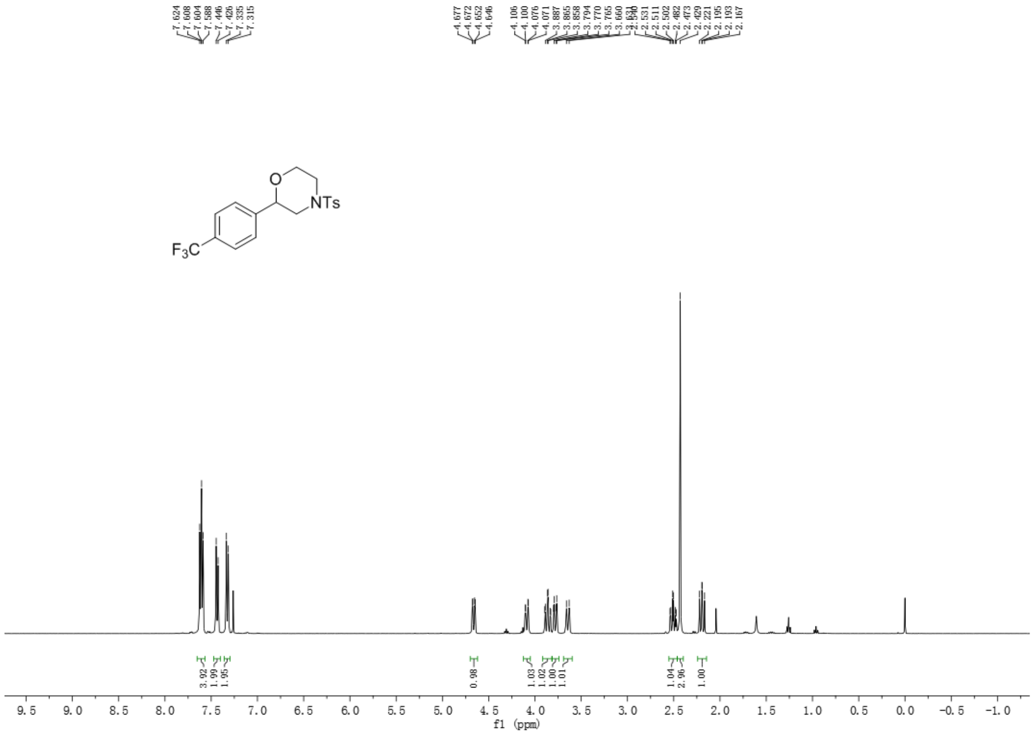
O
NTs
F3C
f1 (ppm)

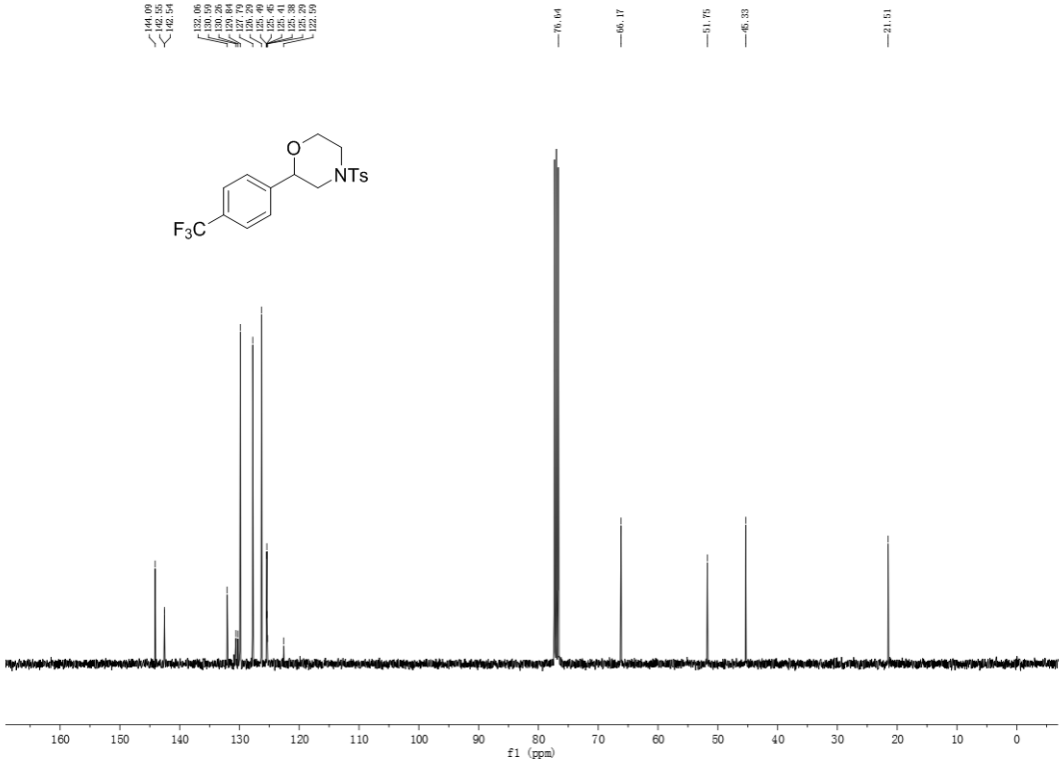
O
NTs
F3C
f1 (ppm)

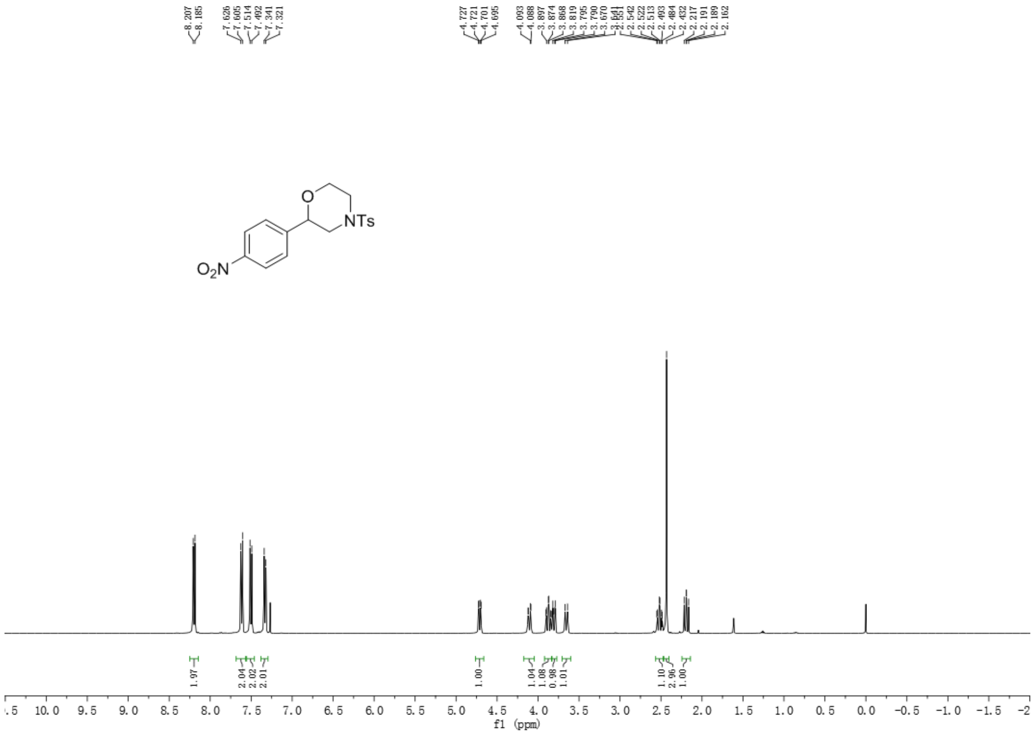
O
NTs
O2N
f1 (ppm)

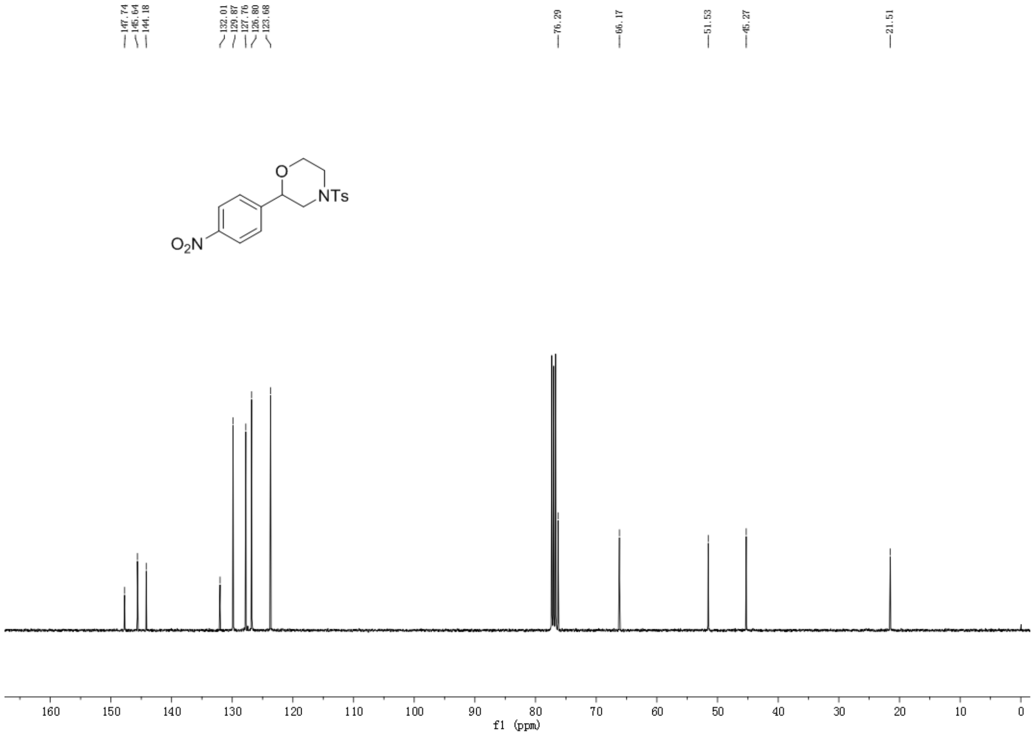
147.74
145.64
144.18
132.01
129.87
127.76
126.80
123.68
76.29
66.17
51.53
45.27
21.51
O
NTs
O2N
f1 (ppm)

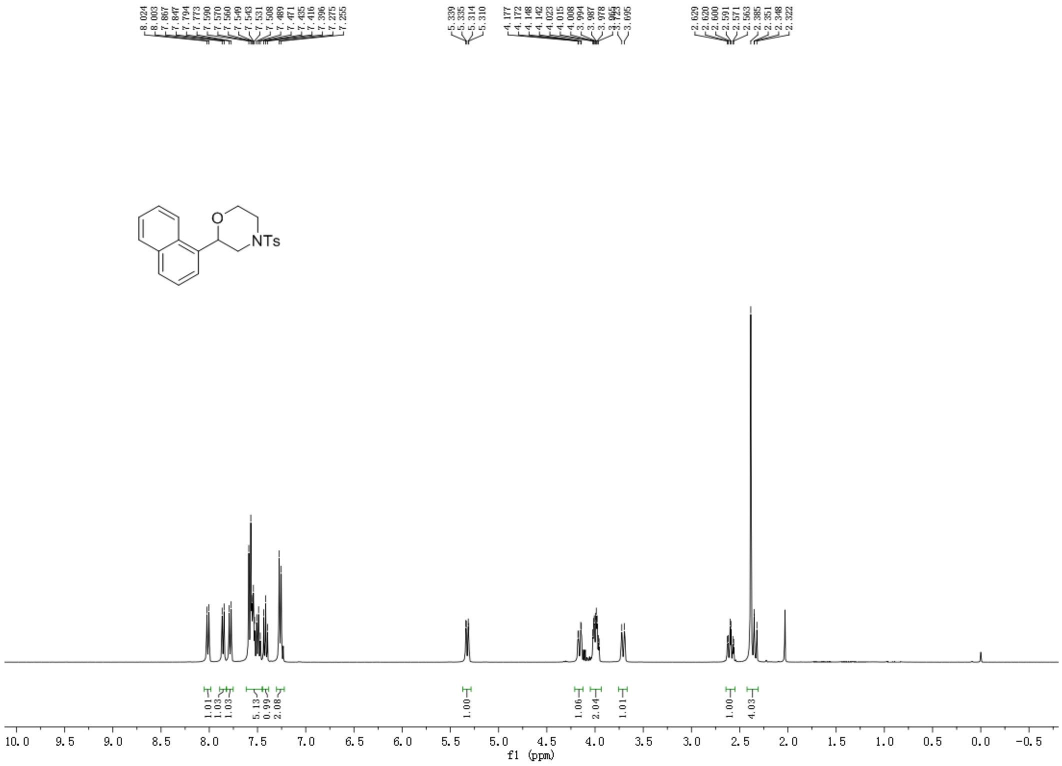

8.024
8.003
7.867
7.847
7.794
7.773
7.590
7.570
7.560
7.549
7.543
7.531
7.508
7.489
7.471
7.435
7.416
7.396
7.275
7.255
5.339
5.335
5.314
5.310
4.177
4.172
4.148
4.142
4.023
4.015
4.008
3.994
3.987
3.978
3.695
2.629
2.620
2.600
2.591
2.571
2.563
2.385
2.351
2.348
2.322
O
NTs
1.01
1.03
1.03
5.13
0.99
2.08
1.00
1.06
2.04
1.01
1.00
4.03
f1 (ppm)

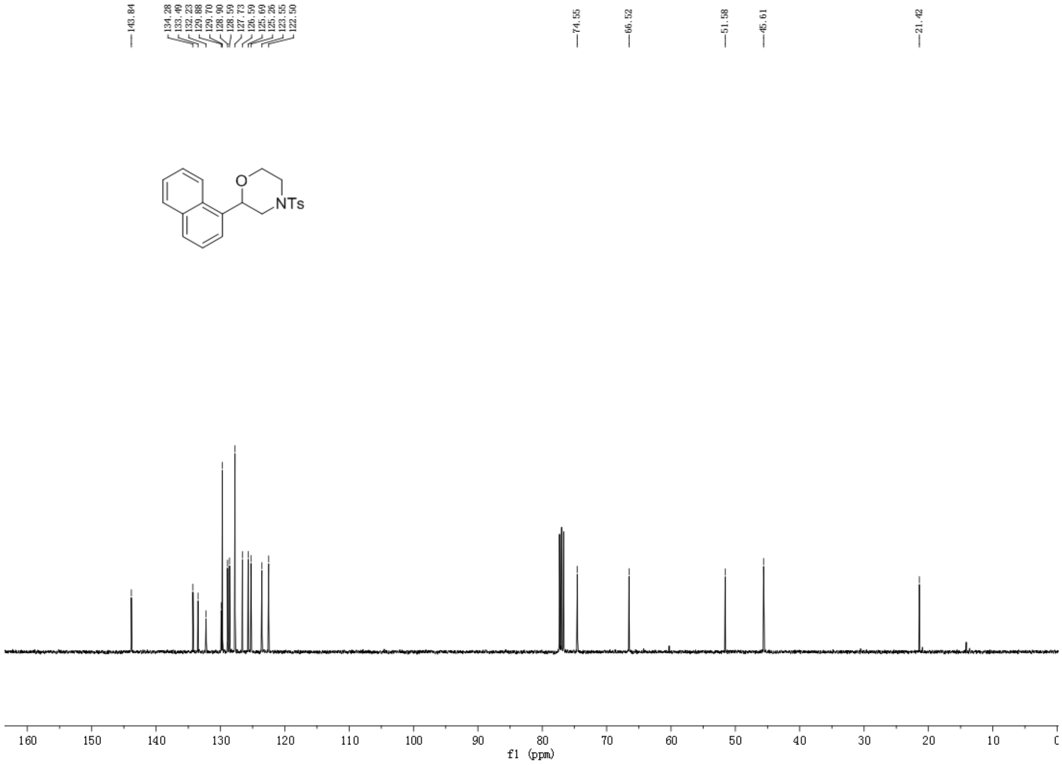

143.84
134.28
133.49
132.23
129.88
129.70
128.90
128.59
127.73
126.59
125.69
125.26
123.55
122.50
74.55
66.52
51.58
45.61
21.42
O
NTs
f1 (ppm)

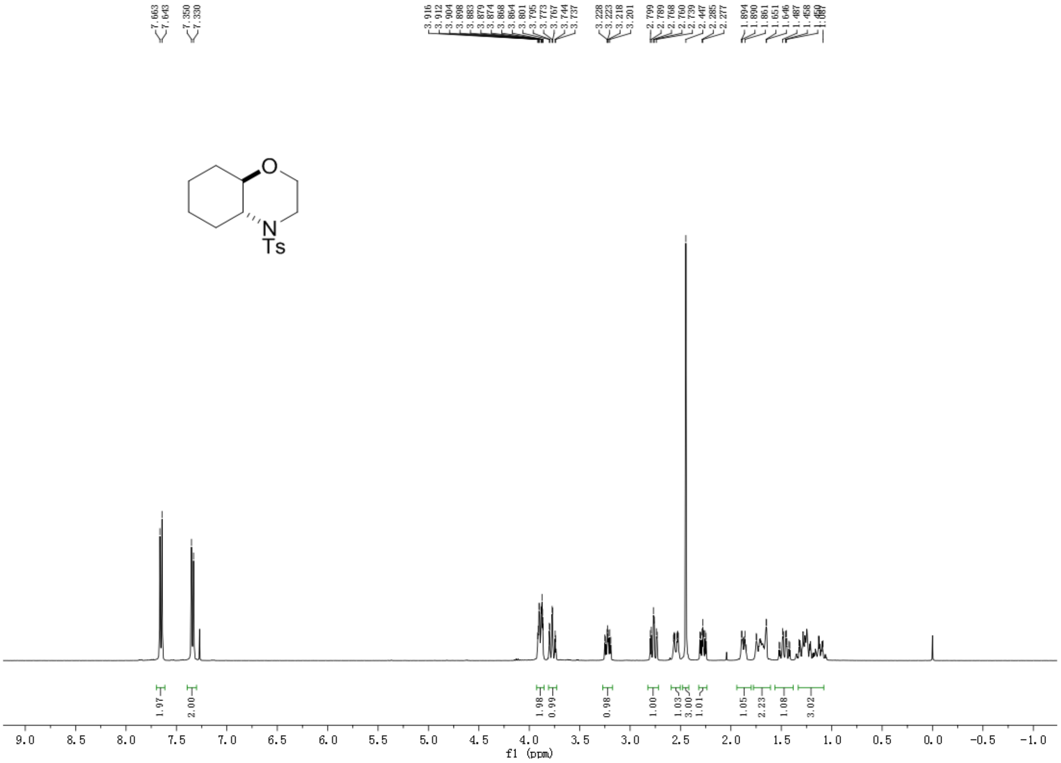
O
N
Ts
f1 (ppm)

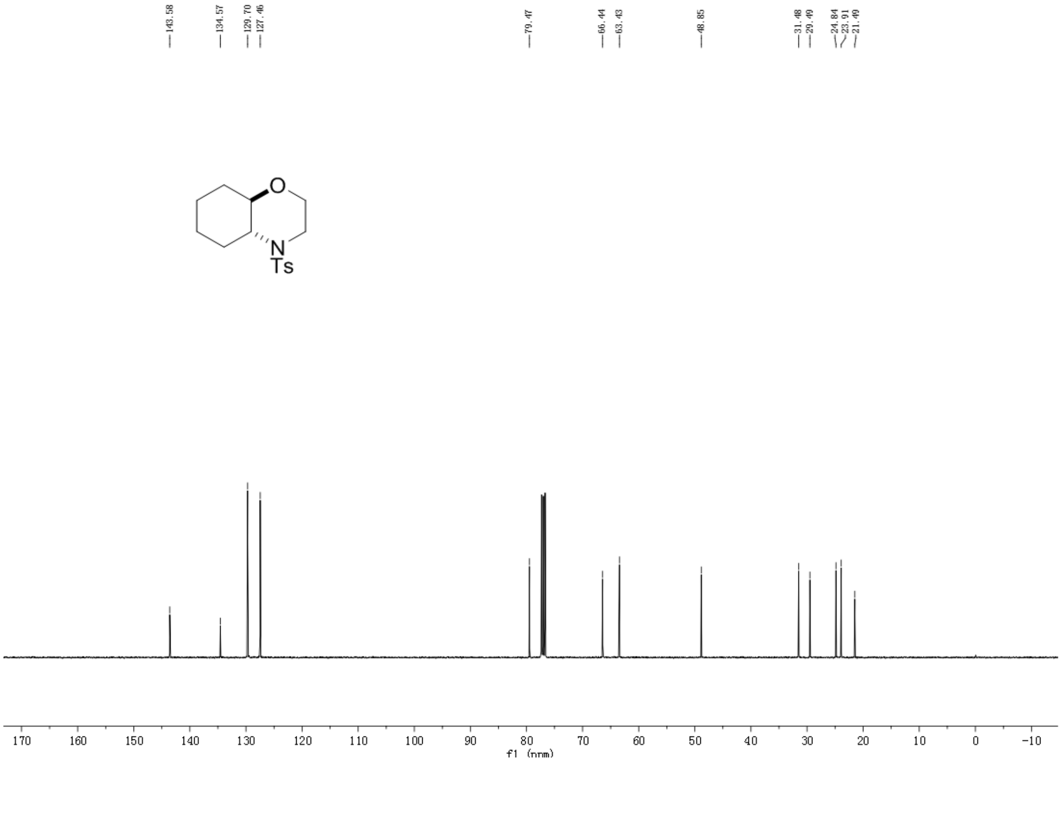
O
N
Ts
f1 (ppm)

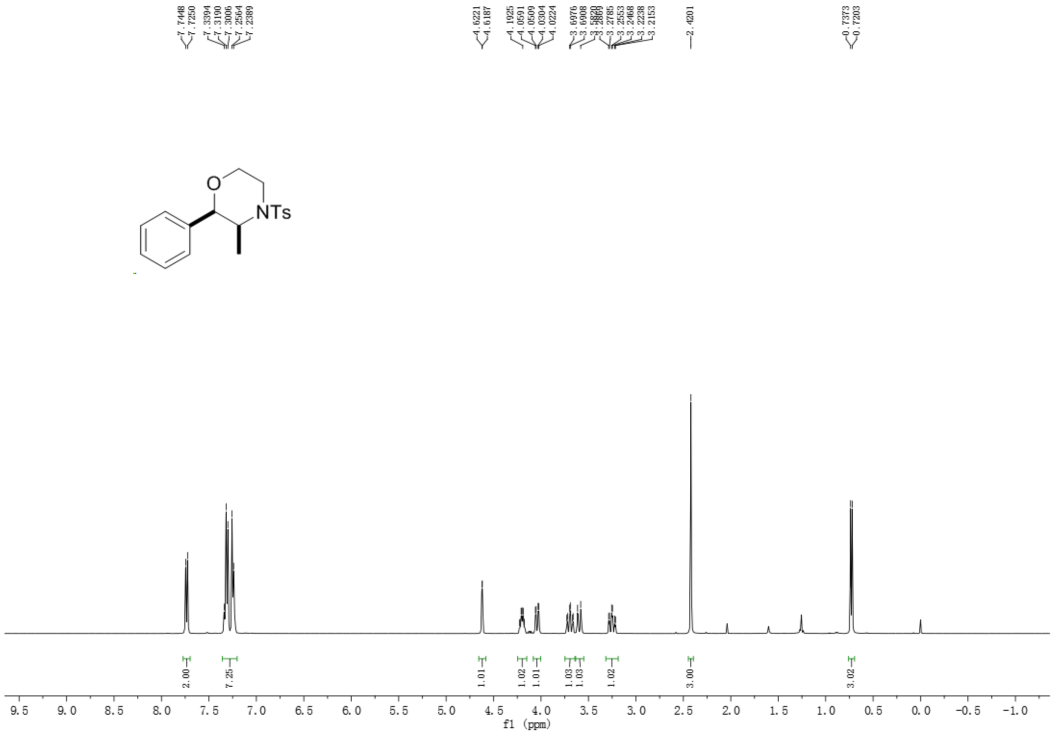

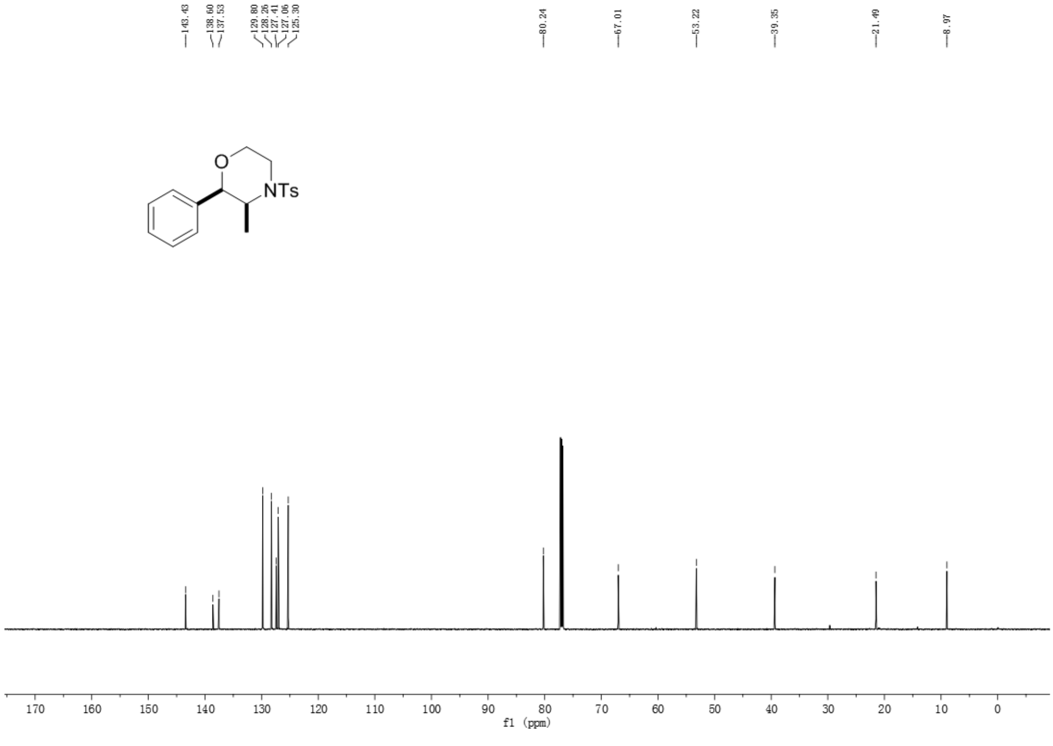

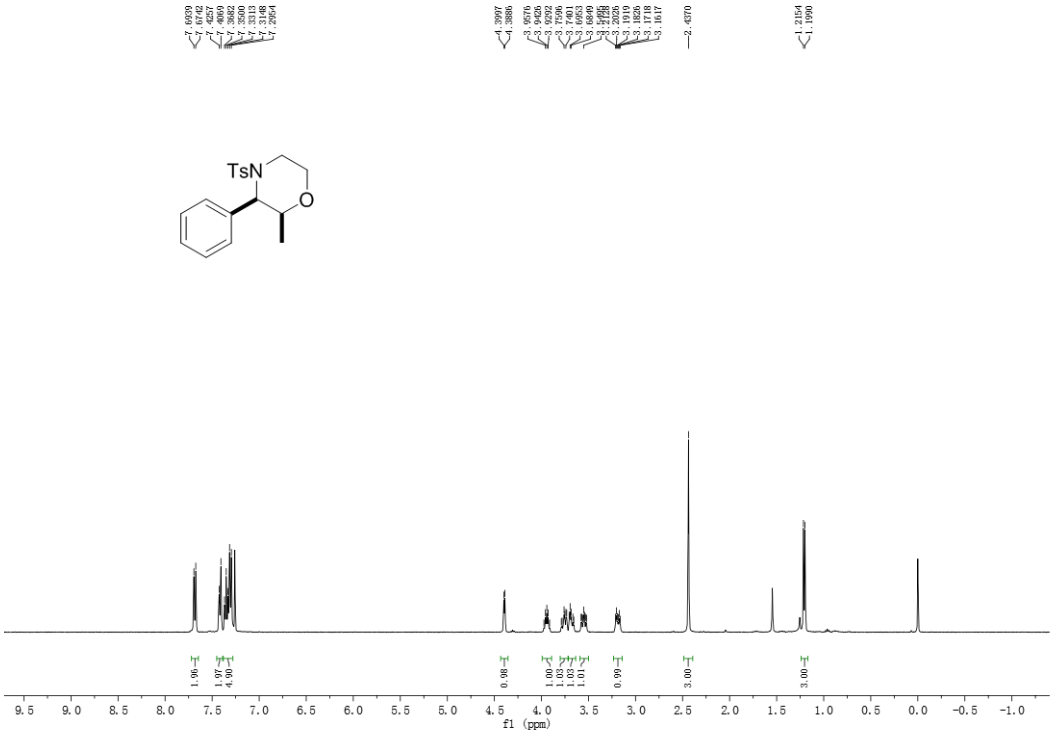

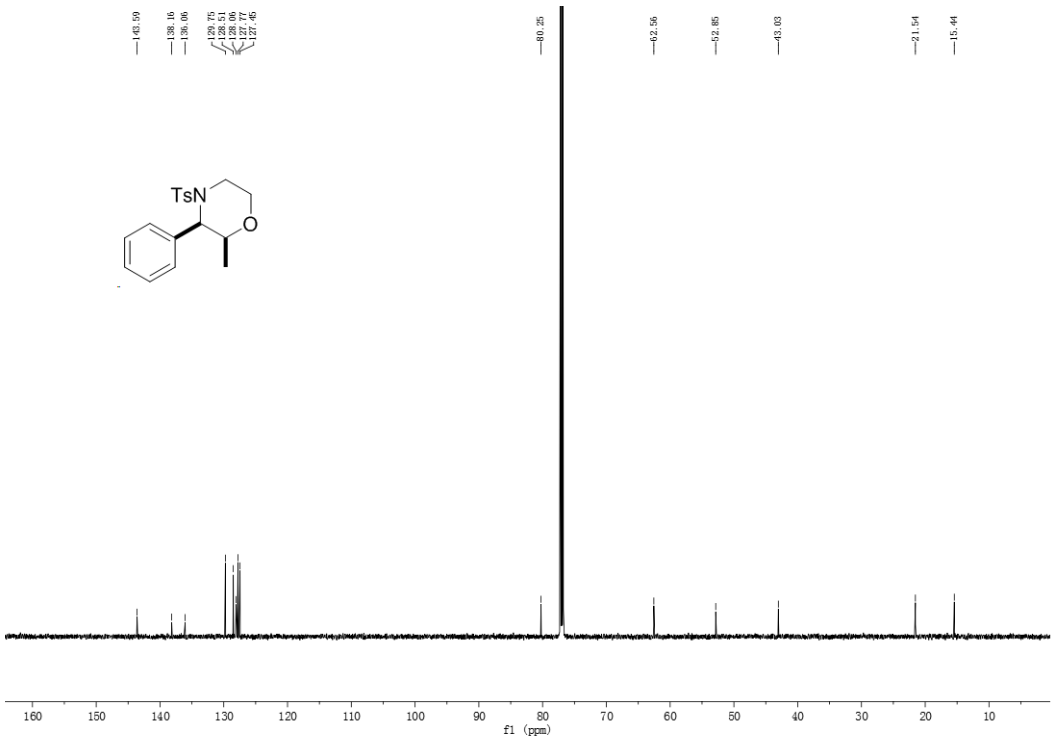

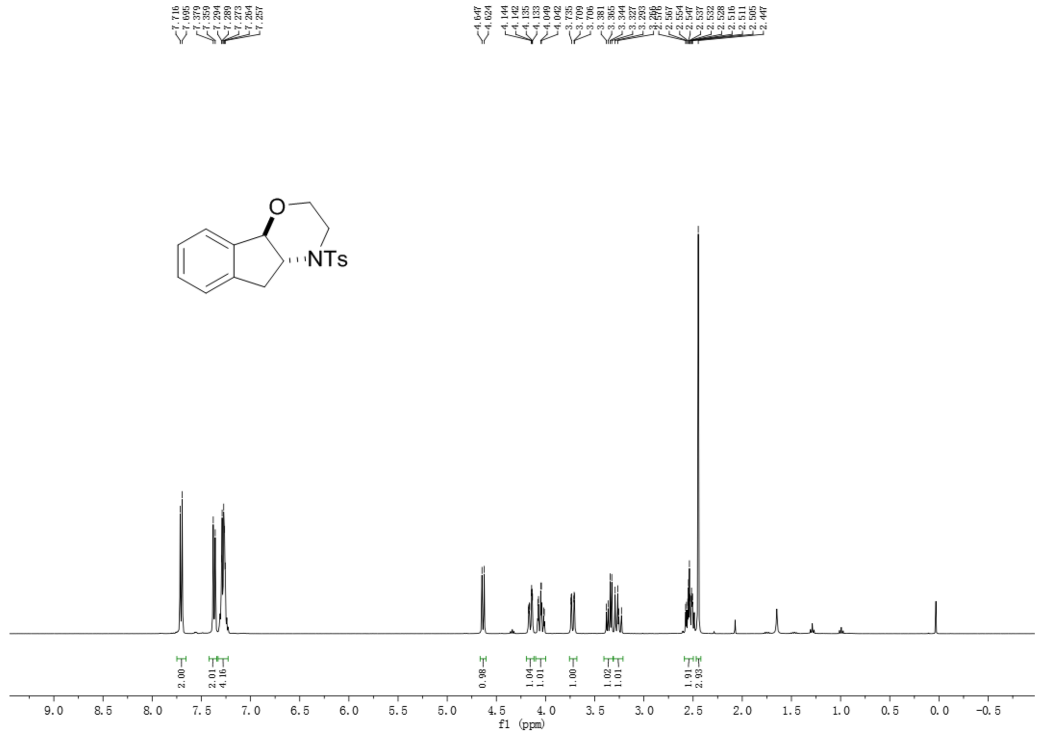

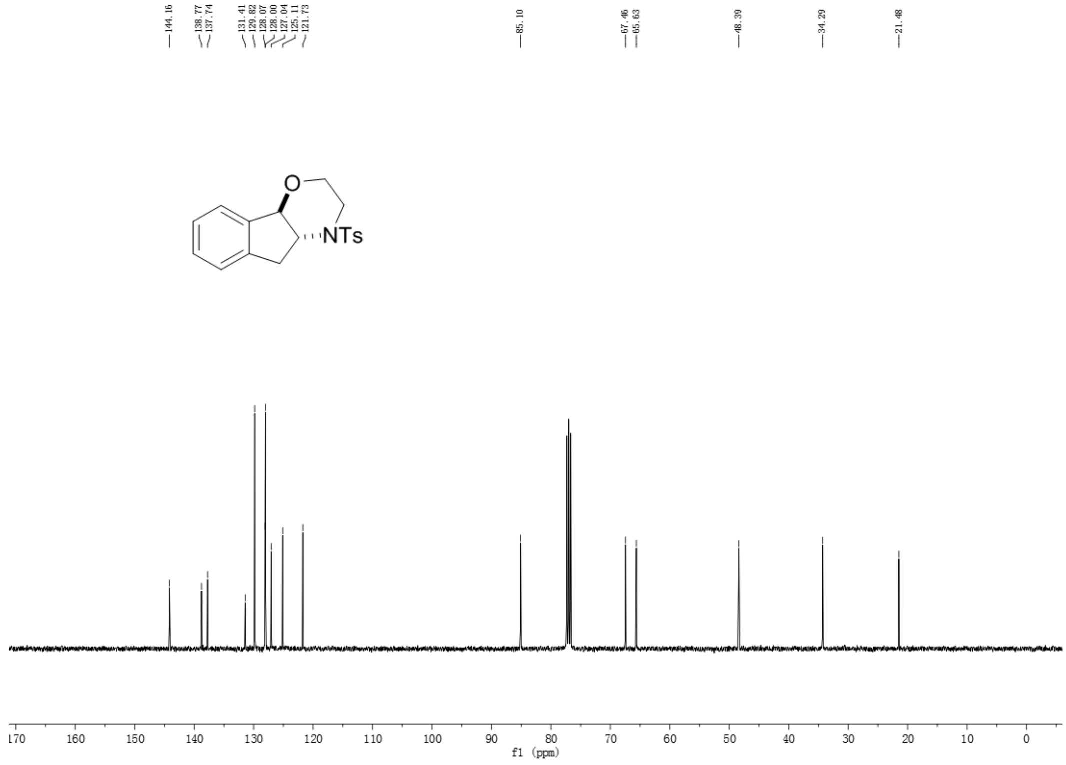

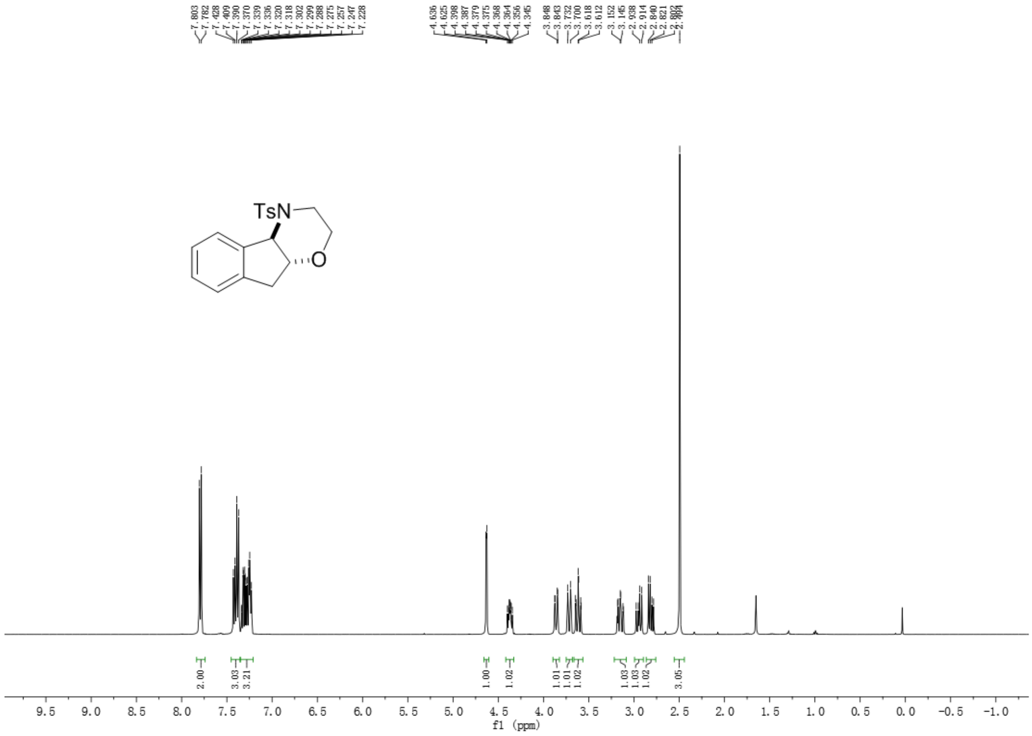

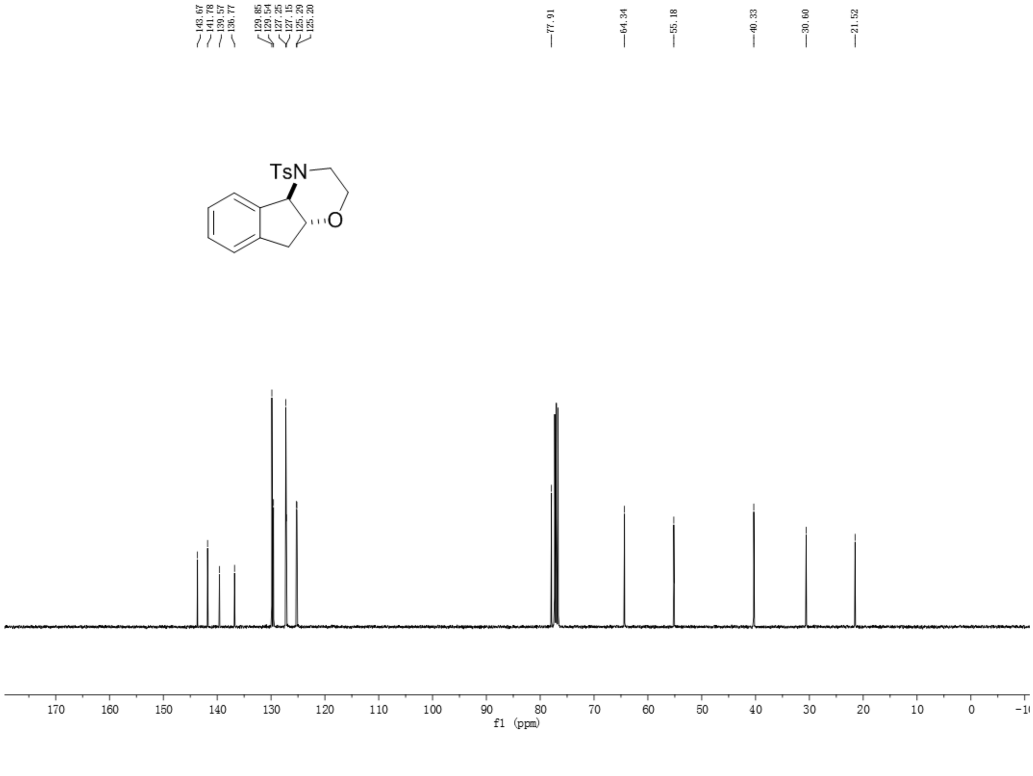

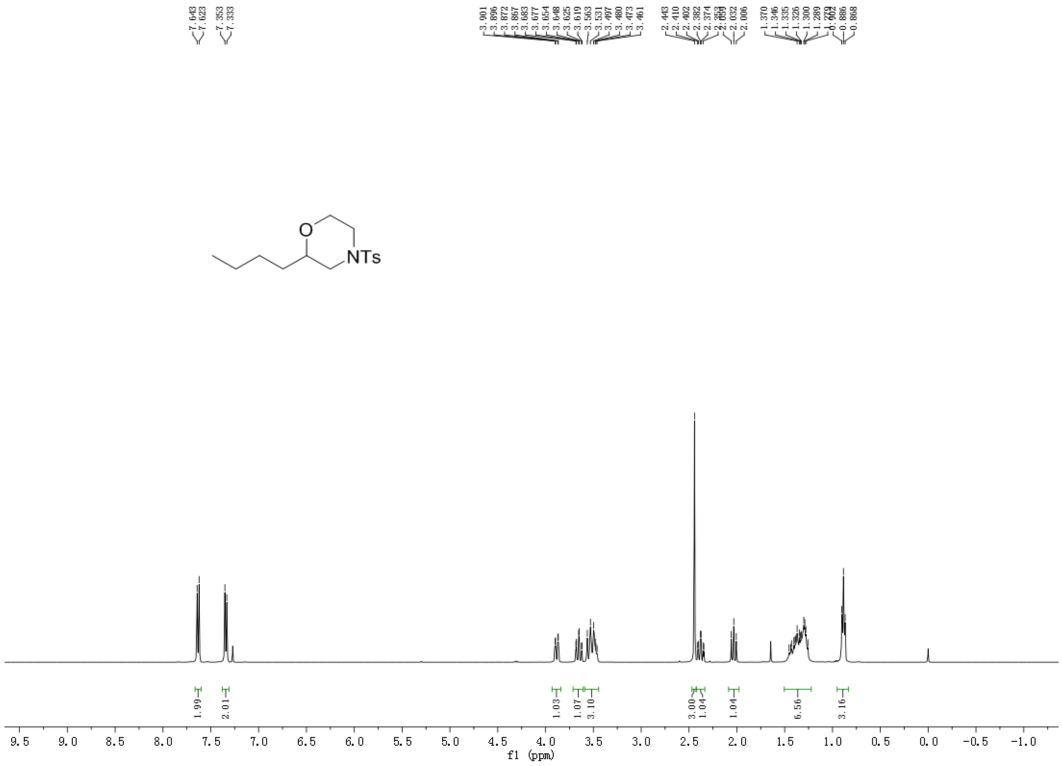

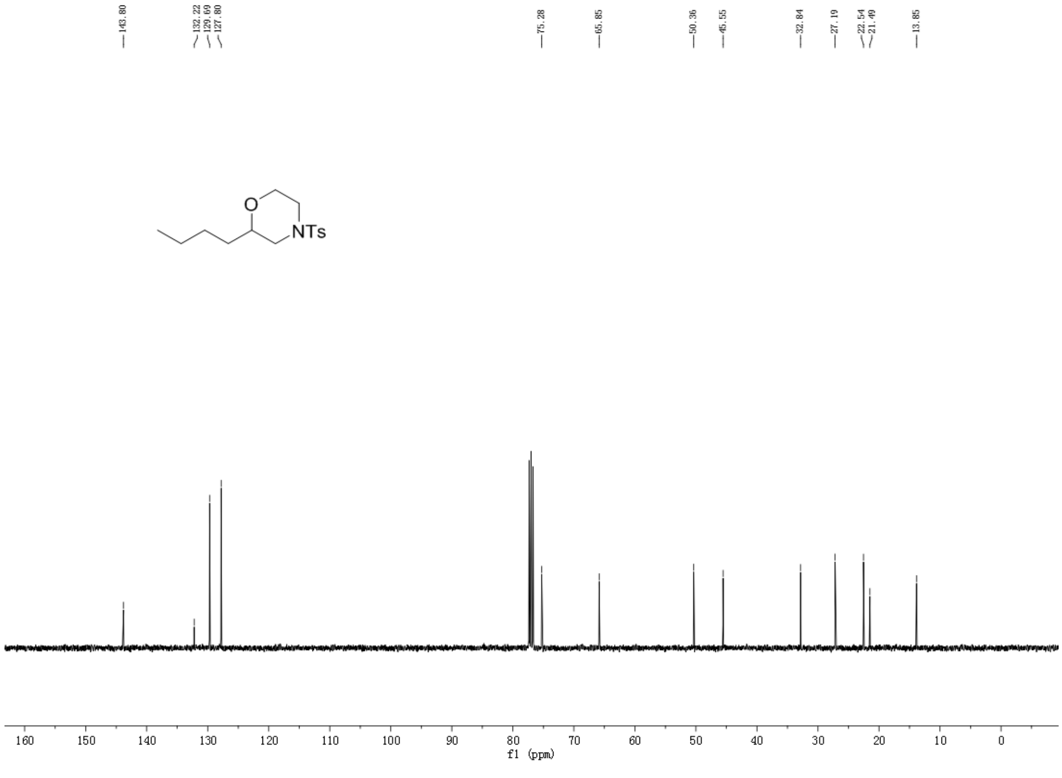

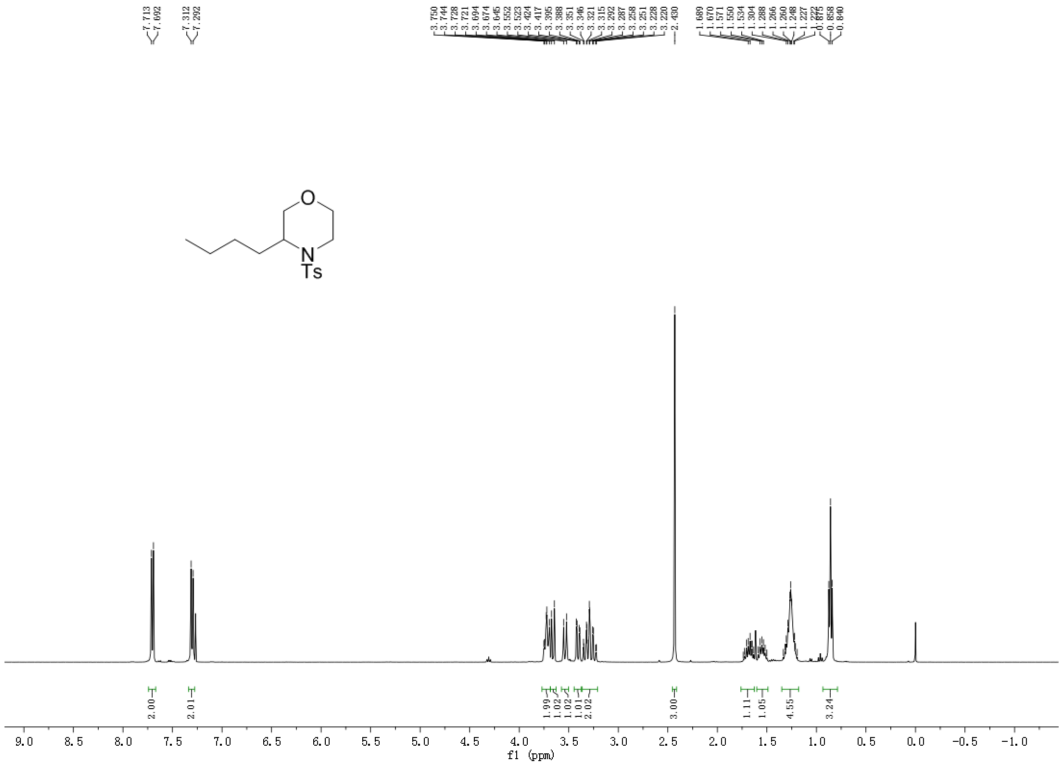
O
N
Ts
f1 (ppm)

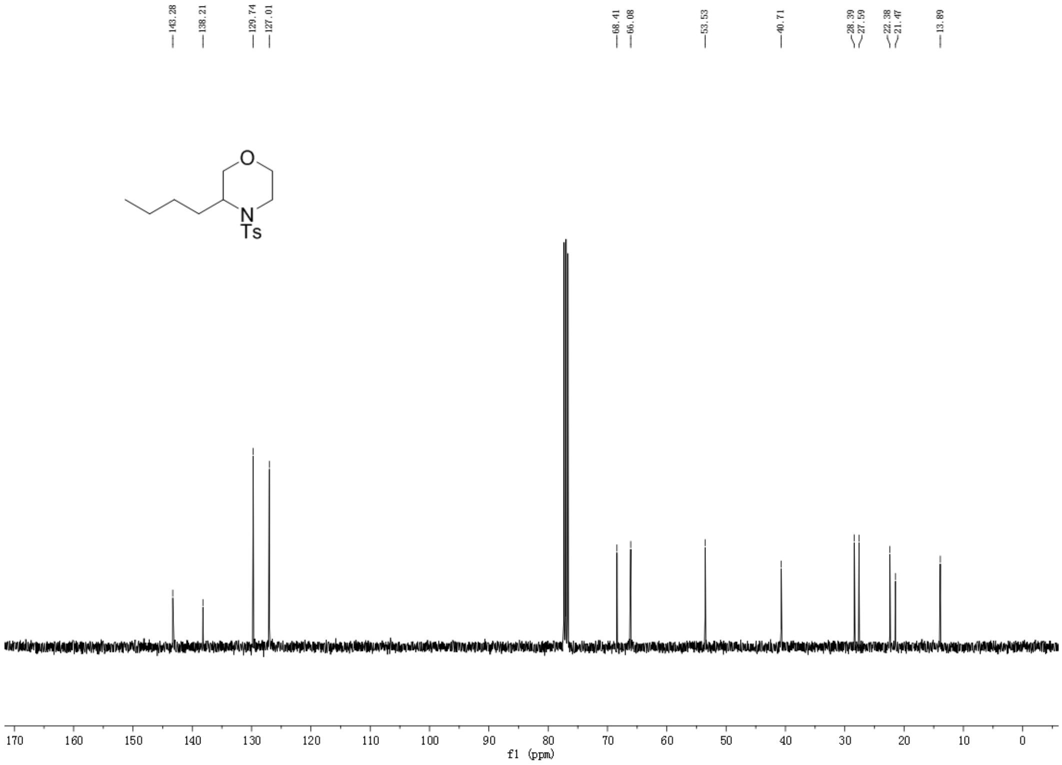
143.28
138.21
129.74
127.01
68.41
66.08
53.53
40.71
28.39
27.59
22.38
21.47
13.89
O
N
Ts
f1 (ppm)

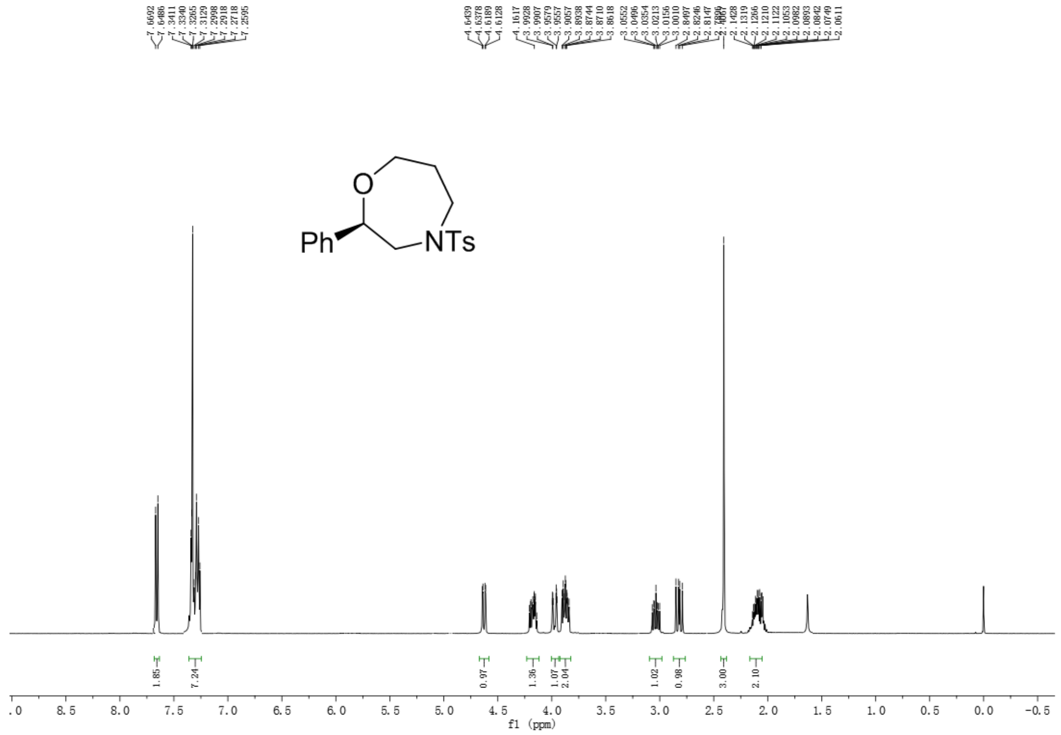

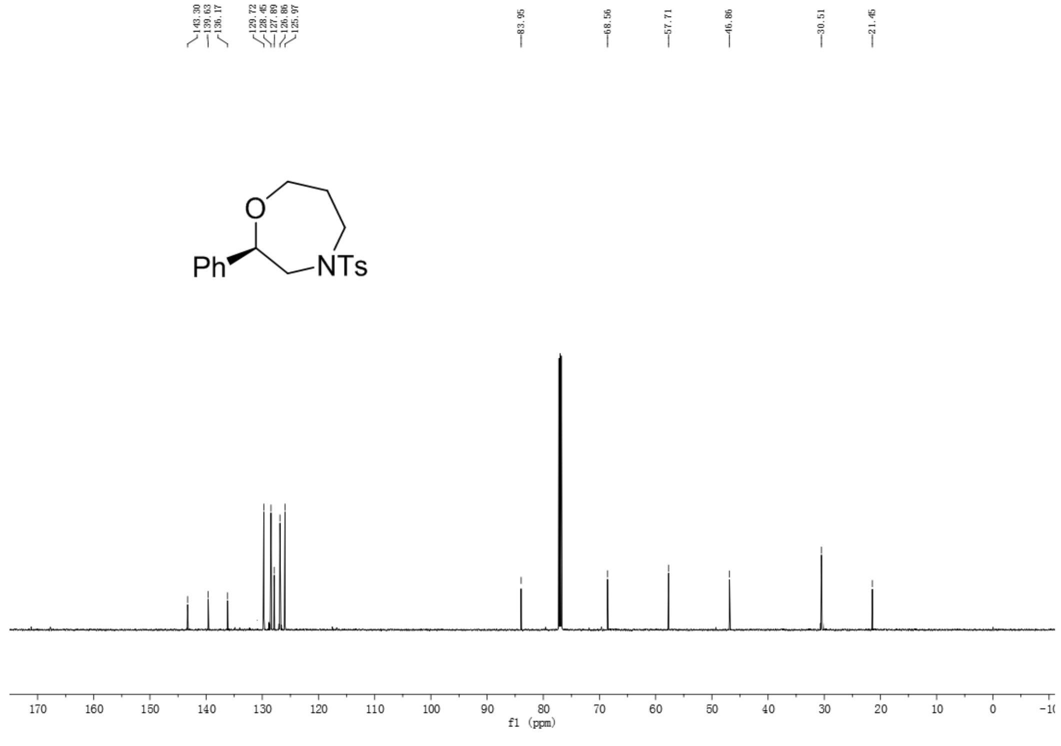

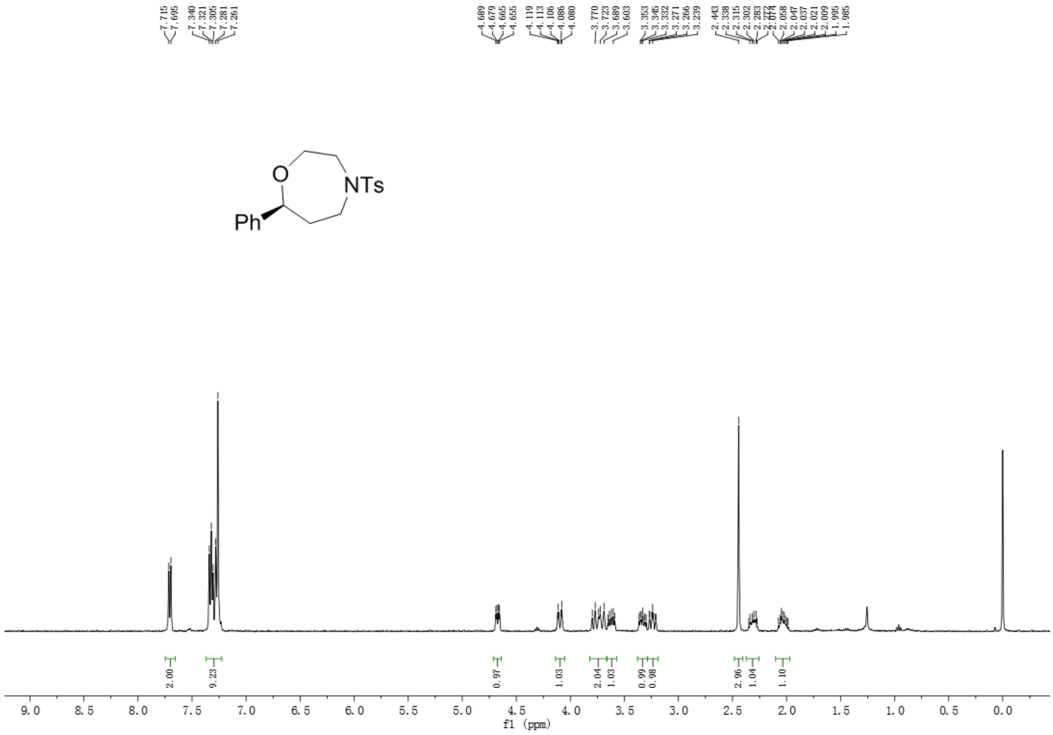

7.715
7.695
7.340
7.321
7.305
7.281
7.261
4.689
4.679
4.665
4.655
4.119
4.113
4.106
4.086
4.080
3.770
3.723
3.689
3.603
3.353
3.345
3.332
3.271
3.266
3.239
2.443
2.338
2.315
2.302
2.283
2.058
2.047
2.037
2.021
2.009
1.995
1.985
O
NTs
Ph
2.00
9.23
0.97
1.03
2.04
1.03
0.99
0.98
2.96
1.04
1.10
9.0 8.5 8.0 7.5 7.0 6.5 6.0 5.5 5.0 4.5 4.0 3.5 3.0 2.5 2.0 1.5 1.0 0.5 0.0
f1 (ppm)

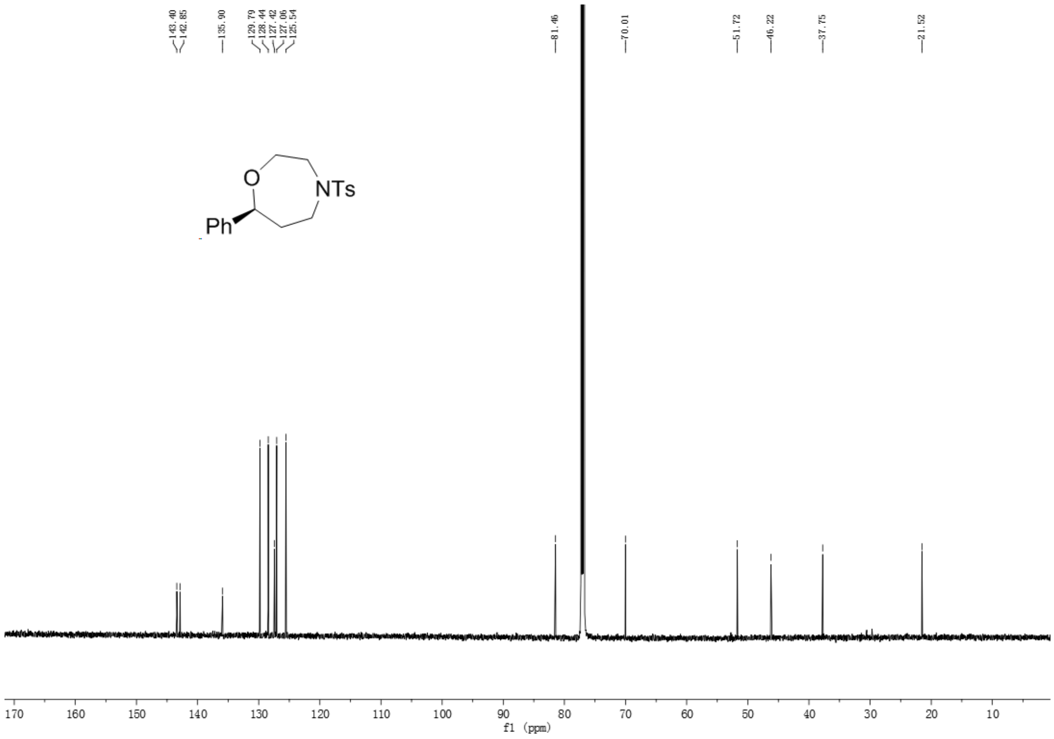

143.40
142.85
135.90
129.79
128.44
127.42
127.06
125.54
81.46
70.01
51.72
46.22
37.75
21.52
O
NTs
Ph
170 160 150 140 130 120 110 100 90 80 70 60 50 40 30 20 10
f1 (ppm)

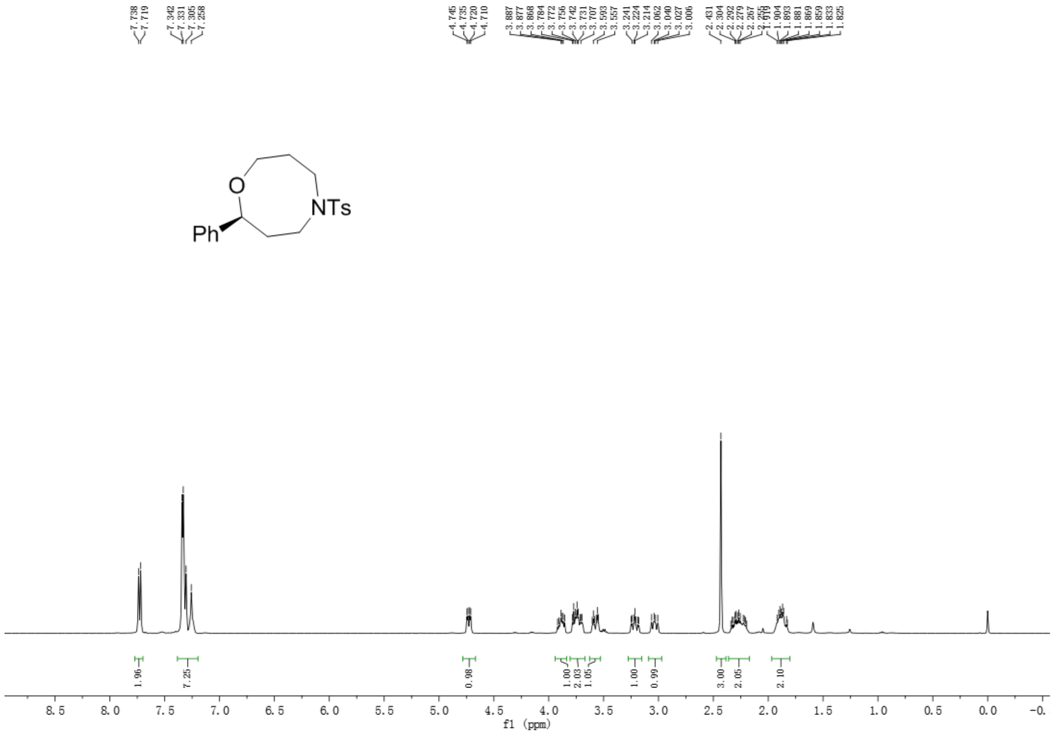

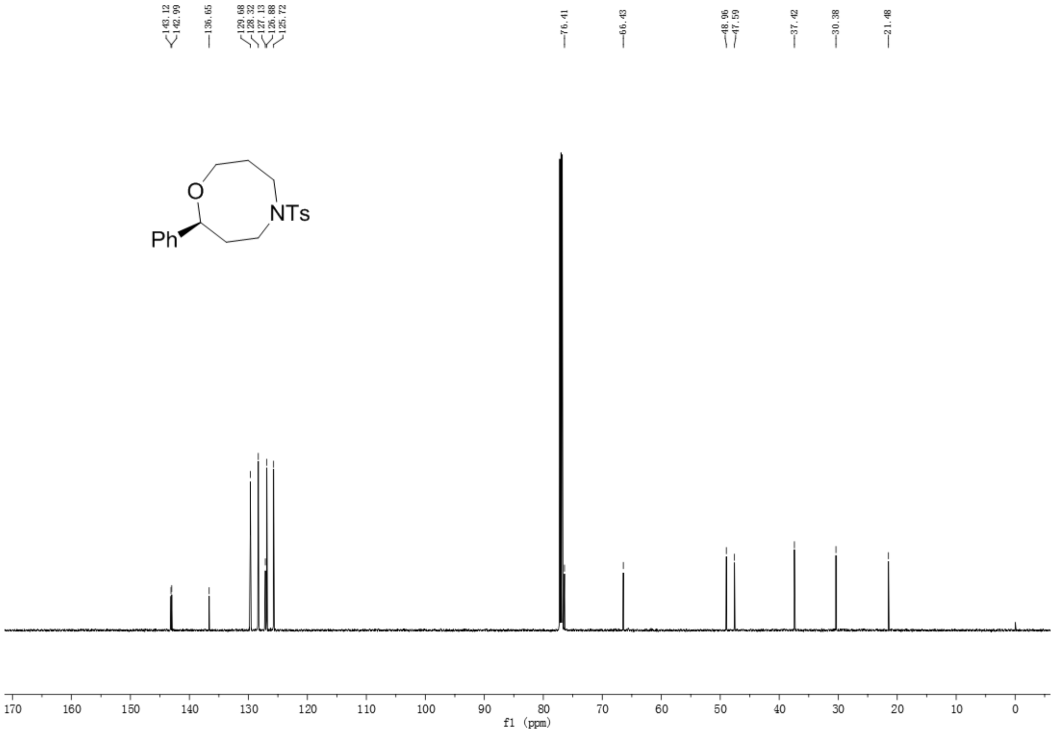

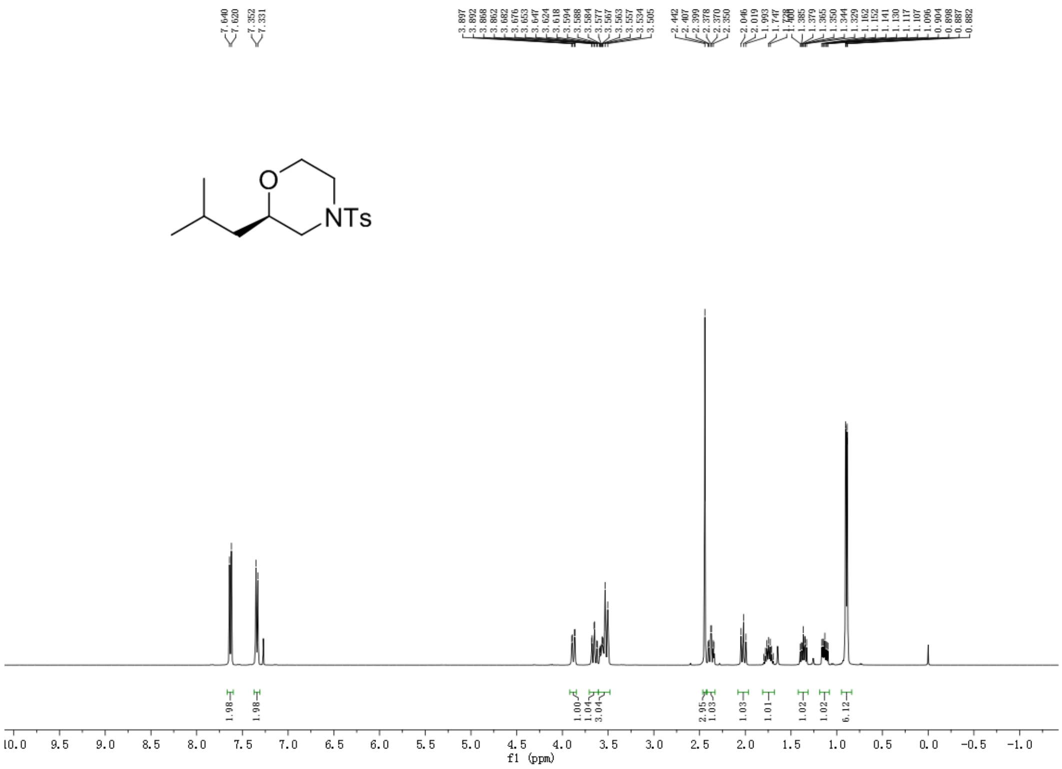
7.640
7.620
7.352
7.331
3.897
3.892
3.868
3.862
3.682
3.676
3.653
3.647
3.624
3.618
3.594
3.588
3.584
3.577
3.567
3.563
3.557
3.534
3.505
2.442
2.407
2.399
2.378
2.370
2.350
2.046
2.019
1.993
1.747
1.385
1.379
1.365
1.350
1.344
1.329
1.162
1.152
1.141
1.130
1.117
1.107
1.096
0.904
0.898
0.887
0.882
O
NTs
1.98
1.98
1.00
1.04
3.04
2.95
1.03
1.03
1.01
1.02
1.02
6.12
f1 (ppm)

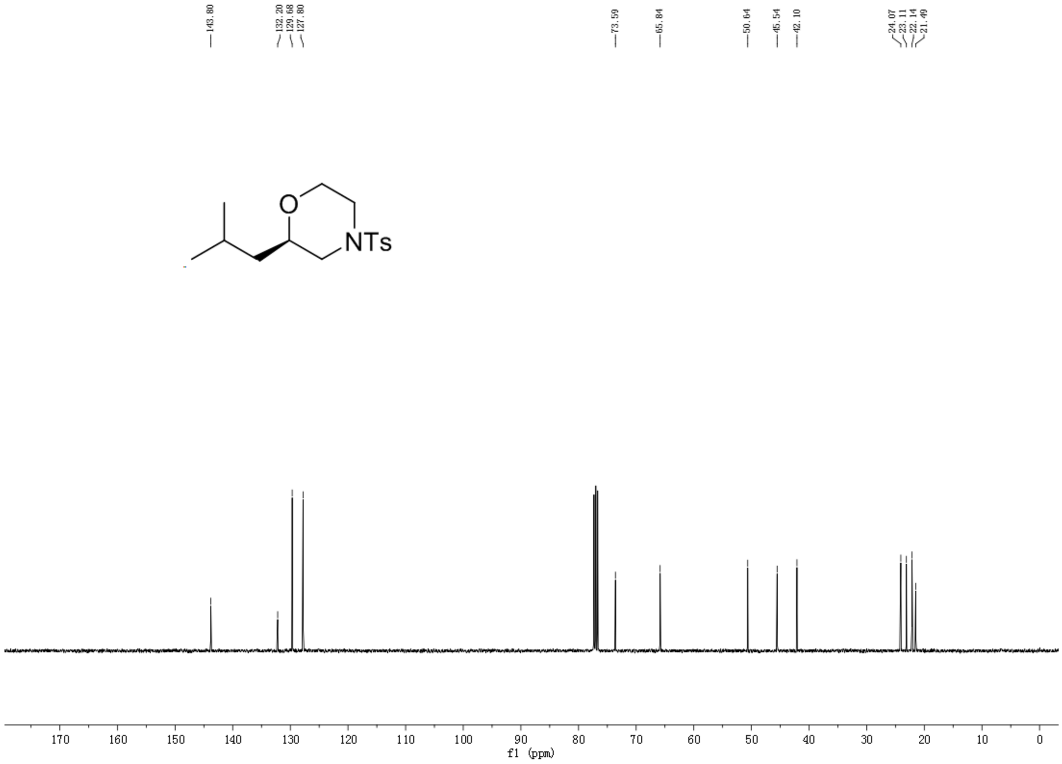
143.80
132.20
129.68
127.80
73.59
65.84
50.64
45.54
42.10
24.07
23.11
22.14
21.49
O
NTs
f1 (ppm)

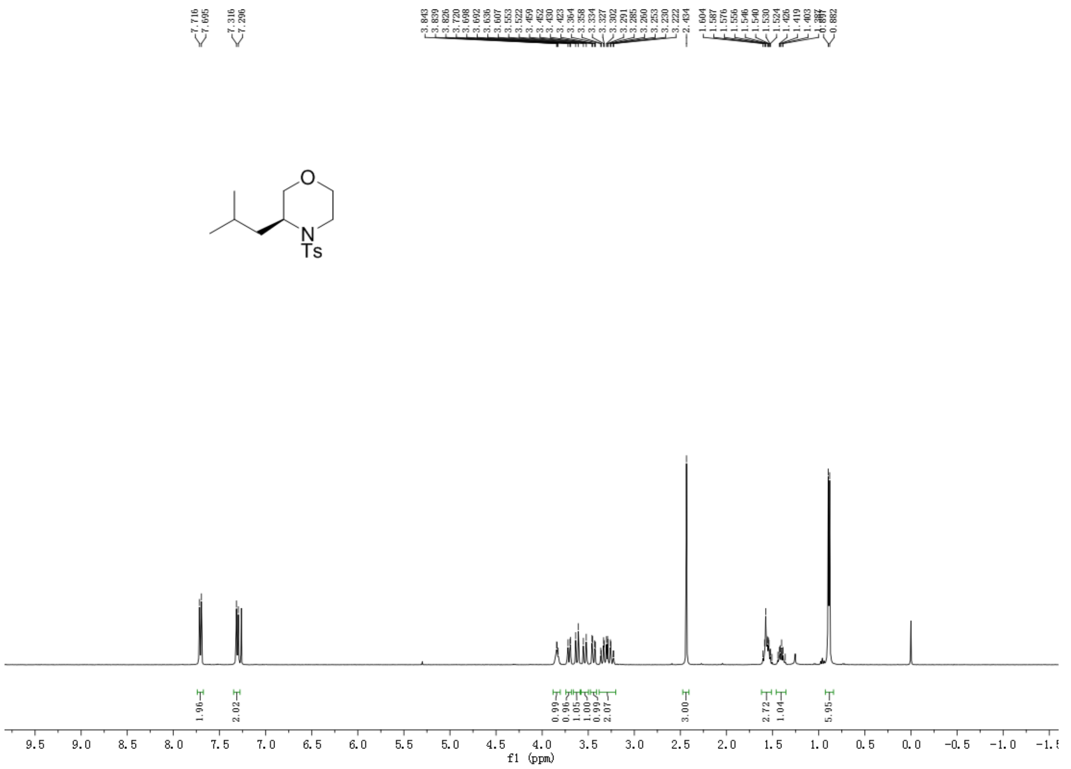

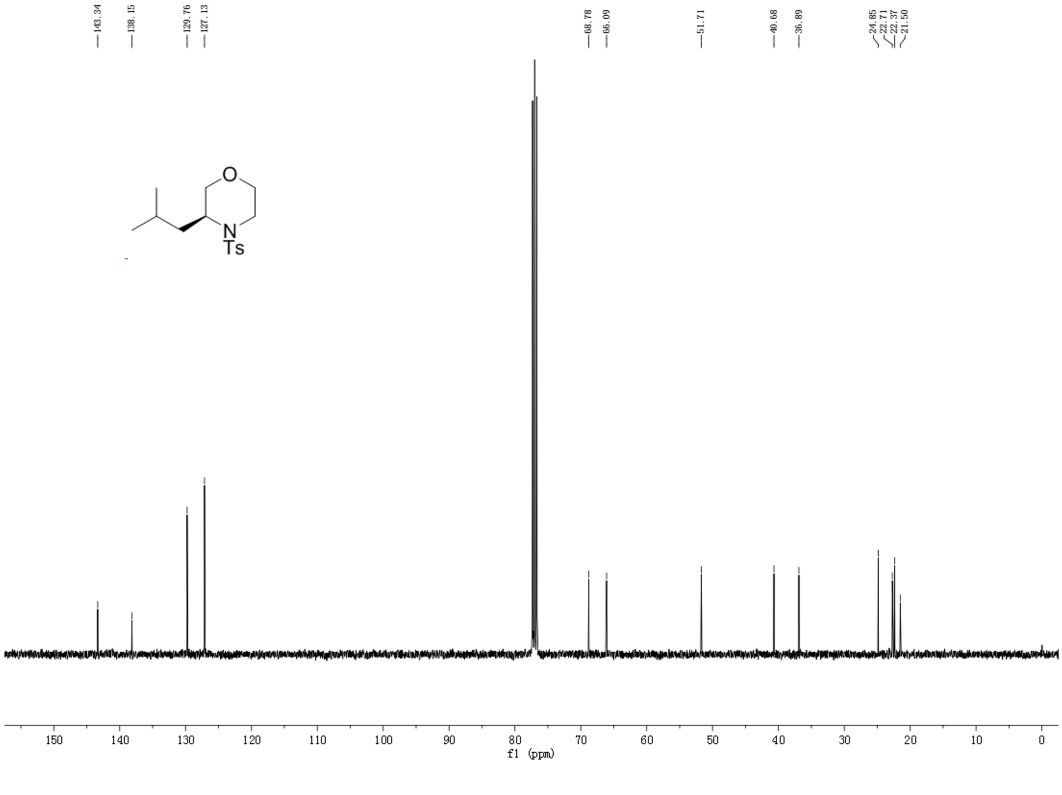

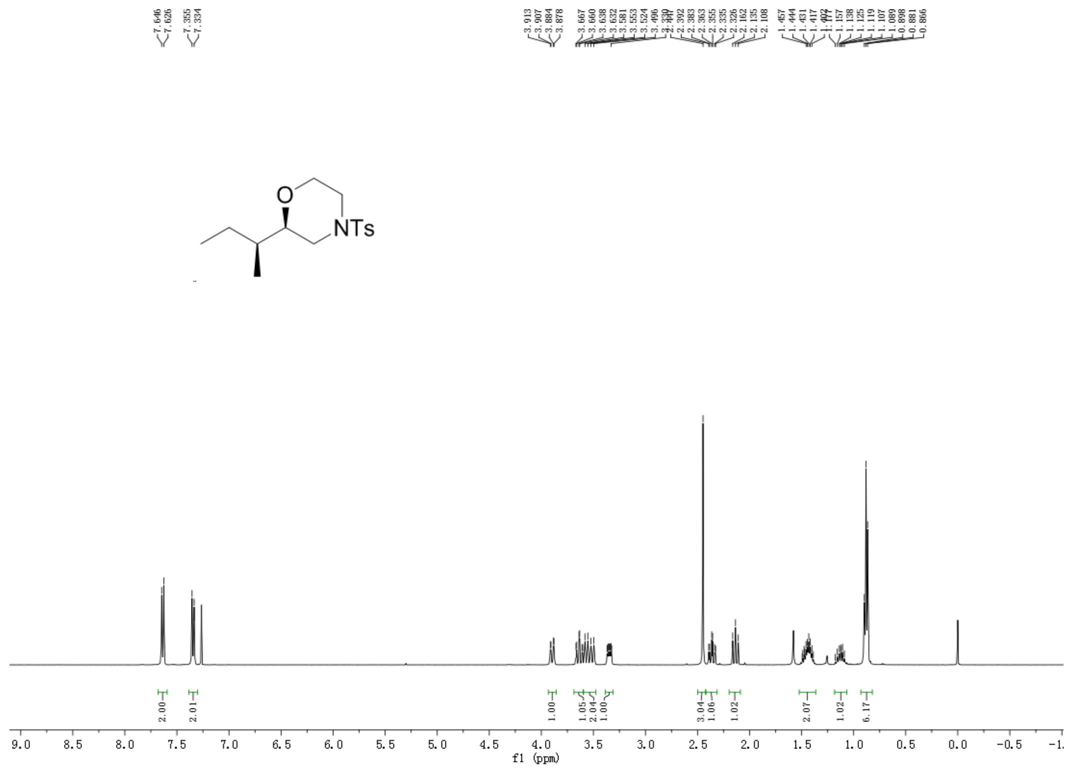
NTs
O
f1 (ppm)

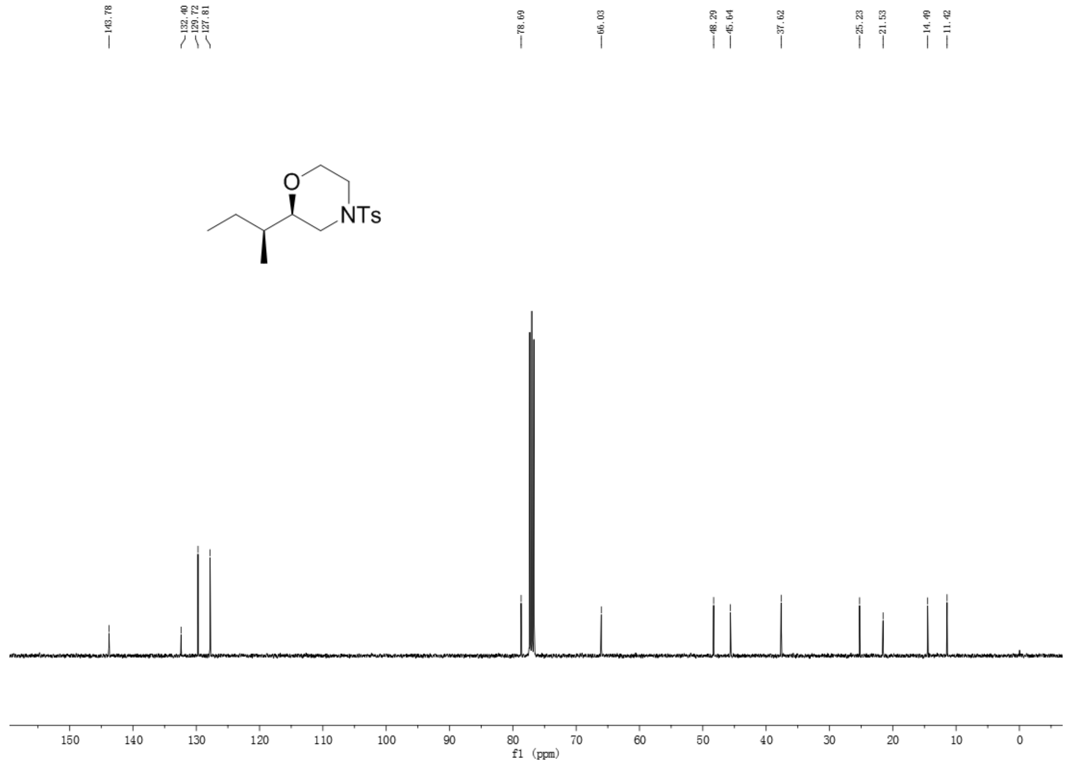
NTs
O
f1 (ppm)

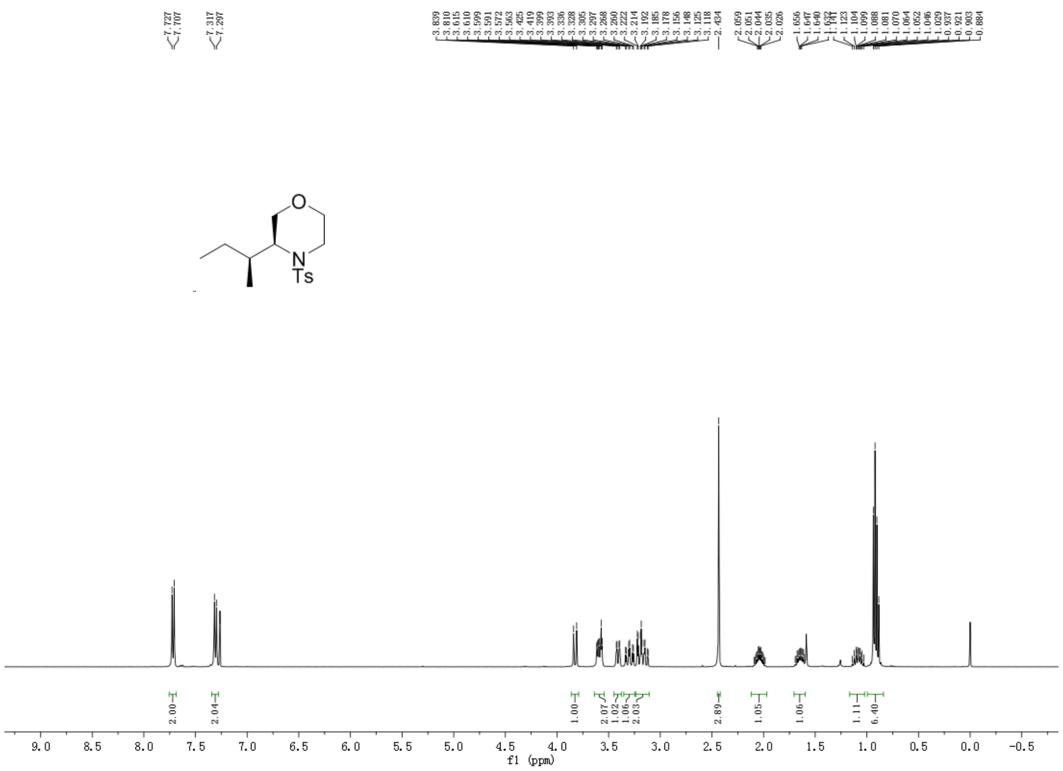

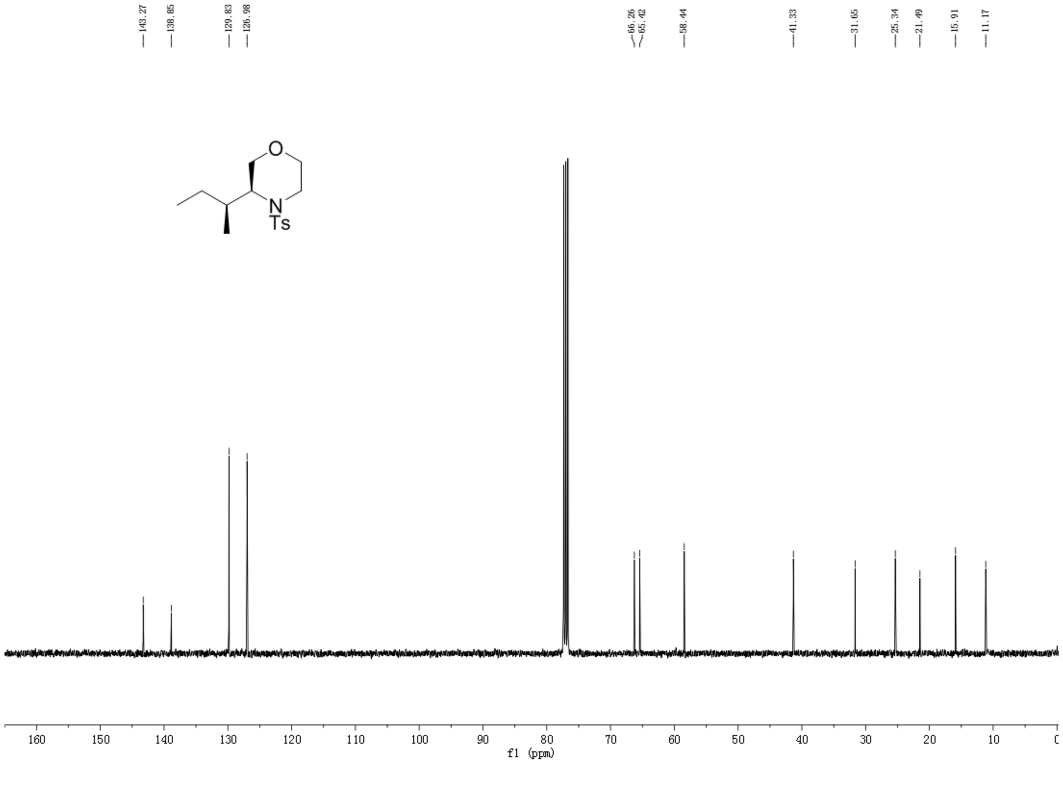

Supplement: File 1 — Experimental procedures, characterization data and copies of 1H and 13C NMR spectra for products. [file Beilstein_J_Org_Chem-11-524-s001.pdf]
